# Supplementary material for: A unified framework for unconstrained and constrained ordination of microbiome read count data
Source: PLoS One. 2019 Feb 13;14(2):e0205474. doi: 10.1371/journal.pone.0205474 (PMC6373939; doi:10.1371/journal.pone.0205474)
Supplement: S1 Appendix — Further, a detailed description of the setup and results of the simulation study, followed by a list of software versions. (PDF) [file pone.0205474.s001.pdf]

A unified framework for unconstrained and constrained ordination of microbiome read count data

Supplementary material

Stijn Hawinkel[1], Frederiek-Maarten Kerckhof[2], Luc Bijnens[3,4], Olivier Thas[1,4,5]*
1 *Department of Data Analysis and Mathematical Modelling, Ghent University, Belgium*
2 *Center for Microbial Ecology and Technology, Ghent University, Belgium*
3 *Quantitative Sciences, Janssen Pharmaceutical companies of Johnson and Johnson, Belgium*
4 *Center for Statistics, Hasselt University, Belgium*
5 *National Institute for Applied Statistics Research Australia (NIASRA), University of Wollongong, Australia*
[*] *Corresponding author; stijn.hawinkel@ugent.be*

Contents

1 Introduction **2**

2 Method description **2**
2.1 Fitting procedure . . . 2
2.1.1 Inputs . . . 2
2.1.2 Trimming . . . 3
2.1.3 Independence model . . . 3
2.1.4 Conditioning on confounders . . . 4
2.1.5 Capturing the signal . . . 4
2.2 Explanatory notes . . . 8
2.2.1 Estimating the independence model . . . 8
2.2.2 The choice of normalization weights . . . 9
2.2.3 Shape of the response function . . . 11
2.2.4 Relationship between unconstrained RC(M) and existing methods . . . 11
2.2.5 Relationship between constrained RC(M) and existing methods . . . 14
2.3 Plotting the RC(M) ordination . . . 14
2.3.1 Unconstrained RC(M) . . . 14
2.3.2 Constrained RC(M) . . . 14
2.4 Assessing the model quality . . . 15
2.4.1 Parsimony . . . 15
2.4.2 Importance of the dimension . . . 16
2.4.3 Detecting lack of fit . . . 17
2.4.4 Identifying influential observations . . . 17

3 Simulation study **19**
3.1 Parametric simulations . . . 19
3.1.1 Summary table of the simulation scenarios . . . 20
3.1.2 Overview of the parametric simulation workflow . . . 20
3.2 Nonparametric simulation . . . 22
3.3 Automatic method evaluation . . . 22
3.3.1 Robustness to technical artefacts . . . 22
3.3.2 Sample separation . . . 22
3.3.3 Contribution of taxa to the separation of the clusters . . . 23

3.4 Results of simulation study . . . 24
3.4.1 No-signal simulations . . . 24
3.4.2 Biological signal simulations . . . 34
3.4.3 Some validation plots . . . 36
3.5 Computational benchmark . . . 44
3.6 Failed fits . . . 44
3.7 Summary table . . . 44

4 Real data examples 45
4.1 Human microbiome project . . . 45
4.2 The American gut project . . . 48
4.3 Turnbaugh et al. (2009) . . . 49
4.4 Zeller et al. (2014) . . . 50
4.4.1 Unconstrained RC(M) . . . 50
4.4.2 Constrained RC(M) . . . 52
4.4.3 Diagnostic plots . . . 57
4.5 Kostic et al. (2012) . . . 60
4.6 Props et al. (2016) . . . 65

5 R-code 69

6 R-language and package versions 69

7 Hardware specifications 71

1 Introduction

This document provides an exact description of the algorithm used to fit the RC(M) model augmented with the negative binomial distribution for microbiome data. Further it embeds the method in the existing literature, and points out correspondences and differences with existing methods. A simulation study is set up to compare the performance of the RC(M) method to competitor methods, and computational benchmarking comparison of the different methods is given. Next the method is illustrated on some real datasets. Finally the R-code used to make the graphs in the paper is given, as well as some version info of the software and hardware used.

The paper comes with an R-package called *RCM* which can be found at https://github.com/CenterForStatistics-UGent/RCM, together with a basic manual. More advanced instructions for use can be found at http://users.ugent.be/~shawinke/RCMmanual/.

2 Method description

2.1 Fitting procedure

2.1.1 Inputs

The algorithm requires the following inputs:

- an n×p data matrix $\mathbf{X}$, with samples i in the rows and taxa (species, OTUs) j in the columns. Thus x_{ij} is the observed count of taxon j in sample i.
- the required dimension of the solution (M). The dimensions are fitted sequentially.

The algorithm allows to supply the following optional inputs:

- a n×k design matrix **G** of confounding variables. Factor variables are coded with 0/1 dummies. The first column should have all elements equal to one (intercept). The entry g_{il} then represents the value of confounder variable l in sample i.
- a n×d design matrix **C** of constraining variables. Factor variables are coded with 0/1 dummies. No intercept is included. The entry c_{iy} then represents the value of constraining variable y in sample i.

2.1.2 Trimming

Rows and columns of **X** with only zero counts are trimmed prior to model fitting. To avoid numerical instability, also taxa below a certain prevalence threshold, or with total count lower than a certain fraction of the number of samples n, are excluded prior to model fitting. The default prevalence threshold is 5%, the default fraction of n is 10%.

If a confounder matrix is provided with dummy variables, also discard the taxa that fall below the prevalence and total count fractions (mentioned above) within each level of the categorical confounding variables, again to avoid overflow.

2.1.3 Independence model

The independence model of sample homogeneity has mean model

$$\log(E(X_{ij})) = u_i + v_j$$

and is augmented with the negative binomial distribution with taxon-specific dispersion parameters θ_j. The independence model is fitted as follows (see Section 2.2.1 below for explanation):

1. Find starting values $u_{i,init} = \log\left(\sum_{j=1}^{p} x_{ij}\right)$ and $v_{j,init} = \log(\frac{\sum_{i=1}^{n} x_{ij}}{\sum_{i=1}^{n}\sum_{j=1}^{p} x_{ij}})$ for u_i and v_j respectively
2. Estimate a mean-dispersion trend using the estimateGLMTrendedDisp() function of the *edgeR* package (version 3.24.3) (Robinson et al. 2010), given u_i and v_j. This estimate is relatively insensitive to slight changes in the mean structure and will only be re-estimated once for every dimension of the ordination to save computation time.
3. Estimate the dispersion parameters θ_j based on the mean-dispersion trend using empirical Bayes with the estimateGLMTagwiseDispersion() function in the *edgeR* package, given u_i and v_j. This step is only executed every 10 iterations (starting at the first iteration) to save computation time.
4. Estimate new values for u_i ($u_{i,new}$) using maximum likelihood (ML), keeping the θ_j's and v_j's constant.
5. Estimate new values for v_j ($v_{j,new}$) using ML, keeping the θ_j's and u_i's constant
6. Check for convergence. If no convergence is reached, repeat steps 3-5. Convergence is assumed when

$$\sqrt{\left(\frac{1}{n}\sum_{i=1}^{n}(1-\frac{u_{i,new}}{u_{i,old}})^2\right)} < 0.001$$

and

$$\sqrt{\left(\frac{1}{p}\sum_{j=1}^{p}(1-\frac{v_{j,new}}{v_{j,old}})^2\right)} < 0.001$$

Once the independence model has converged, the estimates u_i and v_j are kept constant throughout the remainder of the fitting process. The dispersion estimates will still be re-estimated as nuisance parameters in further steps. All maximum likelihood estimation occurs under the negative binomial model.

2.1.4 Conditioning on confounders

If a confounder matrix is provided, the effect of the confounders is filtered out by fitting the following mean model (using maximum likelihood):

$$\log(E(X_{ij})) = u_i + v_j + \sum_{l=1}^{k} \zeta_{jl} g_{il}$$

with ζ_{jl} the interaction parameter between taxon j and confounding variable l. Note that $g_{i1} = 1$ for all i, i.e. the model is fitted with an intercept.

Again this step is performed iteratively by alternating between estimating the ζ_{jl} parameters and re-estimating the overdispersion parameters as in step 3 of the independence model. Convergence is then assumed when $\sqrt{\frac{1}{pq}\sum_{l=1}^{k}\sum_{j=1}^{p}(1-\frac{\zeta_{jl,new}}{\zeta_{jl,old}})^2}$ drops below a tolerance level of 0.001.

2.1.5 Capturing the signal

The steps undertaken so far to model $E(X_{ij})$ are merely fitting a "null" model and will not play a role in the final ordination. The next terms that will be added will capture the biological signal in the data $\mathbf{X}$ and will be used for data visualization. This step differs between an *unconstrained* RC(M) model, that merely uses the data $\mathbf{X}$, and *constrained* analysis, that also incorporates the covariate matrix $\mathbf{C}$.

2.1.5.1 Unconstrained RC(M)

The unconstrained RC(M) model has mean structure

$$\log(E(X_{ij})) = u_i + v_j + \left[\sum_{l=1}^{k} \zeta_{jl} g_{il}\right] + \sum_{m=1}^{M} \psi_m r_{im} s_{jm}$$

with the term between [] for conditioning on known confounders being optional.

The unconstrained RC(M) model is fitted as follows:

1. Obtain a singular value decomposition as $\mathbf{R}^{-1}(\mathbf{X}-\mathbf{E})\mathbf{J}^{-1} = \mathbf{U\Sigma V}^T$, with $\mathbf{E}$ the matrix of fitted values from the previous step. The matrices $\mathbf{R}$ and $\mathbf{J}$ are diagonal matrices with row and column sums of $\mathbf{X}$ on the diagonal, respectively. The first M elements of the ith row of U, denoted by $r_{i1}^{\text{SVD}}, \ldots, r_{iM}^{\text{SVD}}$, give the initial estimates of the r_{im} parameters. Similarly, $\mathbf{\Sigma}$ and $\mathbf{V}$ give initial estimates for the $\boldsymbol{\psi}_m$ and $\mathbf{s}_m$ parameters. For these initial values we still need to ensure that the (weighted) variances equal 1. We do this by transferring some weight to the importance parameters ψ^{SVD} by setting:

$$\psi_m^{init} = \psi_m^{SVD}\sqrt{\sum_{i=1}^{n}\left(r_{im}^{SVD}\right)^2 \sum_{j=1}^{p}\left(z_j s_{jm}^{SVD}\right)^2}$$

$$r_{im}^{init} = \frac{r_{im}^{SVD}}{\left(\sum_{i=1}^{n}\left(r_{im}^{SVD}\right)^2\right)^{1/2}}$$

and

$$s_{jm}^{init} = \frac{z_j s_{jm}^{SVD}}{\left(\sum_{j=1}^{p}\left(s_{jm}^{SVD} z_j\right)^2\right)^{1/2}}$$

with $z_j = \exp(v_j)$ a taxon weight, see Section 2.2.2 below for an extended discussion on the weights.

2. For all dimensions m starting from 1 to M, the following steps are executed:

a) Estimate the dispersions θ_j using empirical Bayes as before (every tenth iteration)
b) Estimate the importance parameter ψ_m by full ML, forcing it to be positive and keeping the sample and taxon scores and overdispersions fixed.
c) Estimate the sample scores r_{im} by restricted ML, keeping the taxon scores, overdispersions and importance parameters fixed. Lagrangian multipliers in the log-likelihood are used to ensure that

$$\sum_{i=1}^{n} r_{im} = 0$$

and

$$\sum_{i=1}^{n} r_{im} r_{im'} = \delta_{mm'}$$

with δ the Kronecker delta.

c) Estimate the taxon scores s_{jm} by restricted ML, keeping the sample scores, overdispersions and importance parameters fixed. Lagrange multipliers in the log-likelihood are used to ensure that

$$\sum_{j=1}^{p} z_j s_{jm} = 0$$

and

$$\sum_{j=1}^{p} z_j s_{jm} s_{jm'} = \delta_{mm'}$$

d) Check for convergence. If no convergence reached, repeat steps a-c, otherwise move to the next dimension and start again from (a), conditioning on the estimates of previous dimensions. Convergence for dimension m is assumed when

$$\left|1 - \frac{\psi_m^{new}}{\psi_m^{old}}\right| < 0.001$$

and

$$\sqrt{\frac{1}{n}\sum_{i=1}^{n}(1 - \frac{r_{im}^{new}}{r_{im}^{old}})^2} < 0.001$$

and

$$\sqrt{\frac{1}{p}\sum_{j=1}^{p}(1 - \frac{s_{jm}^{new}}{s_{jm}^{old}})^2} < 0.001.$$

The Lagrangian parameters are stored to be used as starting values in the next iteration so as to speed up the computation.

2.1.5.2 Constrained RC(M)

The constrained RC(M) model has mean structure

$$\log(E(X_{ij})) = u_i + v_j + \Big[\sum_{l=1}^{k} \zeta_{jl} g_{il}\Big] + \sum_{m=1}^{M} \psi_m f_{jm}(\boldsymbol{\alpha}_m^t \mathbf{c}_i)$$

with the term between [] for conditioning on known confounders being optional. For this model four components need to be fitted iteratively:

- θ_j, the overdispersion parameter as before
- ψ_m, the importance parameter as in the unconstrained model
- $\boldsymbol{\alpha}_m$, the environmental gradients, under the restriction

$$\boldsymbol{\alpha}_m^t \boldsymbol{\alpha}_{m'} = \delta_{mm'}$$

- f_{jm}, the species specific response function. This can be parametric (polynomial in practice) or non-parametric.

The fitting of the constrained RC(M) model proceeds as follows:

1. Standardize the covariate matrix **C**. To render the values of the continuous variables in the environmental gradient comparable, it is clear that they need to be centered and scaled prior to model fitting, as in PCA. This means that their corresponding elements of $\boldsymbol{\alpha}$ represent the contribution to the environmental score of one standard deviation away from the mean of this variable. A perfect quantitative comparison to the magnitude of the parameters of the categorical variables will never be possible. In our case, with 0/1 dummy coding, equal parameters for a dummy and a continuous variable imply that this level of the categorical variable contributes as much to the environmental score as one standard deviation away from the overall mean of the continuous variable.

2. Starting values for $\boldsymbol{\alpha}$ are obtained from a constrained correspondence analysis by the *cca*() function in the *vegan* package (Oksanen et al. 2017). Next, they are normalized to fulfill the $\boldsymbol{\alpha}_m^t \boldsymbol{\alpha}_m = 1$ requirement by setting

$$\boldsymbol{\alpha}_m = \frac{\boldsymbol{\alpha}_m^{cca}}{\sqrt{(\boldsymbol{\alpha}_m^{cca})^t \boldsymbol{\alpha}_m^{cca}}}$$

Starting values for $\boldsymbol{\psi}$ are the eigenvalues of the constrained correspondence analysis. For the response functions no starting values are calculated.

3. For all M, the following steps are performed for dimension m = 1 up to m = M:
    a) Estimate the overdispersions by empirical Bayes as before (every tenth iteration).
    b) If the response function is parametric, estimate the importance parameter ψ_m by full ML
    c) Estimate the response functions f_{jm} by full ML. For **parametric response functions** this entails estimating a vector of parameters $\left(\beta_{0jm}, ..., \beta_{vjm}\right)$ with v the degree of the polynomial. After fitting, normalize these parameters to (w = 1,...,v)

$$\beta_{wjm}^{new} = \frac{\beta_{wjm}}{\sqrt{\sum_{j=1}^{p} \beta_{wjm}^2}}.$$

This assures $\boldsymbol{\beta}_{wm}^t \boldsymbol{\beta}_{wm} = 1$. Note that we do not weigh the taxon-wise components by z_j here, to avoid extreme solutions. See Section 2.2.2 below for an extended discussion on weighting.

For **non-parametric response functions**, estimation of the response functions relies on cubic splines as defined by the $s()$ function of the $VGAM$ package (Yee 2015). For these models, the importance parameter ψ_m is not estimated during the model fitting process, but calculated afterwards. It plays no role in the plotting but is just a measure of importance of each dimension. It is defined to be

$$\psi_m = \sqrt{\frac{1}{np}\sum_{j=1}^{p}\sum_{i=1}^{n} f_{jm}(h_{im})^2}$$

Analogously, estimate general response functions f_m ignoring species labels.

d) Estimate the environmental gradient $\boldsymbol{\alpha}_m$ by maximizing the logged likelihood ratio

$$LR(\boldsymbol{\alpha}_m) = \log \frac{\prod_{i=1}^{n}\prod_{j=1}^{p} g_{NB}(x_{ij}; \boldsymbol{\alpha}_m^t \mathbf{c}_i, f_{jm}, \theta_j, \psi_m)}{\prod_{i=1}^{n}\prod_{j=1}^{p} g_{NB}(x_{ij}; \boldsymbol{\alpha}_m^t \mathbf{c}_i, f_m, \theta_j, \psi_m)}$$

with g_{NB} the density function of the negative binomial distribution, and under the restrictions that:

- $\boldsymbol{\alpha}_m^t \boldsymbol{\alpha}'_m = 0$ for m $\neq$ m'
- Components of α_m belonging to dummies of the same categorical variable sum to zero

The latter restriction is convenient for later plotting and also avoids dependence of the solution on the choice of reference level, in the light of the normalization step in e). This way of estimating $\boldsymbol{\alpha}$ encourages maximal niche separation between the species.

However, the separated niche concept is not accepted by all ecologists. If niches are really maximally separated, how can species co-occur then? An alternative option is therefore to estimate $\boldsymbol{\alpha}_m$ by maximizing only the numerator in the previous equation, but under the same restriction. Surprisingly, the solutions of both approaches are very similar, although not exactly identical. Maximizing the numerator is also much faster.

e) Set

$$\boldsymbol{\alpha}_m^{new} = \frac{\boldsymbol{\alpha}_m}{\sqrt{\boldsymbol{\alpha}_m^t \boldsymbol{\alpha}_m}}$$

to normalize the gradient.

f) Check for convergence. If no convergence is reached, repeat steps a-e, otherwise move to the next dimension and start again from (a), conditioning on the estimates for the previous dimensions. Convergence is assumed when

$$\left|1 - \frac{\psi_m^{new}}{\psi_m^{old}}\right| < 0.001$$

and

$$\sqrt{\frac{1}{d}\sum_{l=1}^{d}(1 - \frac{\alpha_{lm}^{new}}{\alpha_{lm}^{old}})^2} < 0.001$$

For parametric response functions there is an additional requirement that

$$\sqrt{\frac{1}{pv}\sum_{j=1}^{p}\sum_{w=1}^{v}\left(1 - \frac{\beta_{wjm}^{new}}{\beta_{wjm}^{old}}\right)^2} < 0.001$$

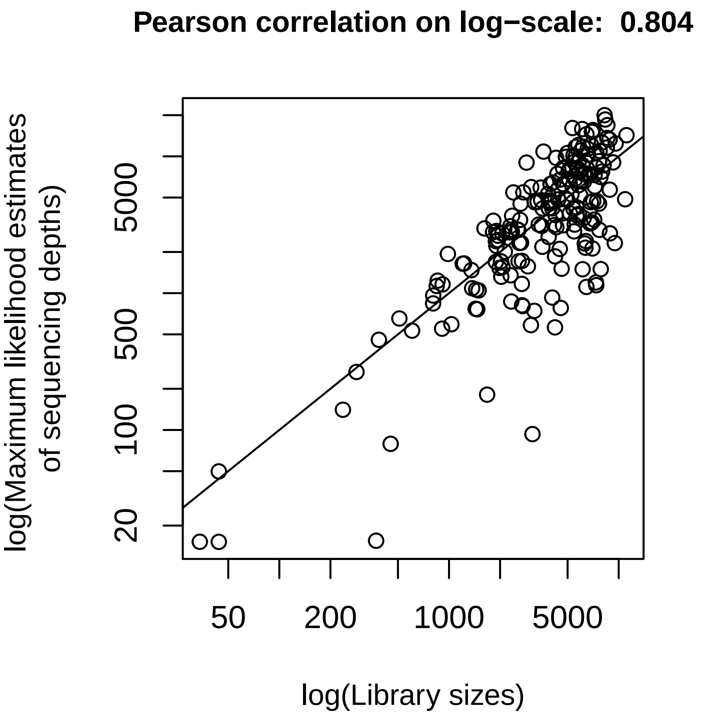

Figure S1: Scatterplots on the log-scale of row sums (library sizes) versus maximum likelihood estimates of sequencing depth for the Kostic dataset.

2.2 Explanatory notes

In this section we give some explanation regarding the particularities of this fitting procedure with respect to the original method by Goodman (1979).

2.2.1 Estimating the independence model

An independence model for a contingency table is basically a marginal model. Therefore the most obvious, model free way to estimate the independence model may be simply through sample and taxon sums, namely $u_i = \log(x_{i.})$ with $x_{i.} = \sum_{j=1}^{p} x_{ij}$ and $v_j = \log(\frac{x_{.j}}{x_{..}})$ with $x_{.j} = \sum_{i=1}^{n} x_{ij}$ and $x_{..} = \sum_{j=1}^{p}\sum_{i=1}^{n} x_{ij}$.

However, the library sizes $x_{i.}$ do not correspond to the maximum likelihood estimate of $\exp(u_i)$ under the negative binomial model. As a result the first dimensional row scores r_{1i} would try to correct for this discrepancy and become related (linearly correlated) to the library sizes. This effect of sequencing depth on the sample ordination is something we want to avoid absolutely. Therefore we estimate the u_i's and v_j's iteratively using maximum likelihood, which also implies dispersion estimation as outlined above.

In practice, the marginal sums differ more from the maximum likelihood estimate (MLE) for the library sizes than for the taxon abundances, as can be seen from Supplementary Figures S1 and S2.

We can think of the following mathematical explanation: when calculating library sizes or abundances by row and column sums, each observations receives the same weight. However, when we estimate the margins through ML, we solve the following score equations:

$$\frac{\partial L(\mathbf{X}|\mathbf{u}_i, \mathbf{v}_j, \boldsymbol{\theta})}{\partial u_i} = \sum_{j=1}^{p} \frac{x_{ij} - \mu_{ij}}{1 + \frac{\mu_{ij}}{\theta_j}} = 0$$

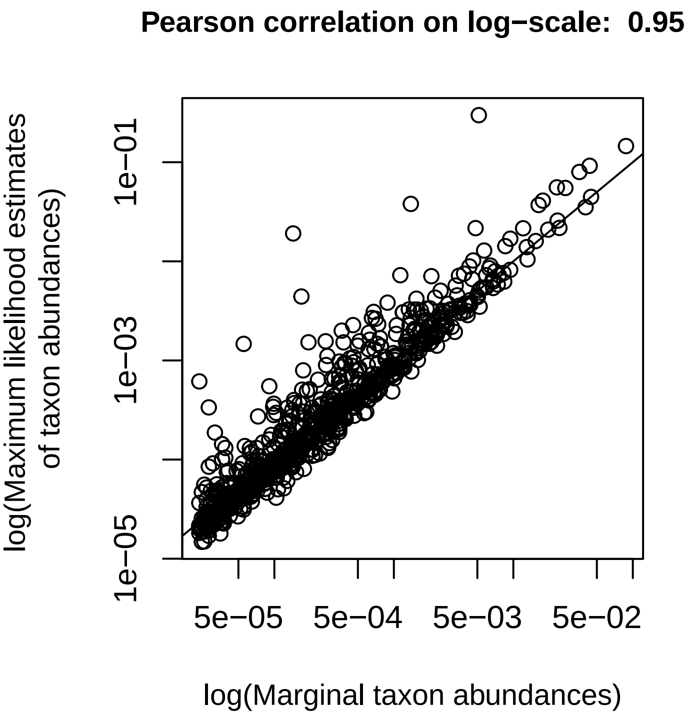

Figure S2: Scatterplots on the log-scale of column sums (marginal taxon abundances) versus maximum likelihood estimates of abundance respectively for the Kostic dataset.

$$\frac{\partial L(\mathbf{X}|\mathbf{u}_i, \mathbf{v}_j, \boldsymbol{\theta})}{\partial v_j} = \sum_{i=1}^{n} \frac{x_{ij} - \mu_{ij}}{1 + \frac{\mu_{ij}}{\theta_j}} = 0,$$

with L representing the log-likelihood of the negative binomial distribution. Note that since we are estimating offsets the value of the regressor is always 1. In this case the difference of x_{ij} with the expected value μ_{ij} is weighted by a factor $\frac{1}{1+\frac{\mu_{ij}}{\theta_j}} = \frac{\theta_j}{\theta_j+\mu_{ij}}$. When estimating v_j, θ_j is a constant but when estimating u_i it is different for every observation x_{ij}. Hence the weights put on every observation differ much more when estimating the sample offsets than when estimating the taxon offsets. That is the reason why the MLE differs more from the marginal sum for the library size than for the abundances.

Note also that when there is a very large overdispersion for a taxon j (θ_j small), its observations carry little information and their weights are small in the calculation of the library sizes. However, when there is very little overdispersion ($\theta_j \to \infty$), the weights of the components of the score function equal 1, as with Poisson regression. It is thus not surprising that the MLEs of the Poisson regression are equal to the estimators based on the marginal sums. This means that the larger and the more diverse the overdispersion estimates are, the more the MLEs under the negative binomial model will depart from the marginal sums. Finally, we see that departures from the mean μ_{ij} are weighted down for large values of μ_{ij}, acknowledging the fact that the variance increases faster than linear with the mean in the negative binomial model.

2.2.2 The choice of normalization weights

Constraints are needed to render the RC(M) model identifiable. We restrict the importance parameter ψ_m to be positive, and center the (weighted) row and column scores around 0, which is a useful property for the biplot and are also the restrictions imposed for correspondence analysis. In addition the row and column scores are restricted to have (weighted) variance 1. ψ_m is the only parameter that can grow in size

without restriction. As a result it will automatically serve as a measure of importance of the departure from independence in that direction. This is also the case for correspondence analysis. Thirdly, the scores of the different dimensions are orthogonal, so the solutions in different dimensions are linearly independent. Details of the restrictions are given through the following equations.

Centering:

$$\sum_{i=1}^{n} w_i r_{im} = 0$$

$$\sum_{j=1}^{p} z_j s_{jm} = 0$$

Normalization($m = m'$) and orthogonality ($m \neq m'$):

$$\sum_{i=1}^{n} w_i r_{im} r_{m'i} = \delta_{mm'}$$

$$\sum_{j=1}^{p} z_j s_{jm} s_{m'j} = \delta_{mm'}$$

In these expressions w_i and z_j are row and column weights. Goodman proposes to use $w_i = x_{i.}$ and $z_j = x_{.j}$. This results in *weighted* constraints that retain the relationship with correspondence analysis (Goodman 1979). Others recommend using uniform weights not to let the marginal distribution affect the model fit (Becker and Clogg 1989).

To make the correct choice one should remember that the weights can be regarded as probabilities, representing the likelihood of sampling a certain sample or taxon from the population. On the population level we could say that

$$E_w(R_m) = \sum_{i=1}^{n} w_i r_{im} = 0$$

i.e. the average row score on the population level is zero. This is a useful restriction to make sure that the biplot is centered around zero. Analogously we want that

$$E_w(R_m R_{m'}) = \sum_{i=1}^{n} w_i r_{im} r_{im'} = \delta_{mm'}$$

and accordingly for the column scores:

$$E_z(S_m) = \sum_{j=1}^{p} z_j s_{jm} = 0$$

$$E_z(S_m S_{m'}) = \sum_{j=1}^{p} z_j s_{jm} s_{jm'} = \delta_{mm'}$$

For the microbiome case, every subject comes from the same population under the null-hypothesis and all subjects are thus equally likely to be sampled and have the same importance. The library sizes are considered as technical artefacts, which are unrelated to the biological importance of the subject. Consequently we use

uniform row weights $w_i = 1/n$ (or $w_i = 1$, the magnitude is of no importance since the associated ψ_m will grow or shrink accordingly). However, some taxa are more prevalent in the population than others. We want the average column scores on the population level to be centered around zero, have variance one and be orthogonal. That is why we set $z_j = \exp(v_j)$; because the more abundant species are in fact more abundant in the population as a whole (as opposed to samples with a large library size), it makes sense to use a marginal weighting scheme for the column scores. The weights z_j are derived from the independence model.

For the parameters of the parametric response functions we use uniform weights for normalization, because the use of $\exp(v_j)$ as weights for the normalization leads to very extreme solutions. Likely this is because the parameters are not centered.

2.2.3 Shape of the response function

Note that our definition of the response function differs from the common definition (ter Braak 1986; Zhu et al. 2005; Yee 2006), which models the mean abundance as a function of environmental conditions. Here the response function models the mean departure from sample homogeneity.

A linear response function may be most appropriate for problems with **short gradients** i.e. whereby the difference in observed environmental variables is too short to distinguish more than an increase or decrease in abundance. Also in this case it is easy to interpret the effect of each of the environmental variables on the departure from homogeneity. As so often in statistics, the linearity assumption may not be realistic, but renders models that are easy to interpret.

For problems with **long gradients** for which species' departures of homogeneity may not be monotonic within the scope of the observed environmental scores, quadratic response functions may be more appropriate. This corresponds e.g. with the scenario in which a species' abundance does not depart heavily from homogeneity for extreme values of the environmental score, but does depart heavily for an intermediate value of the environmental score. In essence this is the same as the approach from Zhu et al. (2005), only now the baseline is the homogeneity model rather than 0. Every taxon thereby has its own baseline (the taxon's mean abundance), and the response function models departures from this baseline. Note that we usually do not choose the ranges of the environmental variables or scores, so that we cannot guarantee a range long enough for the quadratic response function to be appropriate.

Even though the quadratic response function can be fitted, it may still be pointless if the maximum lies outside the range of the observed values for the environmental score. The peak location would then merely be an extrapolation, and a linear response function may be preferable. Even though the linear fit is worse than for the parabolic curve, it represents more truthfully the way the species reacts to the given values of the environmental gradient. Therefore we also provide a "dynamic"-option for the response function, whereby initially a quadratic model is fitted but discarded in favour of a linear one if the optimum lies outside of the range of observed environmental scores. For plotting it is not very attractive to have different shapes of the response function for the same taxon in different dimensions though. Another drawback of quadratic response functions is that its maximum likelihood solution may take on a convex shape, which is hard to give a biological meaning (Zhang and Thas 2016). Additionally the quadratic response function may be convex in some dimensions and concave in others, further blurring the interpretation.

If the user is unsure and has enough data, he may use non-parametric response functions. This may improve the sample and covariate ordination, and makes far less assumptions on the shape of the response function (apart from a certain smoothness). This approach is very interesting if one wants to study individual taxa's response functions.

All in all we see a trade-off between flexibility of the response function and interpretability of the role of the taxa.

2.2.4 Relationship between unconstrained RC(M) and existing methods

2.2.4.1 Correspondence analysis

2.2.4.1.1 Independence model

Under independence between rows and columns we model the counts in a contingency table as

$$E(X_{ij}) = a_i b_j$$

whereby usually $a_i = x_{i.} = \sum_{j=1}^p x_{ij}$ (the library sizes) and $b_j = \frac{x_{.j}}{x_{..}} = \frac{\sum_{i=1}^n x_{ij}}{\sum_{j=1}^p \sum_{i=1}^n x_{ij}}$ (the average relative abundances). Let $\mathbf{E} = \mathbf{a}\ \mathbf{b}^T$ denote the $n \times p$ matrix of with the expected counts under independence.

There exist many variations of correspondence analysis, but all are concerned with the difference between the observed count $\mathbf{X}$ and the expected counts based on the margins (the independence model) $\mathbf{E}$. This means that the signal can come from observations that are either smaller or larger than expected.

2.2.4.1.2 Reconstitution formula of Correspondence Analysis

A more extended model than the independence model is

$$E(X_{ij}) = a_i b_j \Big(1 + \sum_{m=1}^{M} \omega_m q_{im} t_{jm}\Big)$$

with $a_i = x_{i.} = \sum_{j=1}^p x_{ij}$ and $b_j = \frac{x_{.j}}{x_{..}}$ with $x_{.j} = \sum_{i=1}^n x_{ij}$. M = min(n,p) and the terms are ordered such that $\omega_1 > \omega_2 > ... > \omega_M$.

When fitted, the second series of terms will attempt to repair discrepancies between $\mathbf{X}$ and $\mathbf{E}$ and as such capture departures from independence. This is called the *reconstitution formula* since it decomposes the observed average count into its expectation under independence and a residual. The residual is then further decomposed into M orthogonal pieces.

Correspondence analysis is usually done through singular value decomposition (SVD) of the matrix of departures from independence **X-E**. However, this is not directly applied to the matrix of raw departures, but rather to the residual matrix weighted by row and column scores in one way or the other, to account for the heteroscedasicity of count data. Subtle differences in choice of weights lead to different versions of correspondence analysis. Very often it is not mentioned which version is used, which complicates comparison of results of different packages.

The most common form of correspondence analysis performs a singular value decomposition of the following matrix

$$\mathbf{A_1} = \mathbf{R^{-1/2}}(\mathbf{X} - \mathbf{E})\mathbf{J^{-1/2}} = \mathbf{U\Sigma V}^T$$

with $\mathbf{\Sigma}$ a diagonal matrix with the singular values of $\mathbf{X}$ (all between 0 and 1) on the diagonal and $\mathbf{R}$ and $\mathbf{J}$ diagonal matrices with row and column sums of $\mathbf{X}$.

Elements of this matrix are

$$a_{1ij} = \frac{x_{ij} - x_{i.}x_{.j}/n}{\sqrt{x_{i.}x_{.j}}} = \frac{1}{\sqrt{n}} \frac{x_{ij} - x_{i.}x_{.j}/n}{\sqrt{x_{i.}x_{.j}/n}}$$

which can be recognized as $\sqrt{n}$ times the Pearson standardized residuals. The Pearson standardized residuals standardize the departures from $\mathbf{E}$ by dividing by the square root of the expected value. This is in line with the common assumption that for count data $E(X_{ij}) = \text{Var}(X_{ij})$. This transformation would yield approximately standard normally distributed variables if this assumption holds and $E(X_{ij})$ is sufficiently large. Hence the sum of squared elements

$$\sum_{i=1}^{n} \sum_{j=1}^{p} a_{1ij}^2$$

yields 1/n times the Pearson χ^2 statistic to test for association in the contingency table.

In matrix notation the reconstitution formula becomes

$$X = E_{independence} + R^{1/2} U \Sigma V^T J^{1/2}$$

with $\mathbf{1}^T \mathbf{R}^{1/2} \mathbf{U} = \mathbf{0}$ and $\mathbf{1}^T \mathbf{J}^{1/2} \mathbf{V}^T = \mathbf{0}$ (weighted means of rows and columns equal zero) and $\mathbf{U}^T \mathbf{R}^{1/2} \mathbf{U} = \mathbf{1}$ and $\mathbf{V}^T \mathbf{K}^{1/2} \mathbf{V} = \mathbf{1}$ (weighted variances equal one) (Heijden and Leeuw 1985).

2.2.4.1.3 Link to the RC(M)-model

If $a = \sum_{m=1}^{M} \omega_m v_{im} w_{jm}$ is small (i.e. the deviation from independence is small) then $log(1+a) \approx a$ and

$$\log(E(x_{ij})) = \log(x_{i.}) + \log(x_{.j}) - \log(x_{..}) + \log\left(1 + \sum_{m=1}^{M} \omega_m q_{im} t_{jm}\right) \approx \log(x_{i.}) + \log(x_{.j}) - \log(x_{..}) + \sum_{m=1}^{M} \omega_m q_{im} t_{jm}$$

which shows a relationship between the RC(M)-model and correspondence analysis (Escoufier 1982; Heijden et al. 1994).

If the same restrictions apply to the scores q_{im} and t_{jm} as to $\mathbf{U}$ and $\mathbf{V}$, we can state that $\psi_m \approx \omega_m$, $q_{im} \approx r_{im}$ and $t_{jm} \approx s_{jm}$. The assumption that the departure from independence is small seems unlikely for microbiome data, but it does provide useful starting values for the ML fitting of the RC(M) model.

2.2.4.2 Gomms

The *gomms* package (Sohn and Li 2017) implements a similar mean model to the RC(M) model, but the differences lie mainly in the error distribution. Our RC(M) model assumes a negative binomial model with unique dispersion parameters for every taxon. The *gomms* methods employs a zero-inflated quasi Poisson distribution with a common overdispersion parameter. It is well known however, that overdispersions differ a lot between taxa in sequencing data (Robinson and Smyth 2007; Anders and Huber 2010). Also, the zero-inflated component raises the need for an EM-algorithm which is computationally demanding. The *gomms* package does not exploit the taxon scores for making biplots, and no diagnostic plots nor a constrained counterpart are available.

2.2.4.3 Row-column interaction models

Our unconstrained RC(M) method is a special case of the row-column interaction models (RCIM) of (Yee and Hadi 2014), which generates the model RC(M) from Goodman (1979). We have written our own, faster implementation and improved dispersion estimation for the negative binomial error model.

2.2.4.4 Latent variable models

The latent variable model by Hui et al. (2015) can also be regarded as a modified version of a row-column interaction model, but there the row and column scores are not treated symmetrically, and also no constrained version is available. The sample scores are considered to be random effects, which greatly complicates their estimation.

The clustering model by Pledger and Arnold (2014) follows a similar reasoning, but does not assign unique scores to all taxa and samples.

2.2.5 Relationship between constrained RC(M) and existing methods

2.2.5.1 Constrained correspondence analysis

The solution of constrained correspondence analysis (CCA) corresponds to that of quadratic response curves with tolerances equal for all taxa and augmented with a Poisson distribution (Zhu et al. 2005). Whereas the constrained RC(M) model models the departure from independence in a multiplicative way, correspondence analysis captures departure from independence in an additive way by modelling the residuals. In CCA the environmental gradients are not made orthogonal as in the RC(M)-model, but the sample scores are (ter Braak 1986).

2.2.5.2 Environmental gradient estimation

Most existing methods to estimate response functions and environmental gradients use linear, quadratic and non-parametric response function as in our RC(M) methods (Zhu et al. 2005; Yee and Hadi 2014). They fail however to account explicitly for differences in sequencing depth and taxon abundance, as our method does by estimating the independence model. Our RC(M) method has borrowed the log-likelihood ratio approach of estimating the environmental gradient based on niche separation from (Zhu et al. 2005).

2.3 Plotting the RC(M) ordination

2.3.1 Unconstrained RC(M)

To plot the unconstrained sample ordination, e.g. in the first two dimensions, plot $\psi_1 r_{1i}$ vs $\psi_2 r_{2i}$, preferably as dots. All weight of the importance parameters ψ_m is allotted to the samples, which means that the distances between sample points can be interpreted as optimal representations of between-sample distances in lower dimension: more weight is added to differences in sample scores in important dimensions.

To show the role of the taxa in the ordination, add taxon scores s_{1j} vs s_{2j} as arrows to make a biplot. This assures that the orthogonal projection of the vector $(\psi_1 r_{1i}, \psi_2 r_{2i})$ on (s_{1j}, s_{2j}) equals $(s_{1j}, s_{2j})^t(\psi_1 r_{1i}, \psi_2 r_{2i}) = \sum_{m=1}^2 \psi_m r_{im} s_{jm}$. This inner product is thus proportional to the departure from independence in the first two dimensions combined, for taxon j in sample i: $\psi_1 r_{1i} s_{1j} + \psi_2 r_{2i} s_{2j}$. The larger the entries of the species and sample scores (the scaling between these two sets is arbitrary, we usually choose them in the same order of magnitude) and the smaller the angle between the vectors, the larger the departure of this taxon in this sample.

Distances between taxon arrows are meaningless in this representation. To avoid misleading plots it is of primary importance to use the same scale on all axes, no matter how rectangular and inconveniently shaped this renders the plot.

2.3.2 Constrained RC(M)

2.3.2.1 Linear response functions

For a constrained ordination with linear response functions, plot the sample scores $(\psi_1 \boldsymbol{\alpha}_1^t \mathbf{c}_i, \psi_2 \boldsymbol{\alpha}_2^t \mathbf{c}_i)$, preferably as dots. Again this ordination optimally represents distances between samples in low dimension, but now only with respect to the variability that can be explained by the environmental variables.

Taxon arrows can be added to make a biplot. The taxon arrows have their origin in $(-\frac{\beta_{0j1}}{\beta_{1j1}}, -\frac{\beta_{0j2}}{\beta_{1j2}})$. This point represents the combination of values of the environmental scores in the first two dimensions were a sample would have no expected departure from homogeneity for this taxon j. The arrow then extends in the direction of $(\beta_{1j1}, \beta_{1j2})$ with length proportional to $\sqrt{\beta_{1j1}^2 + \beta_{1j2}^2}$. The orthogonal projection of this taxon vector onto the sample scores (which depart from the origin), is then equal to $(\beta_{1j1}, \beta_{1j2})^t(\psi_1 \boldsymbol{\alpha}_1^t \mathbf{c}_i, \psi_2 \boldsymbol{\alpha}_2^t \mathbf{c}_i) =$

$\sum_{m=1}^{2} \psi_m \beta_{ijm} \boldsymbol{\alpha}_m^t \mathbf{c}_i$, i.e. the departure from uniformity of taxon j that is due to the environmental score from sample i.

In order to make a triplot, labels for the environmental variables are then added according to the loadings of $\boldsymbol{\alpha}_m$. The projection of $\boldsymbol{\alpha}_y$ (the component of $\boldsymbol{\alpha}$ belonging to variable y) onto the taxon arrows then reflects the sensitivity of the expected abundance of taxon j to changes in variable y. For the categorical variables all levels are shown on the plot, there are no hidden reference levels. The continuous variables represent changes of the magnitude of one standard deviation. Comparison of the magnitude of the loadings of continuous and categorical variables is inherently difficult.

There is no interpretation available for the relative position of sample and variable vectors. This is because the environmental gradient $\boldsymbol{\alpha}_m$ projects the environmental variables of a sample i, $\mathbf{c}_i$, onto a single scalar h_{im}, the environmental score. Many combinations of variables $\mathbf{c}_i$ can lead to the same environmental score. Again, distances between taxon arrows are meaningless in this representation.

2.3.2.2 Quadratic response function

For a quadratic response function the samples are ordered as for the linear one.

The taxa are plotted as dots at the locations $(-\frac{\beta_{2j1}}{2\beta_{3j1}}, -\frac{\beta_{2j2}}{2\beta_{3j2}})$ of maximal departure from independence. The convexity $\beta_{3jm} < 0$ or concavity $\beta_{3jm} > 0$ in each dimension can be shown e.g. by a colour code. Note that cases like $\beta_{3j1} < 0 < \beta_{3j2}$ can occur, which greatly complicates the interpretation. Further, ellipses can be drawn around the taxon points to indicate the steepness of the response functions. We choose to draw ellipses connecting the values of the environmental score at which the response functions are at 95% of their peaks.

The environmental variables can be added as in the linear case to show how they contribute to the environmental gradient.

2.3.2.3 Non-parametric response functions

For non-parametric response functions, the species cannot be easily plotted in 2D, and the samples plot would be meaningless due to the irregular shape of the response functions. The only 2D plot that can be made is the variables plot. However, as before distance between variables are not meaningful, and the gradients in both dimensions should be interpreted separately.

The most important plot for non-parametric response functions is the one-dimensional triplot. This plot shows the shape of the response function as a function of the environmental score in one dimension. The environmental gradient of this dimension can be added as a reference to show which variables constitute the gradient. Also sample scores can be added to this one dimensional triplot. The sacrifice of one dimension is needed as the y-axis is used to depict the irregular shape of the response function.

2.4 Assessing the model quality

Even though it is only an explorative visualization and conclusions may still be valid in the face of slight violation of its assumptions, we need tools to evaluate the goodness of fit of the RC(M) model and the validity of its assumptions.

2.4.1 Parsimony

An unconstrained RC(M) model of dimension m on a n×p data matrix requires estimation of p (abundances) + n (library sizes) + p (dispersions) + mp (column scores) + mn (row scores) + m (importance parameters) = (m+2)p + (m+1)n + m parameters out of np entries. 4m + m(m-1) restrictions have been imposed, so the final model is still very parsimonious for realistic sizes of n and p (hundreds).

A constrained RC(M) model with linear response functions of dimension m on a n×p data matrix and with a n×d covariate matrix requires estimation of p (abundances) + n (library sizes) + p (dispersions) + 2mp (response function parameters) + md (environmental gradient loadings) + m (importance parameters) = (2m+2)p + m(d+1) + n parameters out of np entries. 3m + m(m-1) restrictions have been imposed.

2.4.2 Importance of the dimension

A very natural question is to know how much more important the lower dimensions are in explaining the present variability then the higher dimensions. Also we would want a measure of how much of the variability has been explained in lower dimensions. In principal components analysis (PCA) there is the concept of "percentage variance explained", in correspondence analysis (CA) the total inertia is known and thus also the percentage of variance captured by the lower dimensions. Still the value of these expressions is questionable, since they only yield a fraction of *total* variability. However, part of the total variability is noise, and one does not know which percentage of the *signal* the higher dimensions explain. In Principal Coordinates Analysis (PCoA) measures of importance of the dimension are also given as the percentage of variance explained. This can be misleading however, as this refers to the variance *of the distance* matrix explained. In the calculation of the distance matrix, already some of the variability is discarded. As a result these percentages should be interpreted with caution, and not as a function of *total variability.*

Since for the RC(M) model for computational reasons only a couple of dimensions are fitted, it is harder to come up with a measure of total variability.

2.4.2.1 Importance parameters

The best measure of differences in importance between the dimensions are the importance parameters ψ_m. Since all other parameters in both the unconstrained and constrained variables are normalized, these are the only ones that can grow in magnitude to give more weight to the departures of independence in their dimension. This is very similar to the eigenvalues in PCA or the singular values in correspondence analysis, whose size is proportional to the importance of the corresponding dimension. In both the unconstrained and the constrained case it may occur that the magnitude of the ψ_m's is not always monotonically decreasing with the dimensions. However, for skewed distributions as the negative binomial this need not be surprising: the strongest improvement in likelihood is not always achieved by the greatest change in the mean, especially in the presence of nuisance parameters. As long as the ordination axis are properly scaled this does not invalidate the interpretation of the ordination plots.

The plotting procedure described above will allot all weight of the importance parameter to the samples, thus automatically weighting for the importance of the dimensions in the sample ordination. As such the ψ's will not directly contribute to the interpretation of the plot.

2.4.2.2 Log-likelihoods

Another way to quantify the importance of the dimensions is to compare their differences in log-likelihoods of a model of dimension m (ll_m) with respect to the saturated model. These differences are also known as half the "deviances". The log-likelihood saturated model (ll_{sat}) is calculated using the Poisson density, setting mean and variance equal to the observed counts. These differences in log-likelihood are then normalized with respect to the difference in log likelihood between the independence model ($ll_{independence}$) and the saturated model. The terms obtained for dimension m is then:

$$\frac{ll_m - ll^*}{ll_{sat} - ll_{independence}},$$

with ll^* the log-likelihood of the lower dimension, which can be the independence model, the model after filtering on confounders or simply the lower dimension (m-1) of the RC(M) model.

This approach has the advantage of also providing a measure of importance for the confounders. Also it uses the saturated model as a reference and thus provides a fraction of “total variability”.

Disadvantages are the difficulty in interpreting log-likelihoods, and the fact that in some corner case the log-likelihood drops with higher dimensions. This is because the estimation of the dispersions and environmental gradients is not full maximum likelihood.

2.4.2.3 Inertia

As with correspondence analysis, the fraction of total inertia explained by the different dimensions can be plotted on the axes. The inertia is defined as the sum of squared Pearson residuals, or

$$\sum_{i=1}^{n}\sum_{j=1}^{p}\frac{(x_{ij}-e_{ijm})^2}{e_{ijm}}$$

with x_{ij} the observed count and e_{ijm} the expected count under the model with m dimensions. The inertia has the advantage that also the variance explained by the filtering on confounders step can be plotted, and that there is a measure of residual variance.

On the other hand, as we argue in the manuscript, the inertia is not a good measure of variability for overdispersed data, as it implicitly assumes the mean to equal the variance. Hence this criterion of dimension importance should be interpreted with caution.

2.4.3 Detecting lack of fit

In our 2D or 3D representation, some samples and taxa may be very well represented, but others not. This may be because of a lack of fit of the negative binomial distribution, or because its departure from independence cannot be represented in lower dimension. Anyhow, we provide tools to detect lack of fit.

2.4.3.1 Deviance residuals

The deviance residuals d_{ij} of the negative binomial distribution are defined as (Zwilling 2013):

$$d_{ij}=\begin{cases} sgn(x_{ij}-\mu_{ij})\sqrt{2\Big(x_{ij}ln(\frac{x_{ij}}{\mu_{ij}})-(x_{ij}+\frac{1}{\phi_j})ln(\frac{1+x_{ij}\phi_j}{1+\mu_{ij}\phi_j})\Big)} \text{ if } x_{ij}>0\\ sgn(x_{ij}-\mu_{ij})\sqrt{\frac{2}{\phi_j}ln(1+\phi_j\mu_{ij})} \text{ if } x_{ij}=0 \end{cases}$$

Their sum of squares equals the total deviance per sample or taxon. We can visually represent the mean deviance per sample or taxon by colour codes. In the constrained case with parametric response functions, plotting the deviance residuals as a function of the environmental gradient can reveal patterns and thus lack of fit to the linearity assumption.

We can do this for the taxa that respond strongest to the environmental gradient, and make residual plots with deviance or Pearson residuals. An alternative is to try to detect systematic trends through series of positive or negative residuals using Ward and Wolfowitz’ runs test, and plot the taxa with the largest test statistic.

2.4.4 Identifying influential observations

Since we have explicitly expressed all score functions, we can easily identify influential observations using *influence functions* (Hampel et al. 2011). They represent the influence a certain observation has on a parameter,

keeping the other sorts of parameters fixed. Because of the iterative algorithm this latter assumption is incorrect, but the influence functions might still harbour interesting information.

For maximum likelihood estimation the influence function $\chi(\gamma|f, \mathbf{x})$ of a parameter γ for a distribution f and data $\mathbf{x}$ is defined as:

$$\chi(\gamma|f, \mathbf{x}) = -\mathbf{S}_f(\gamma|\mathbf{x}) E\big(\mathbf{I}(\gamma|f)\big)^{-1}$$

with $\mathbf{S}_f(\gamma|\mathbf{x})$ the score function and $E\big(\mathbf{I}(\gamma|\mathbf{x})\big)$ the expected Fisher information matrix.

For the unconstrained case in a scenario without outliers the influence functions may not yield very surprising results on the level of the plot, observations mainly have influence on their own row and column scores. Coupling through the constraints is rather weak. It may however help to identify outlying abundances in case of outlying row- or column scores.

For the constrained case it may be enlightening to see which samples (and taxa) affect the estimation of the environmental gradient most.

3 Simulation study

In this document we present three ways of testing an ordination method:

1) Parametric simulation with the known underlying groups
2) Non-parametric simulation with SimSeq
3) Applying the method to real datasets with biological signal allegedly known

Parametric simulation is convenient since the underlying truth is known, but its parametric assumptions may be violated. SimSeq provides non-parametric data resampling, and is thus a reasonably neutral tool. Examples of real datasets can be found above. Code for all simulations can be found in the Supplementary File RCMcode.R.

Computations were run on a Dell laptop, on two servers with 12 respectively 30 cores and on the high performance computing facilities of VSC (the Flemish Supercomputer Center). All analyses were run with the R programming language versions 3.5.1, 3.4.3 and 3.3.1 (R Core Team 2015).

3.1 Parametric simulations

All simulations were performed with n=60 samples and p=1000 taxa.

Parametric simulations under sample homogeneity (i.e. without signal) were set up by simulating counts from the negative binomial distribution with equal mean taxa composition in all samples. In a one scenario (NB0 (lib)), prior to data generation, the samples were divided into 4 equally sized groups. The sampled library sizes were multiplied by 0.2, 1, 5 and 10 in each of these groups, respectively. In a second scenario (NB0 (disp)), the sampled taxon-wise dispersions were multiplied by 0.2, 1, 2 and 5 in the 4 groups prior to data generation.

For parametric simulations with differences in mean taxa composition (with biological signal), counts were generated for 4 equally sized groups of samples with different taxa compositions. In a first setting (NB), initially one taxa composition was sampled for all the groups. This composition was then altered for every group separately by multiplying a random sample of 10% of the taxa abundances by a fold change of 5 to make them differentially abundant (DA). The second setting (NB (cor)) was identical to the first, except that counts were generated with between-taxon correlations, as estimated by SpiecEasi (Kurtz et al. 2015) on the mid vagina, stool and tongue dorsum datasets of the HMP and on the AGP data. A correlation network was sampled for every Monte Carlo instance. The third scenario (NB (phy)) was also similar to the first, only now a random phylogenetic tree was created for every dataset. Next, the tree was divided into 20 clusters of related taxa, and differential abundance was introduced in one of the clusters with a fold change of 5. This assures that the DA taxa are phylogenetically related, similar to the second approach in Chen et al. 2012. A fourth scenario (ZINB) uses the same strategy for the introduction of differential abundance as the first, but uses zero-inflated negative binomial distributions. The DA is introduced only in the count part of the distribution. A fifth scenario (DM) uses the Dirichlet multinomial distribution, for which DA is introduced as for the first scenario. In a sixth setting (NB (unrel)), for every group taxa compositions were sampled independently from the pool of estimated mean relative abundances. In a seventh scenario (NB (lib)), differential abundance was introduced as in the first scenario, but in this case we will also make the library sizes different in four groups. However, these library-size groups will not coincide with the composition groups. On the contrary, each composition group will have equal number of samples from the same library size group. The initial library sizes are all sampled from the same pool of library sizes. The first group has unmodified library sizes, the second group library sizes multiplied by 1.5, the third by 2 and the fourth by 3. In a eighth scenario (NB (disp)), the taxon-wise dispersions are modified in four groups, but these dispersion groups will not coincide with the composition groups. On the contrary, each composition group will have equal number of samples from the same dispersion group. For this aim, the first group has unmodified taxon dispersions, the second group dispersions multiplied by 5, the third by 2 and the fourth by 0.25. In a tenth scenario, data were generated with the NB distribution, but without any biological signal. The tenth and

eleventh scenarios (NB (lib2) and NB (disp2)) also modify library sizes and dispersion, but in this case the DA and library size/dispersion groups coincide.

3.1.1 Summary table of the simulation scenarios

Simulation.scenario	Template	Distribution	Signal	Remarks
NB0	AGP + HMP	NB	No	
NB0 (lib)	AGP + HMP	NB	No	Groups differ in library sizes
NB0 (disp)	AGP + HMP	NB	No	Groups differ in dispersions
NB	AGP + HMP	NB	Yes	
NB (cor)	AGP + HMP	NB	Yes	Correlated taxa
NB (phy)	AGP + HMP	NB	Yes	Phylogenetically related taxa were made DA
DM	AGP + HMP	DM	Yes	
ZINB	AGP + HMP	Zero-inflated NB	Yes	
NB (unrel)	AGP + HMP	NB	Yes	Taxon compositions were sampled independently
NB (lib)	AGP + HMP	NB	Yes	Groups differ in library sizes, but orthogonally to DA
NB (disp)	AGP + HMP	NB	Yes	Groups differ in dispersions, but orthogonally to DA
NB (lib2)	AGP + HMP	NB	Yes	Groups differ in library sizes, same groups as DA
NB (disp2)	AGP + HMP	NB	Yes	Groups differ in dispersions, same groups as DA
Props (cycle)	Props	Real data	Yes	Non-parametric simulation
Props (phase)	Props	Real data	Yes	Non-parametric simulation
Kostic (country)	Kostic	Real data	Yes	Non-parametric simulation
Kostic (diagnosis)	Kostic	Real data	Yes	Non-parametric simulation
Zeller (diagnosis)	Zeller	Real data	Yes	Non-parametric simulation
Turnbaugh (diet)	Turnbaugh	Real data	Yes	Non-parametric simulation

Table S1: Summary table of simulation scenarios. DA: differentially abundant, NB: negative binomial, DM: Dirichlet multinomial

3.1.2 Overview of the parametric simulation workflow

a) Assume a parametric distribution and estimate corresponding parameters

taxon 1	...	taxon p
ρ_1	...	ρ_p
$\boldsymbol{\theta}_1$	...	$\boldsymbol{\theta}_p$

The ρ_j parameters reflect the mean relative abundance of each taxon j, whereby $\sum_{j=1}^{p} \rho_j = 1$. $\boldsymbol{\theta}_p$ contains all other parameters estimated for taxon j.

- Scenarios 1-3 and 6-8: Negative binomial distribution
- Scenario 4 (ZINB): Zero-inflated negative binomial distribution
- Scenario 5 (DM): Dirichlet-multinomial distribution

b) Obtain different taxon compositions for every of the 4 groups

- Scenario 6 (NB (unrel)): Sample a taxon composition from the pool of parameter estimates for every group separately

Groups	taxon 1	...	taxon p
group 1	ρ_{11}	...	ρ_{1p}
group 2	ρ_{21}	...	ρ_{2p}
group 3	ρ_{31}	...	ρ_{11}
group 4	ρ_{41}	...	ρ_{41}

- Scenarios 1-5 and 7-8: Sample one taxon composition, and introduce differential abundance by multiplying a random sample of 5% of the taxa by a fold change of 5

Groups	taxon 1	...	taxon a	taxon b	...	taxon c	taxon d	...	taxon e	taxon f	...	taxon p
group 1	ρ_{11}	$\dots$	ρ_{1a}	ρ_{1b}	$\dots$	ρ_{1c}	ρ_{1d}	$\dots$	ρ_{1e}	ρ_{1f}	$\dots$	ρ_{1p}
group 2	ρ_{21}	$\dots$	ρ_{2a}	ρ_{2b}	$\dots$	ρ_{2c}	ρ_{2d}	$\dots$	ρ_{2e}	ρ_{2f}	$\dots$	ρ_{2p}
group 3	ρ_{31}	$\dots$	ρ_{3a}	ρ_{3b}	$\dots$	ρ_{3c}	ρ_{3d}	$\dots$	ρ_{3e}	ρ_{3f}	$\dots$	ρ_{3p}
group 4	ρ_{41}	$\dots$	ρ_{41}	ρ_{4b}	$\dots$	ρ_{4c}	ρ_{4d}	$\dots$	ρ_{4e}	ρ_{4f}	$\dots$	ρ_{4p}

- Scenario 4 (NB (phy)): Differential abundance is introduced in phylogenetically related taxa

c) Generate random data according to the chosen distribution

Scenario 2 (NB (cor)): Use an estimated taxon correlation structure

Groups	taxon 1	...	taxon p
group 1	x_{11}	$\dots$	x_{1p}
$\vdots$	$\vdots$	$\ddots$	$\vdots$
group 1	$x_{n_1 1}$	$\dots$	$x_{n_1 p}$
group 2	$x_{(n_1+1)1}$	$\dots$	$x_{(n_1+1)p}$
$\vdots$	$\vdots$	$\ddots$	$\vdots$
group 2	$x_{(n_1+n_2)1}$	$\dots$	$x_{(n_1+n_2)p}$
group 3	$x_{(n_1+n_2+1)1}$	$\dots$	$x_{(n_1+n_2+1)p}$
$\vdots$	$\vdots$	$\ddots$	$\vdots$
group 3	$x_{(n_1+n_2+n_3)1}$	$\dots$	$x_{(n_1+n_2+n_3)p}$
group 4	$x_{(n_1+n_2+n_3+1)1}$	$\dots$	$x_{(n_1+n_2+n_3+1)p}$
$\vdots$	$\vdots$	$\ddots$	$\vdots$
group 4	$x_{(n_1+n_2+n_3+n_4)1}$	$\dots$	$x_{(n_1+n_2+n_3+n_4)p}$

d) Apply ordination method and evaluate performance

3.2 Nonparametric simulation

An objective simulation approach would be to use non-parametric resampling from a true dataset, as in *SimSeq* (Benidt and Nettleton 2015). For this we need microbiome datasets with covariates known to be related to bacterial abundance, preferably with more than two groups. The Zeller data is one such dataset, with the cancer variable expected to be related to relative abundance and having three levels (Normal, small adenoma and cancer) (Zeller et al. 2014). For the Turnbaugh dataset we use "Diet", with levels "BK" and "Western" (Turnbaugh et al. 2009). For the Kostic data also cancer diagnosis and country were used (scenarios called KosticDiagnosis and KosticCountry) (Kostic et al. 2014). For the Props data both reactor cycle and phase were used (scenarios called PropsCycle and PropsPhase) (Props et al. 2016). In all settings 100 Monte-Carlo instances were generated.

We generate data as follows

1. Select a covariate and test for differential abundance using Wilcoxon-Mann-Whitney or Kruskal-Wallis test
2. Calculate local false discovery rates (lfdr)
3. Sample non DA taxa with equal weights from all taxa
4. Sample DA taxa from all taxa with weights equal to 1-lfdr
5. Sample counts from non DA taxa from samples with the most frequent covariate level
6. Sample counts from DA taxa also from the samples with other covariate levels, and correct for differences in library sizes. This maintains the same distribution of covariate levels and overall data matrix size as the original dataset.

The *gllvm* method suffered heavily from numeric instability on synthetic datasets generated nonparametrically from the Props2016 and Props2018 datasets. As a result, this method was omitted from the comparison for these datasets.

3.3 Automatic method evaluation

In a simulation we need multiple repetitions to reliably estimate the performance of an ordination method, so we need an automatic evaluation of the quality of the ordination.

3.3.1 Robustness to technical artefacts

The motivating problem to develop the whole method was to find an approach that would not show correlation between the row scores and the library sizes, which is a technical artefact.

Pearson correlations of row scores with library sizes could thus be a criterion to evaluate the quality of the biplot. Equivalently correlations of the taxon scores with average relative abundance and with true logged dispersions can be investigated.

3.3.2 Sample separation

3.3.2.1 Pseudo F-statistic

A pseudo F-statistic for distance matrices has been proposed by (Anderson 2001), and has been applied to simulation studies for ordination methods (Schmidt et al. 2016).

It is calculated as

$$F_{pseudo} = \frac{SS_{overall} - SS_{within}}{SS_{overall}} \frac{n-a}{a-1}$$

with a the number of clusters, $SS_{overall}$ the sum of squared distances of all pairwise combinations of points, and SS_{within} the sum of squared distances of all samples from the same cluster.

3.3.2.2 Silhouette

The silhouette (Rousseeuw 1987) is well established tool to measure sample separation. For each point i, calculate the distance to each other point in the ordination, and average these distances within the cluster. Call $a(i)$ the average distance to its own cluster and $b(i)$ the smallest of the average distances to the other clusters. The the silhouette of observation i $s(i)$ is defined as:

$$s(i) = \frac{b(i) - a(i)}{max\big(a(i), b(i)\big)}$$

The silhouette can take values between +1 for optimal separation and -1 for wrong classification. If a sample i lies close to the centroid of its own cluster but very far from all the others, then it has a high silhouette.

3.3.3 Contribution of taxa to the separation of the clusters

For the methods that do yield taxon scores, we can also verify if the correct taxa contribute to the separation of the clusters. For this purpose we define the following "taxon ratio". This metric is based on the average inner product of the DA taxon scores and the samples scores of samples in which the taxa are known to be differentially abundant. This yields a measure of how much these DA taxa contribute to the separation of the samples. The mean inner product of the non-DA taxon scores with the same sample scores should be small for an ordination method that can discriminate between DA and non-DA taxa. The ratio of the former to the latter mean inner product is the taxon ratio. It is used as a measure of method performance in terms of taxon identification. The taxon ratio captures how well taxa are identified for a single sample cluster.

Call $\mathbf{s}_{l,sig}$ the $p_{l,sig}$×m matrix with taxon scores of the $p_{l,sig}$ taxa that are differentially abundant in the n_l samples of group l. The signal of these taxon scores in the direction of the sample scores $\mathbf{r}_l$ of group l, with $\mathbf{r}_l\boldsymbol{\psi}$ an n_l×m matrix and $\boldsymbol{\psi}$ a diagonal matrix of dimension m with importance parameters on the diagonal, is $\mathbf{1}^t\mathbf{r}_l\boldsymbol{\psi}\mathbf{s}^t_{l,sig}\mathbf{1}$. Hereby $\mathbf{1}$ are unit vectors of the appropriate size that serve to sum all the elements of $\mathbf{r}_l\boldsymbol{\psi}\mathbf{s}^t_{l,sig}$.

Let $\mathbf{s}_{l,noSig}$ be the $p_{l,sig}$×m matrix with scores of the $p_{l,sig}$ non differentially abundant taxa, then the taxon ratio equals

$$\text{Taxon ratio}_l = \frac{p_{l,noSig}}{p_{l,sig}} \frac{\mathbf{1}^t\mathbf{r}_l\boldsymbol{\psi}\mathbf{s}^t_{l,sig}\mathbf{1}}{\mathbf{1}^t\mathbf{r}_l\boldsymbol{\psi}\mathbf{s}^t_{l,noSig}\mathbf{1}}.$$

The taxon ratio was then averaged over all sample clusters.

3.4 Results of simulation study

This section gives an exhaustive overview of all simulation results. In all plots below, ordination methods are coloured according to the underlying paradigm. "Independence" refers to methods that dissect the departure from row-column independence in an additive way, like correspondence analysis. "Distance" refers to methods based on distances between samples, like PCoA. All other methods have particular paradigms and are coloured separately. The RC(M) method had rare cases of non-convergence, for gomms it happened frequently that the fitting process ended with an error or with non-convergence.

3.4.1 No-signal simulations

3.4.1.1 Correlations

Library sizes are considered to be technical artefacts, and thus should not affect the ordination, which is meant to display only biological signal. To verify this we calculate the Pearson correlations of the observed library sizes with every set of sample scores and compare this with the correlation with a random standard normal variable. Further we calculate Pearson correlations of taxon scores to observed taxon abundances and to true logged dispersions. Ideally, these correlations should not be greater than the correlation with the random standard normal variable.

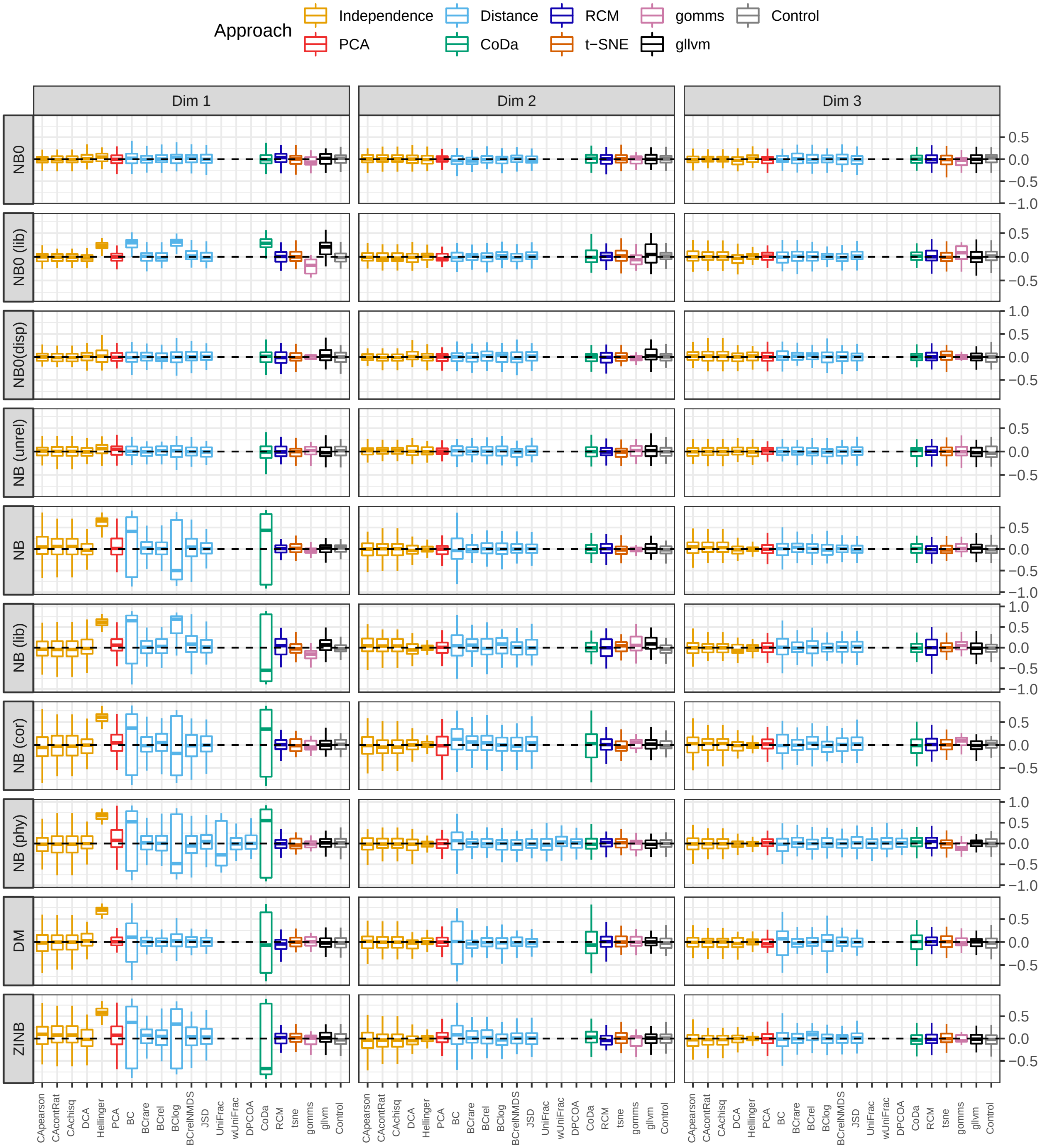

Figure S3: Boxplots with the correlation of sample scores with observed library sizes (y-axis) for different ordination methods (x-axis). Side panels indicate the different parametric simulation scenarios, see Section 3.1 for an explanation of the codes used. Top panels show the dimension of the sample score. Dashed black line indicates zero correlation.

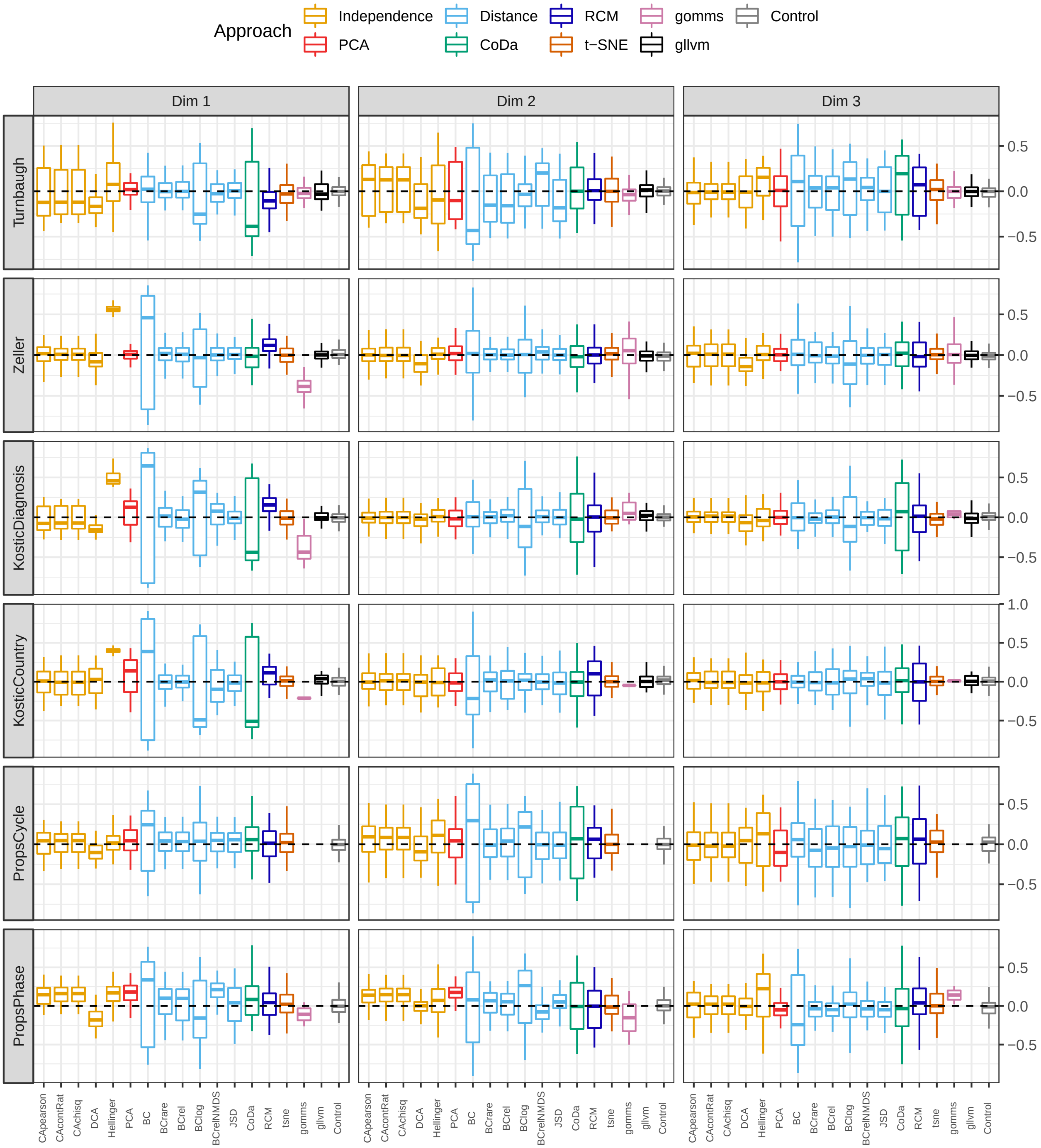

Figure S4: Boxplots with the correlation of sample scores with observed library sizes (y-axis) for different ordination methods (x-axis) in non-parametric simulation. Side panels indicate the different template datasets for non-parametric simulation, see Section 3.2 for further details. Top panels show the dimension of the sample score. Dashed black line indicates zero correlation.

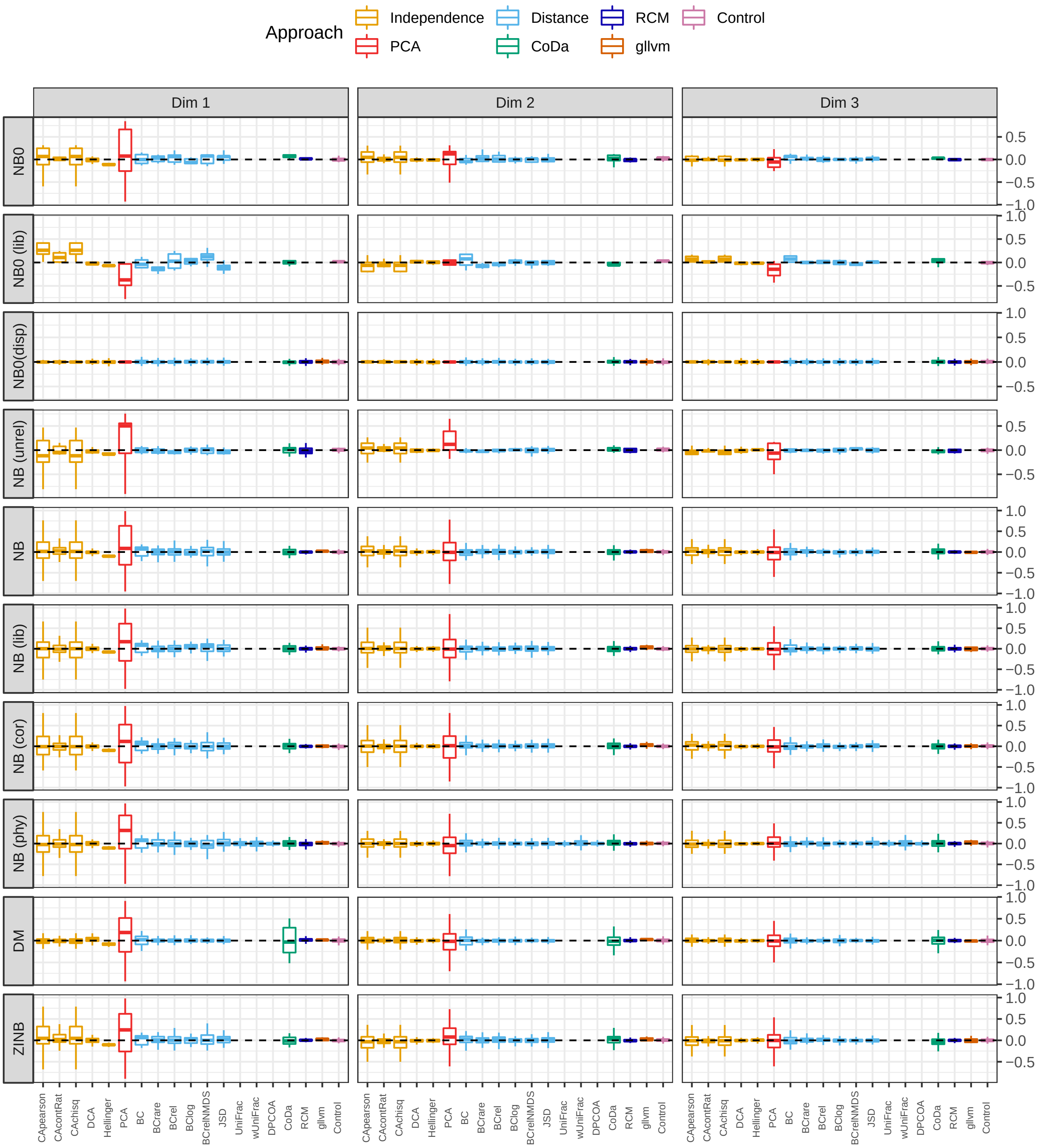

Figure S5: Boxplots with the correlation of taxon scores with observed taxon abundances (y-axis) for different ordination methods (x-axis). Side panels indicate the different parametric simulation scenarios, see Section 3.1 for an explanation of the codes used. Top panels show the dimension of the taxon score. Dashed black line indicates zero correlation.

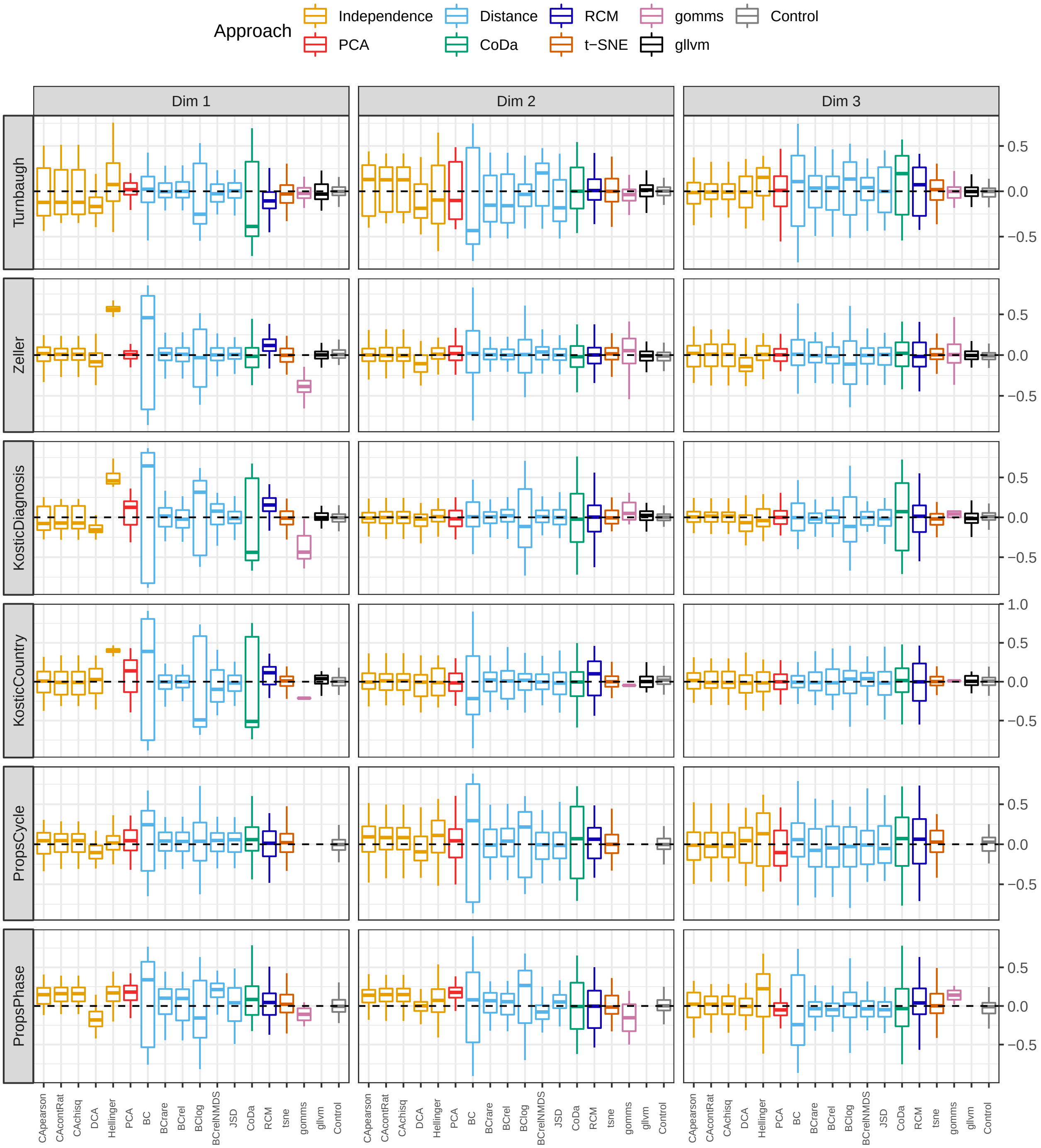

Figure S6: Boxplots with the correlation of taxon scores with observed taxon abundances (y-axis) for different ordination methods (x-axis) in non-parametric simulation. Side panels indicate the different template datasets for non-parametric simulation, see Section 3.2 for further details. Top panels show the dimension of the sample score. Dashed black line indicates zero correlation.

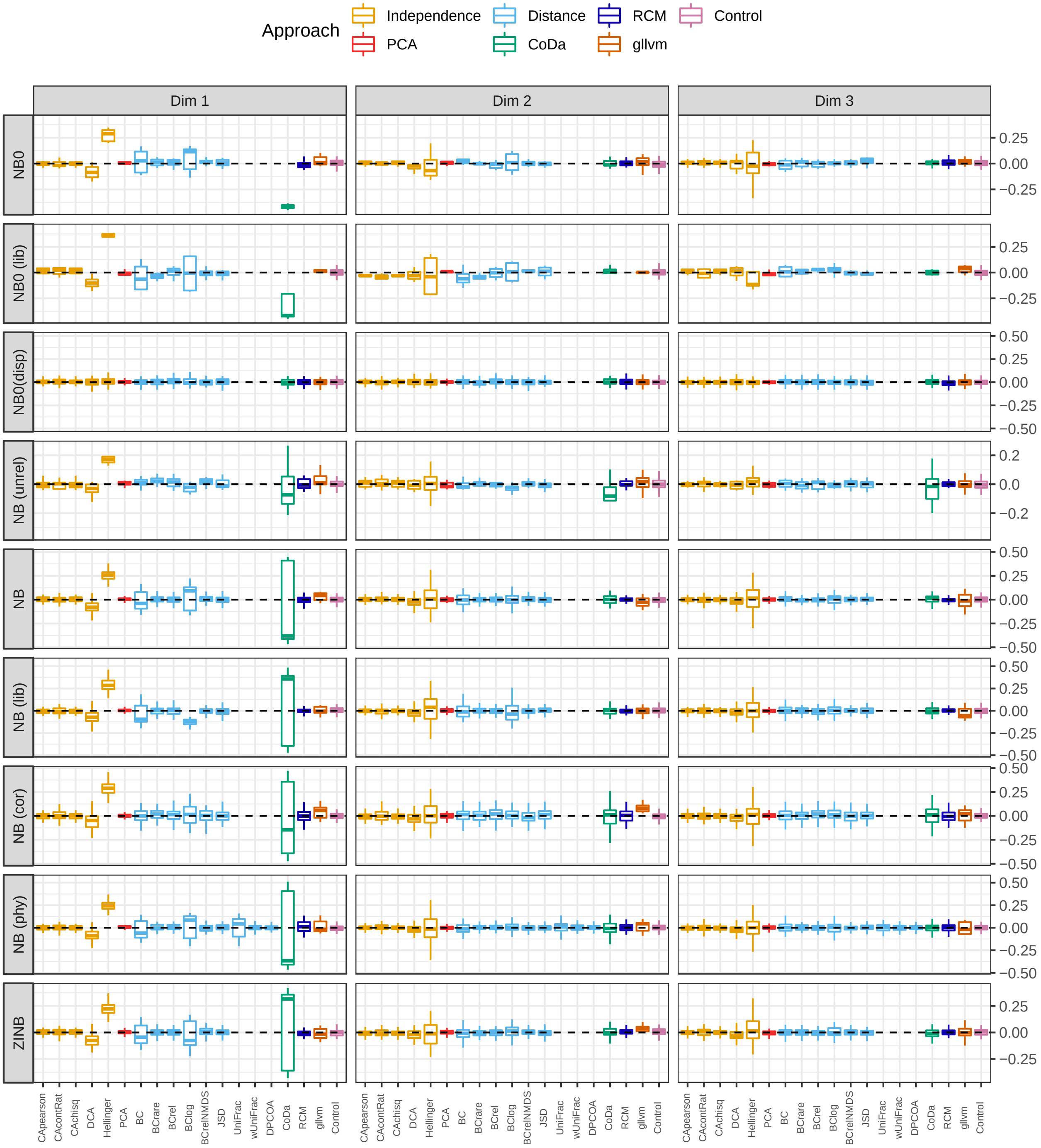

Figure S7: Boxplots with the correlation of taxon scores with true taxon dispersions (y-axis) for different ordination methods (x-axis). Side panels indicate the different parametric simulation scenarios, see Section 3.1 for an explanation of the codes used. Top panels show the dimension of the taxon score. Dashed black line indicates zero correlation.

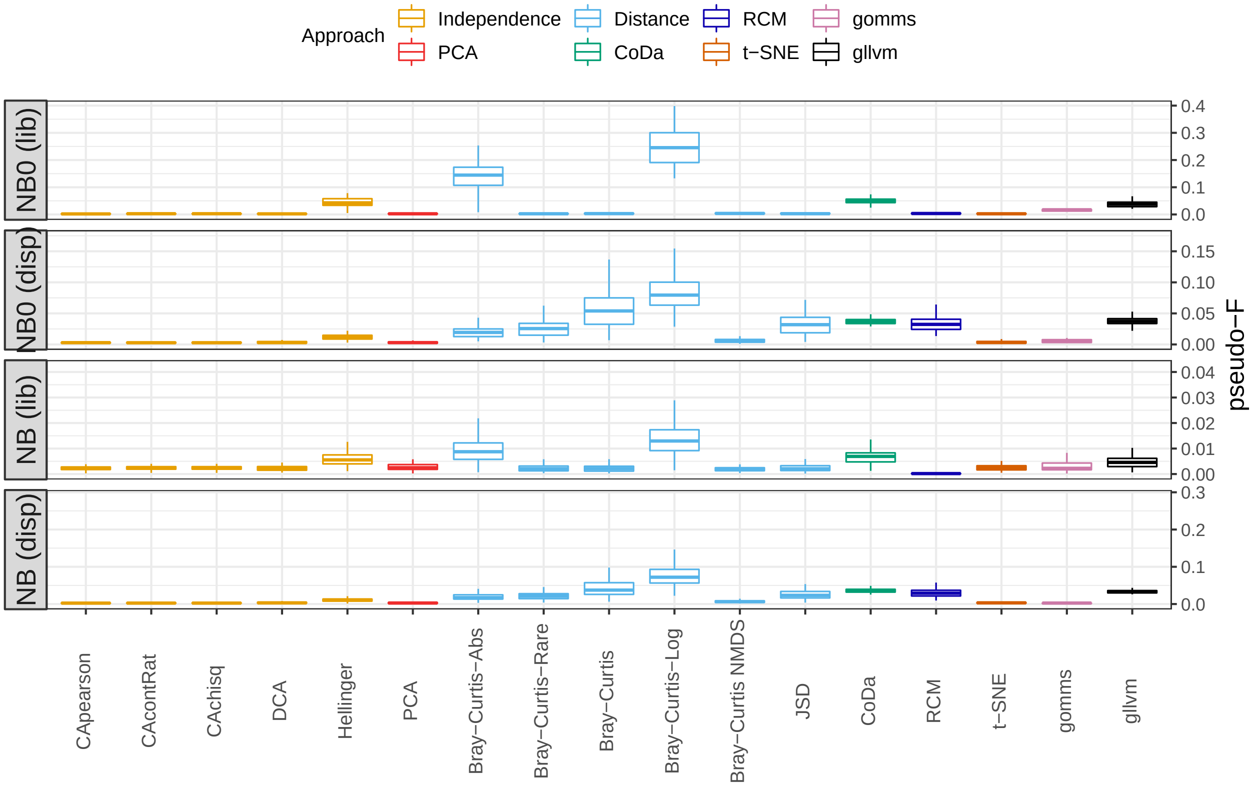

Figure S8: Boxplots of sample clustering measures (y-axis) as a function of ordination methods (x-axis). Side panels indicate the clustering measure used, see Section 3.1 for an explanation of the codes used.

3.4.1.2 Clustering

Differences in sampling techniques or real, biological differences in variability may occur between groups of samples, even when they have the same compositions. Ordinations should be robust to these, so we check if samples with similar variability cluster together.

Also, as a result of the correlation of row scores and library sizes this may lead to clustering of samples with the same composition according to library sizes.

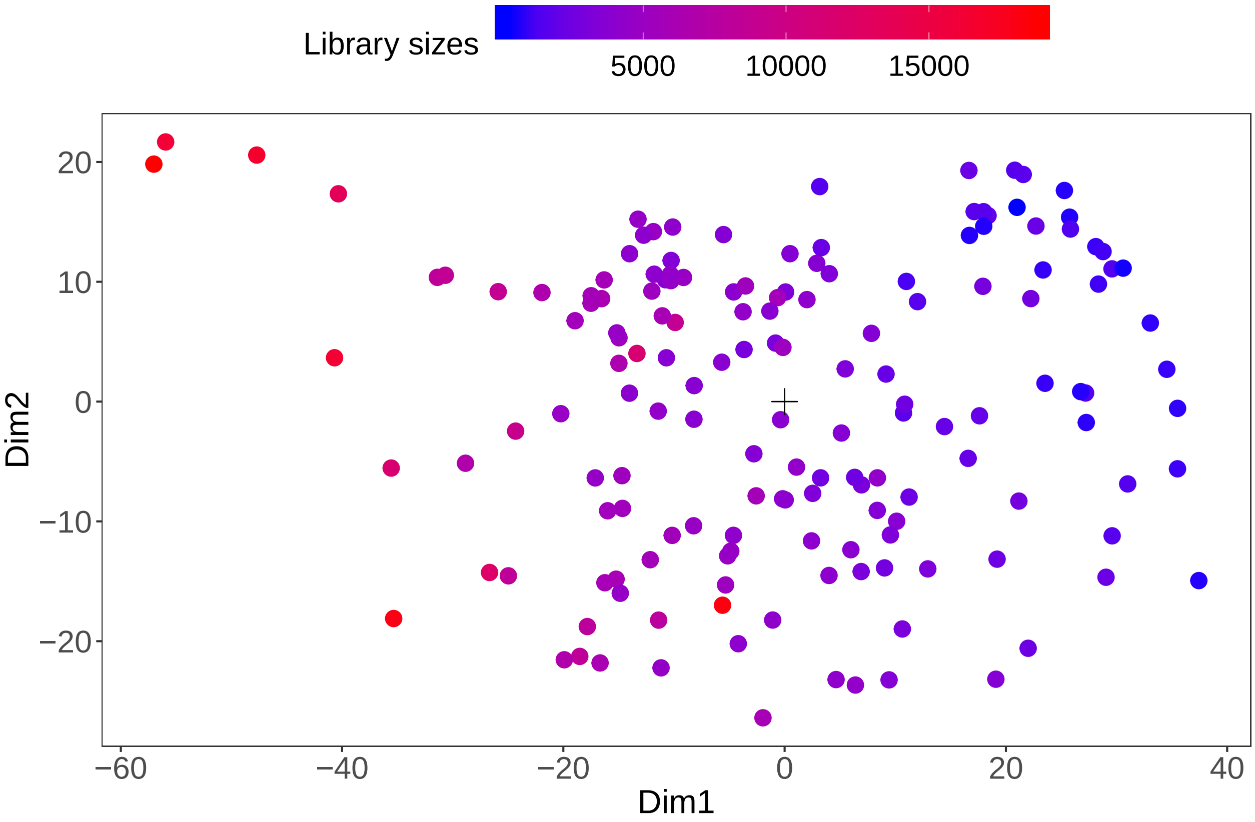

Figure S9: Sample ordination of the Anterior nares dataset from the HMP through CoDa. Samples are coloured by library size.

In absence of biological signal. Most methods do not cluster samples according to library sizes, except for PCoA with Bray-Curtis distances on absolute and logged abundances, the ordination based on the Hellinger distance and CoDa. In presence of biological signal this clustering is much reduced.

All PCoA-based methods investigated (except for NMDS), as well as the CoDa and RC(M) methods, cluster samples according to differences in dispersion, regardless of the presence of biological signal.

We illustrate this effect here for the CoDa method on the Anterior nares dataset from the HMP.

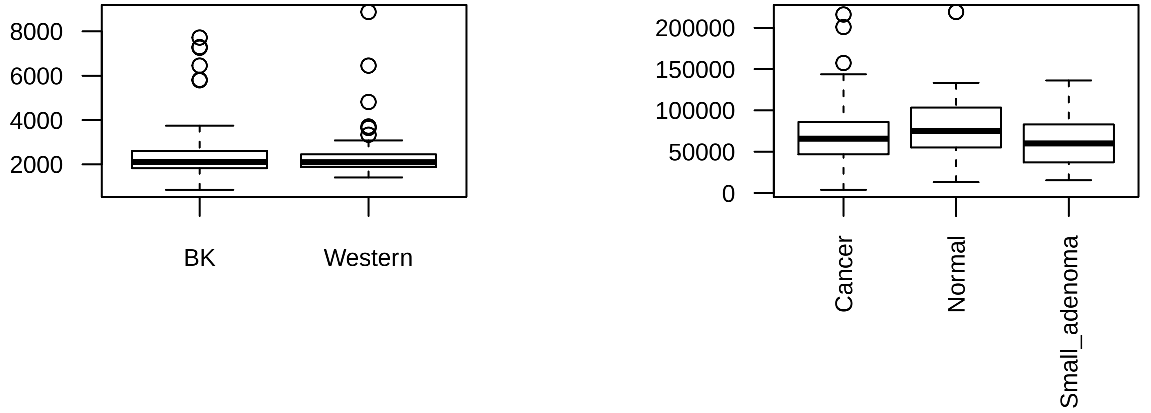

Figure S10: Observed library sizes as a function of diet in the Turnbaugh dataset (left), and as a function of diagnosis in the Zeller dataset (right)

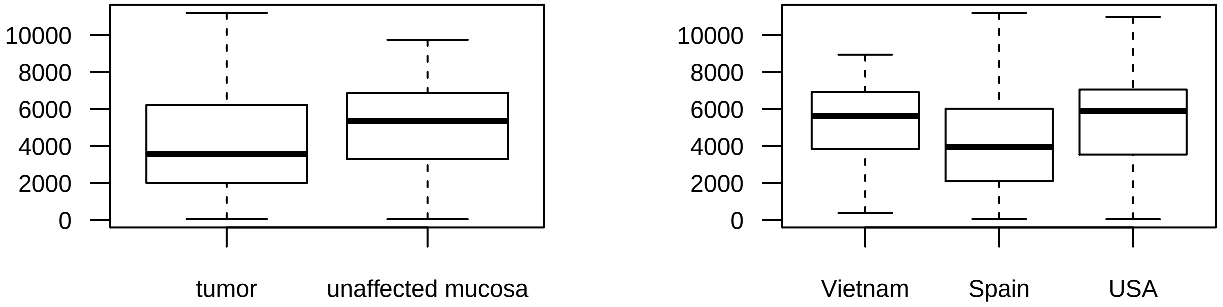

Figure S11: Observed library sizes as a function of diagnosis (left) and country (right) in the Kostic dataset (top)

3.4.1.3 Relation between library sizes and sample covariates

Often library sizes are strongly related to important biological covariates (see boxplots below), and fall into discrete groups. As we have shown that some ordination techniques are sensitive to differences in library sizes, this may lead to very misleading ordination plots.

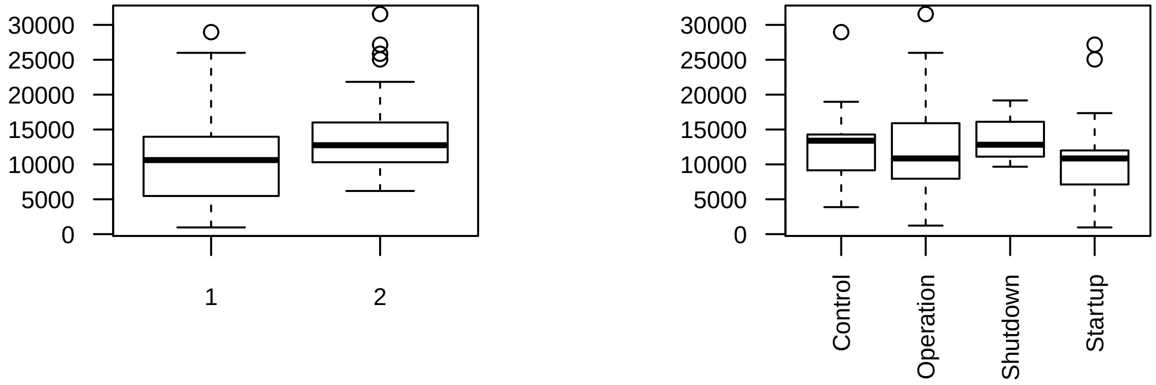

Figure S12: Observed library sizes as a function of reactor cycle (left) and phase (right) in the Props dataset

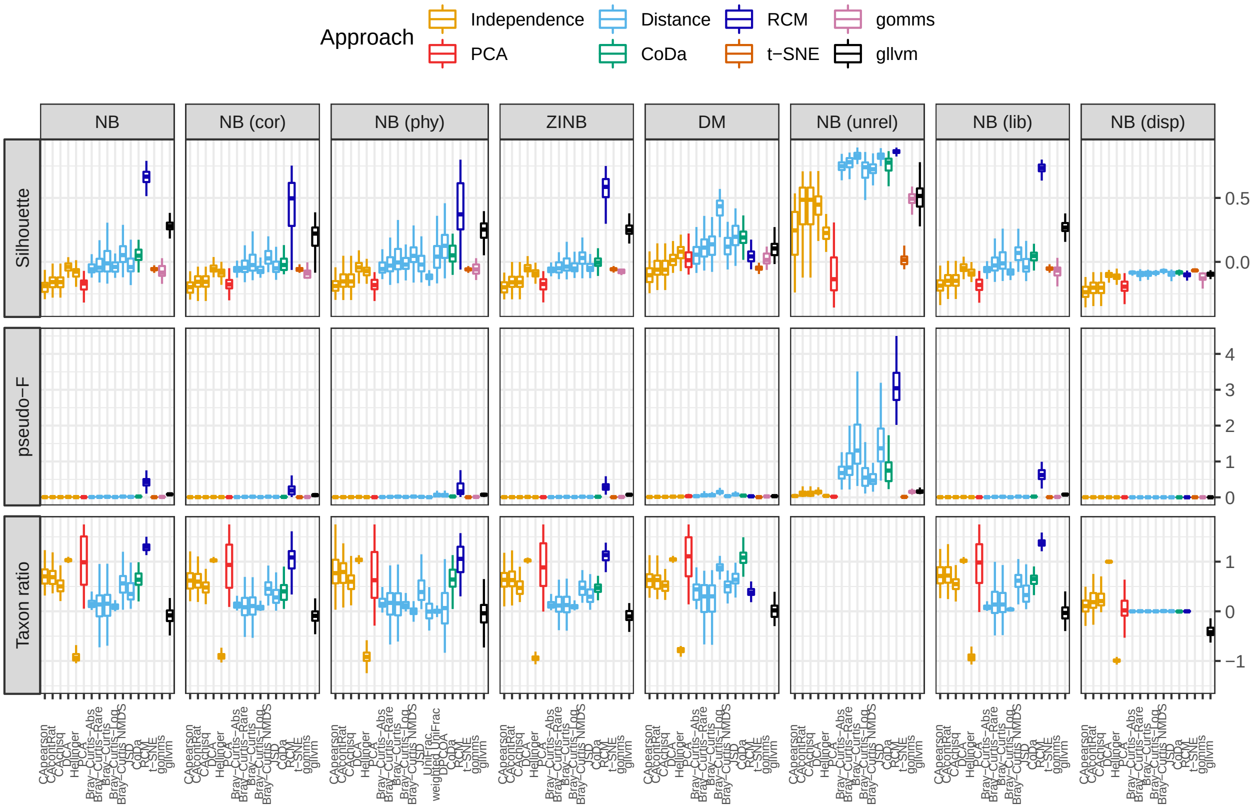

Figure S13: Boxplots of performance measures (y-axis) as a function of the ordination method (x-axis). Top panels indicate the different parametric simulation scenarios, see Section 3.1 for an explanation of the codes used. Left panels indicate the criterion used.

3.4.2 Biological signal simulations

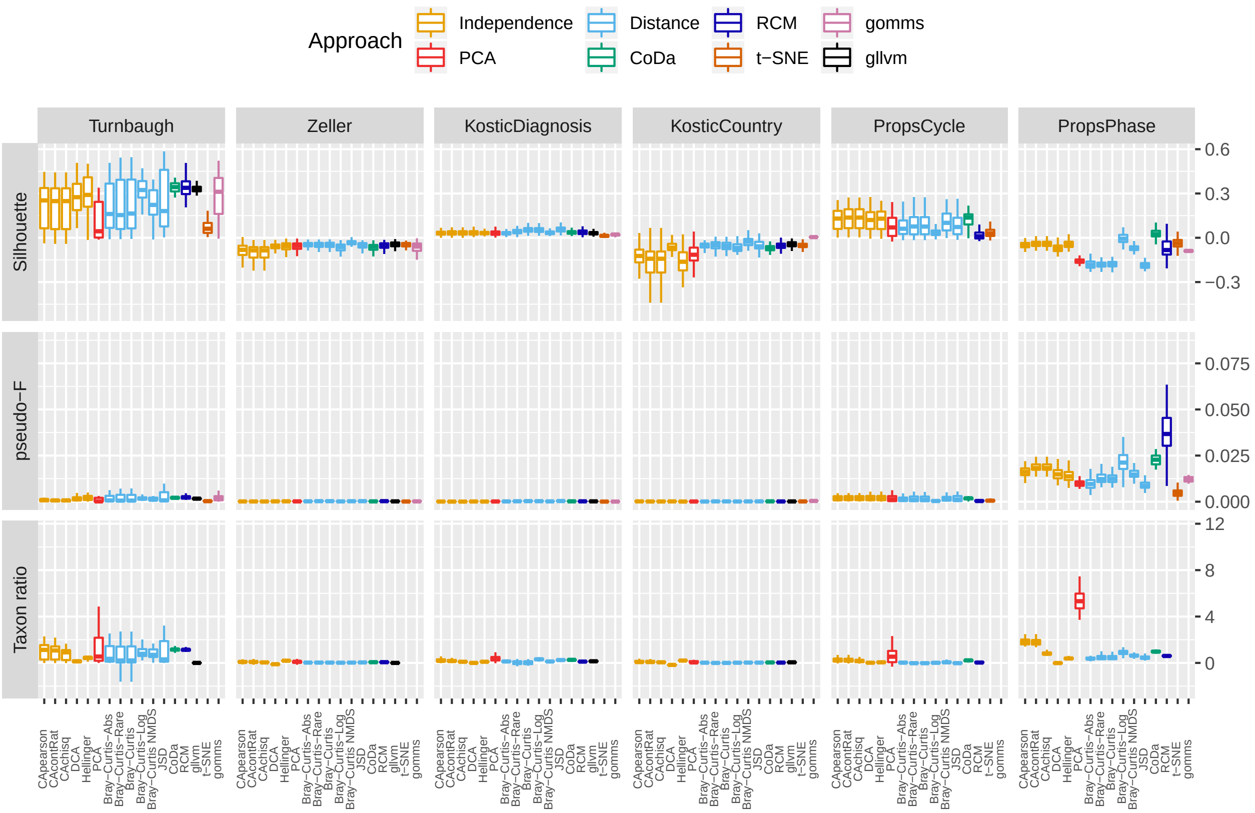

Figure S14: Boxplots of performance measures (y-axis) as a function of the ordination method (x-axis). Top panels indicate the different template datasets for non-parametric simulation (see Section 3.2 for further details), side panels indicate which separation measure is shown.

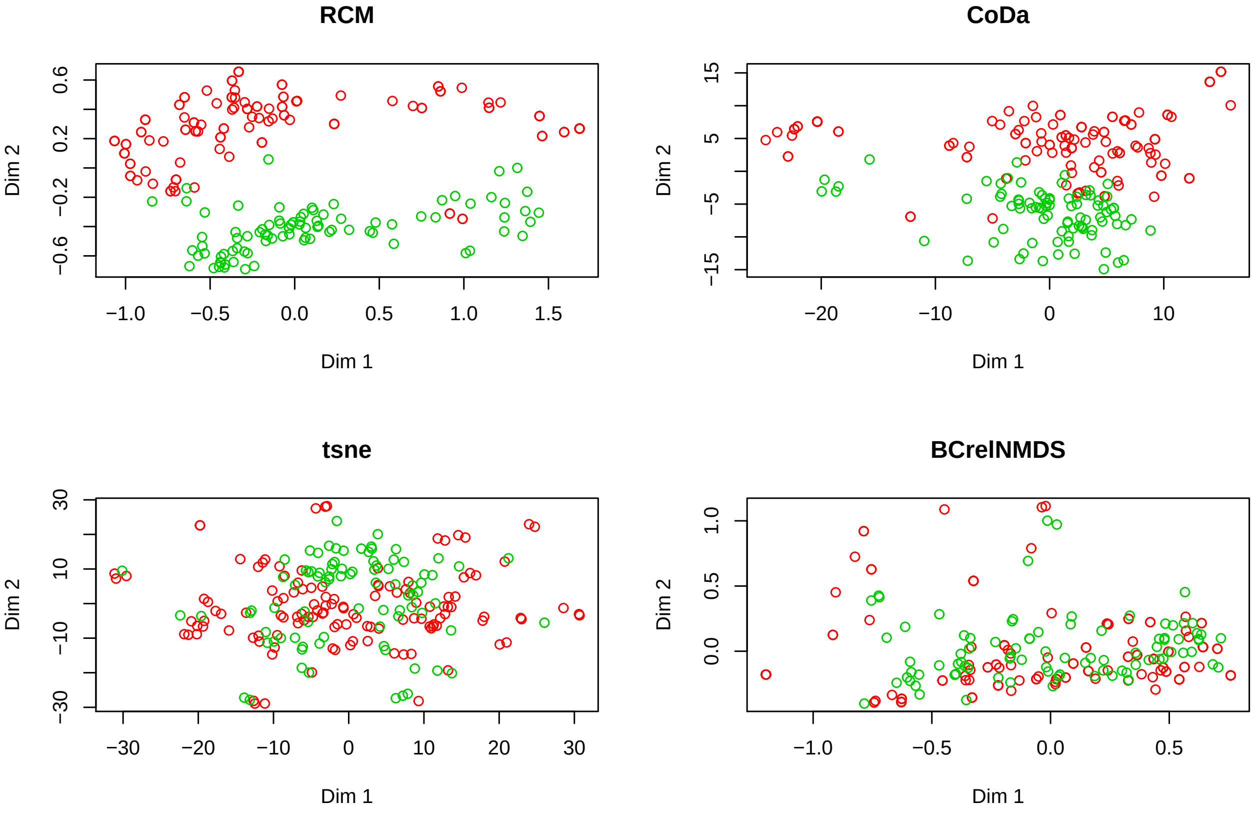

Figure S15: Sample plots of the ordinations by the RC(M), CoDa, t-SNE and NMDS with Bray-Curtis distances on a randomly sampeld SimSeq dataset generated based on the Turnbaugh dataset with diet as grouping variable. Dots are coloured by diet.

3.4.3 Some validation plots

To validate the summary measures used to score sample clustering, we make some example sample plots. Each time we plot the two best, and the two worst performing methods, and see if the result is meaningfully different. We show the ordination graph for each of these methods at their median performance according to the pseudo-F statistic.

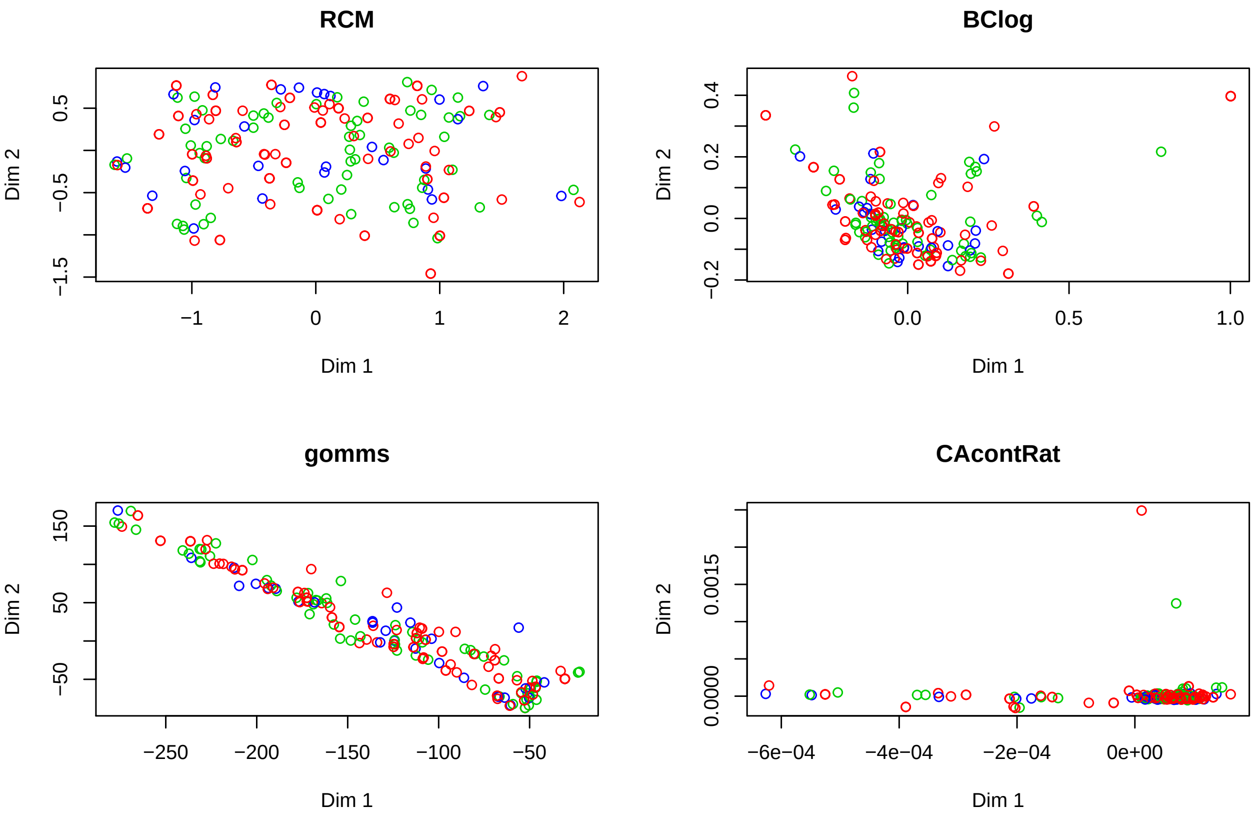

Figure S16: Sample plots of the ordinations by the RC(M), PCoA with Bray-Curtis distances on logged abundances, gomms and correspondence analysis based on the contingency ratio, on a randomly sampeld SimSeq dataset generated based on the Zeller dataset with cancer diagnosis as grouping variable. Dots are coloured by cancer diagnosis.

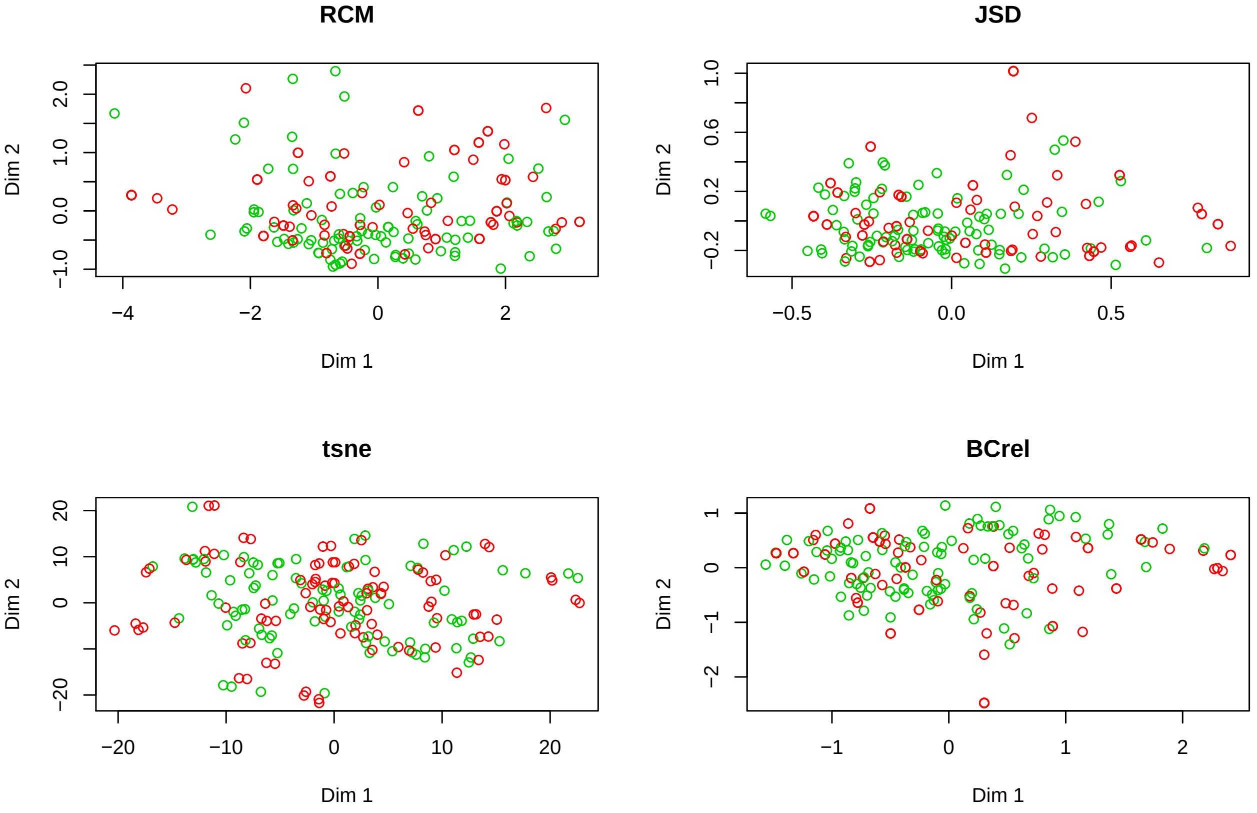

Figure S17: Sample plots of the ordinations by the RC(M), PCoA with Jensen-Shannon divergence, t-SNE and PCoA with Bray-Curtis distances on absolute abundances on a randomly sampeld SimSeq dataset generated based on the Kostic dataset with cancer diagnosis as grouping variable. Dots are coloured by cancer diagnosis.

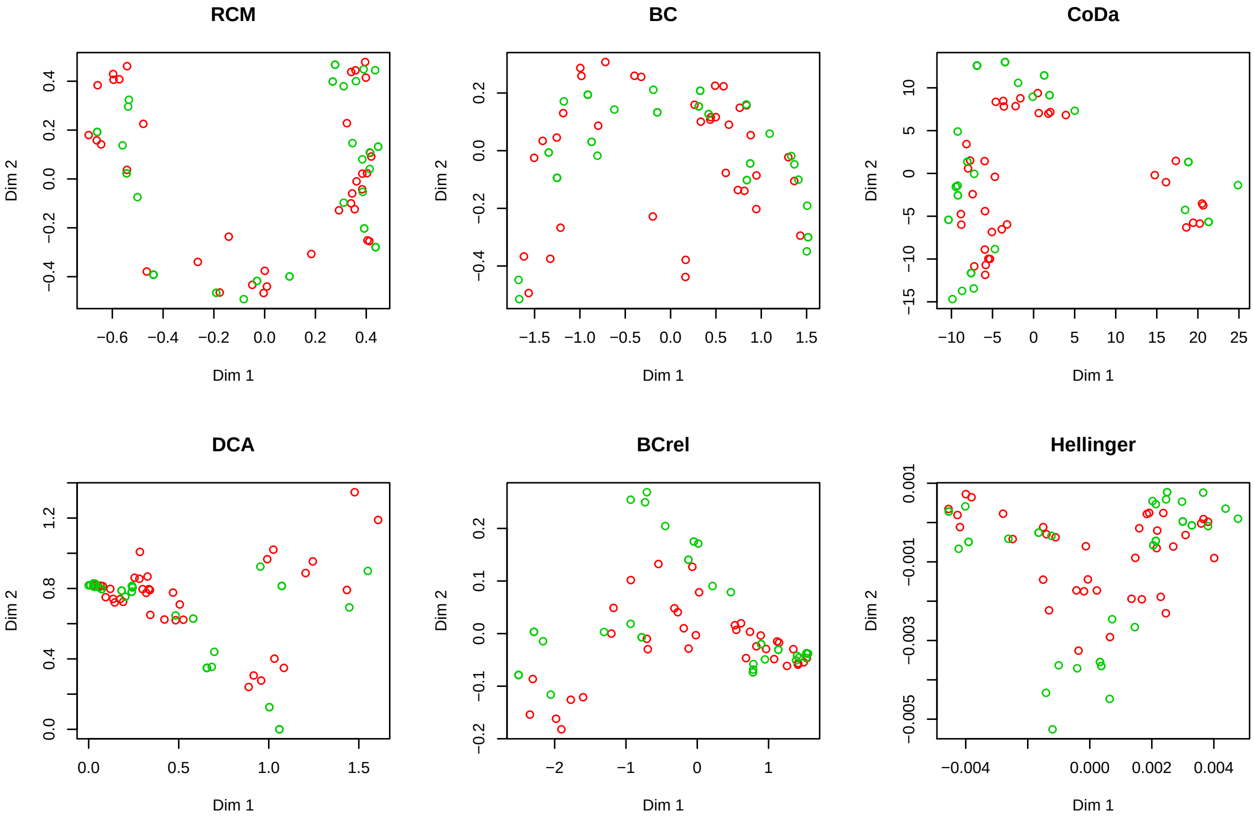

Figure S18: Sample plots of the ordinations by the RC(M), PCoA with Bray-Curtis distances on absolute abundances, CoDa, detrended correspondence analysis and PCoA with Bray-Curtis distances on relative abundances and Hellinger distance on a randomly sampeld SimSeq dataset generated based on the Props dataset with reactor cycle as grouping variable. Dots are coloured by reactor cycle.

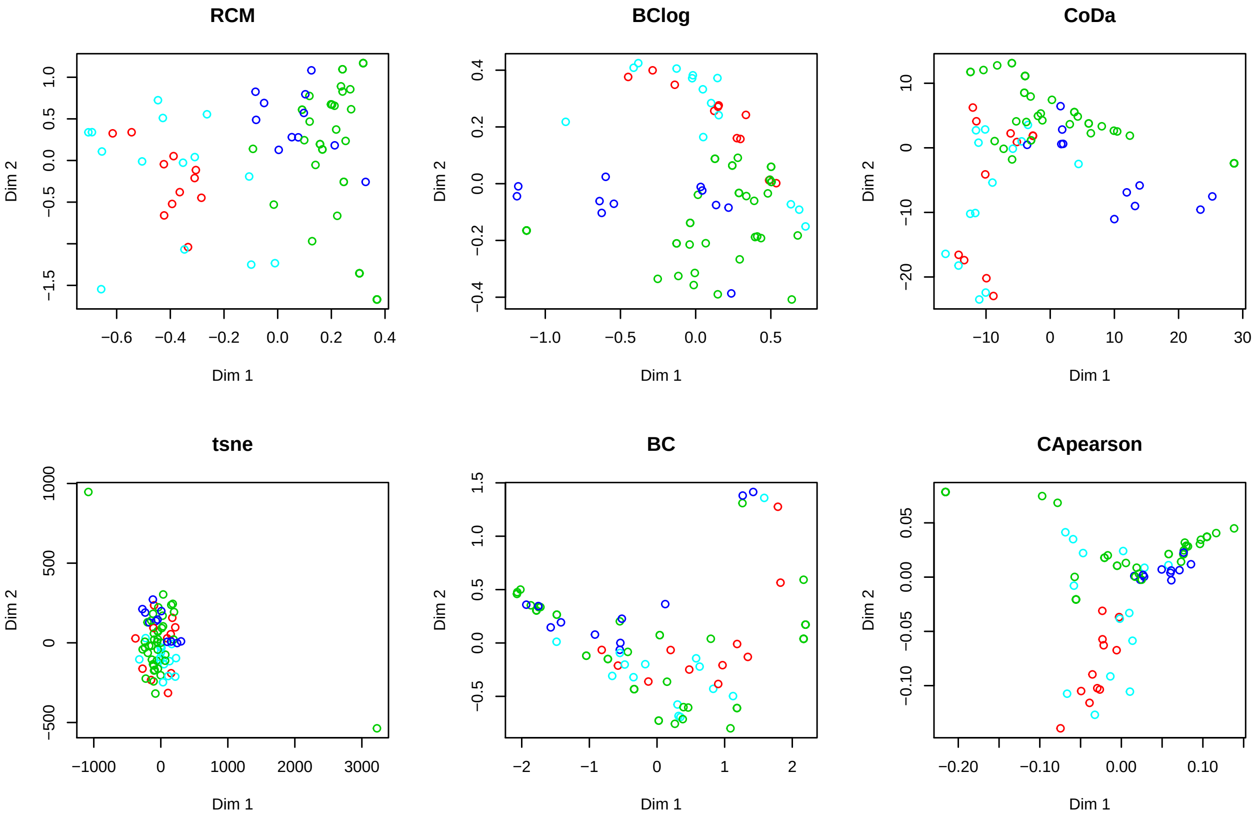

Figure S19: Sample plots of the ordinations by the RC(M), PCoA with Bray-Curtis distances on logged abundances, CoDa, t-SNE and PCoA with Bray-Curtis distances on absolute abundances and correspondence analysis based on Pearson residuals on a randomly sampeld SimSeq dataset generated based on the Props dataset with reactor phase as grouping variable. Dots are coloured by reactor phase.

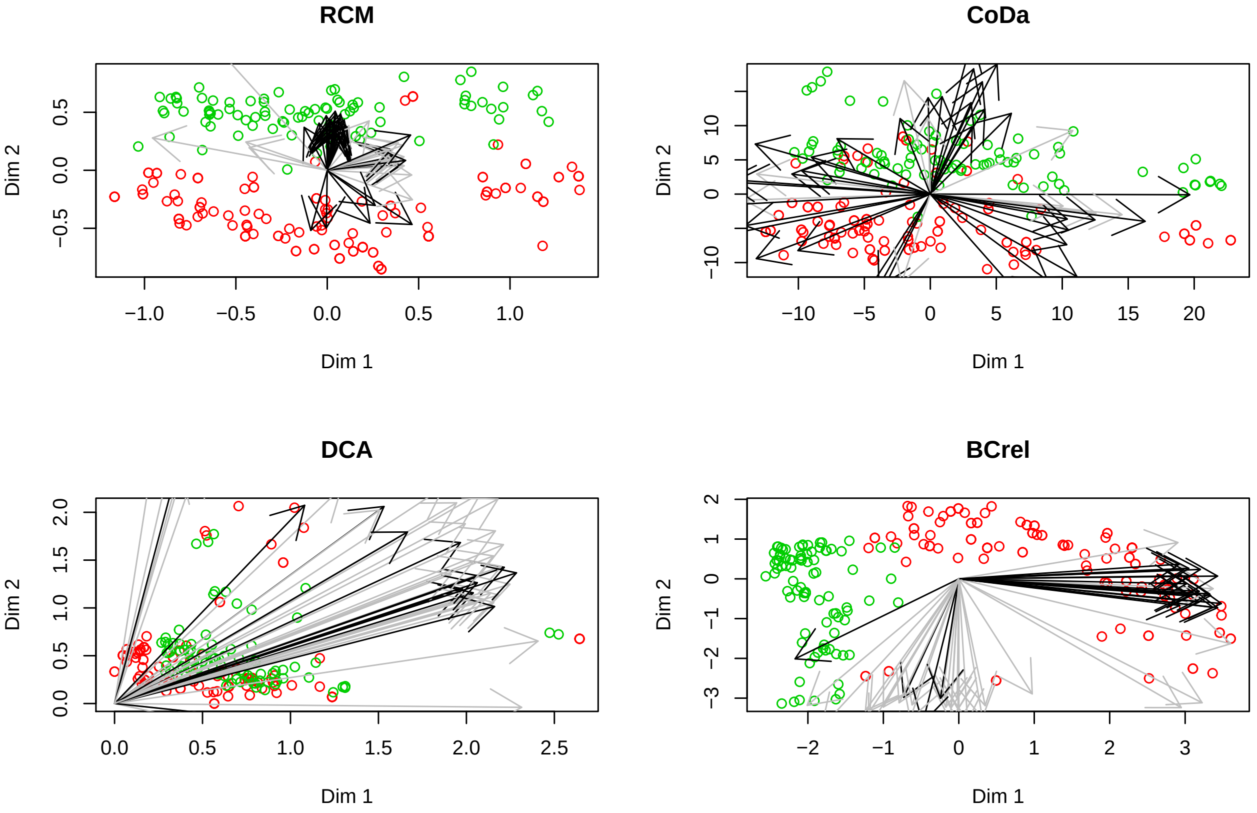

Figure S20: Biplots of the ordinations by the RC(M), CoDa, t-SNE and NMDS with Bray-Curtis distances on a randomly sampeld SimSeq dataset generated based on the Turnbaugh dataset with diet as grouping variable. Dots are coloured by diet, only the 5% taxa with strongest signal are shown. Differentially abundant taxa are coloured black, the others grey.

We can conclude that the high throughput measure of cluster sampling corresponds reasonably well with a visual evaluation. We next look at some biplots with median taxon ratio over all Monte-Carlo simulations to visually inspect if it is a good summary measure for taxon identification.

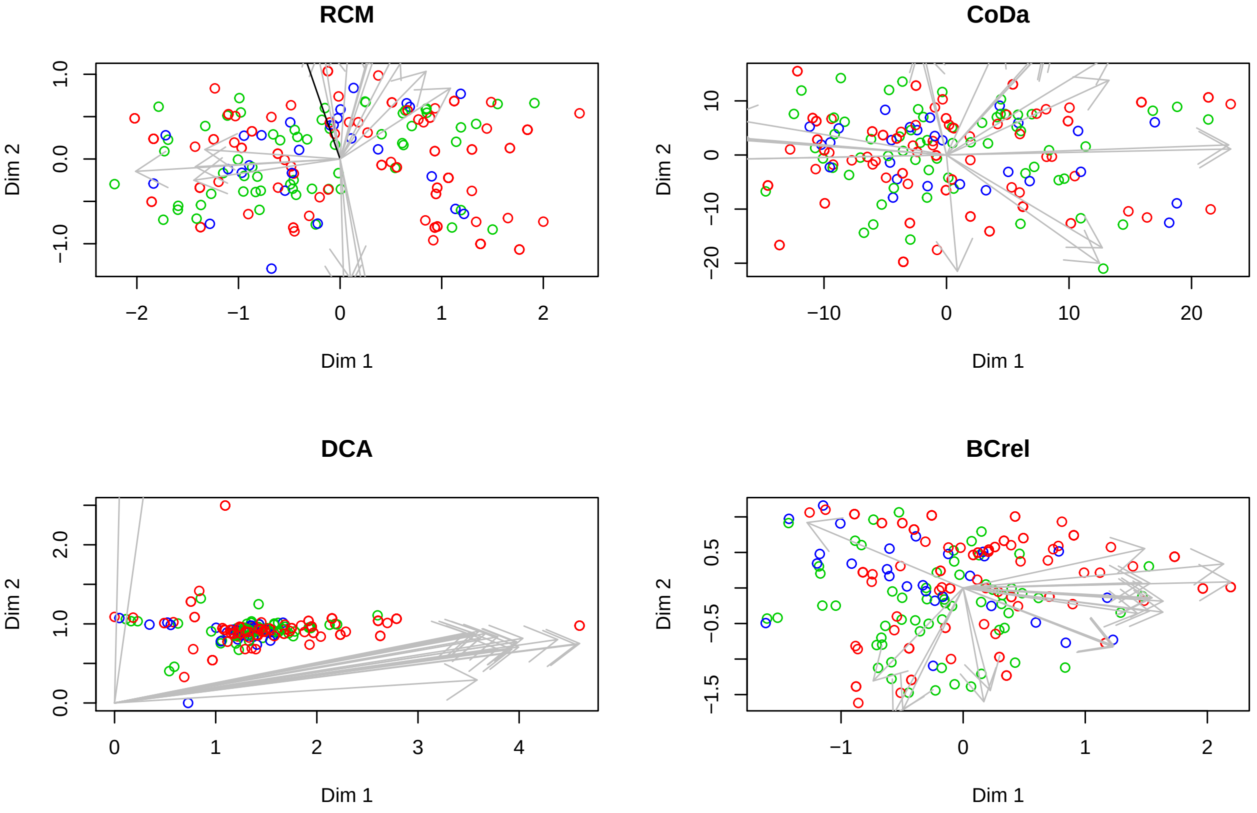

Figure S21: Biplots of the ordinations by the RC(M), CoDa, t-SNE and NMDS with Bray-Curtis distances on a randomly sampeld SimSeq dataset generated based on the Zeller dataset with diet as grouping variable. Dots are coloured by diet, only the 5% taxa with strongest signal are shown. Differentially abundant taxa are coloured black, the others grey.

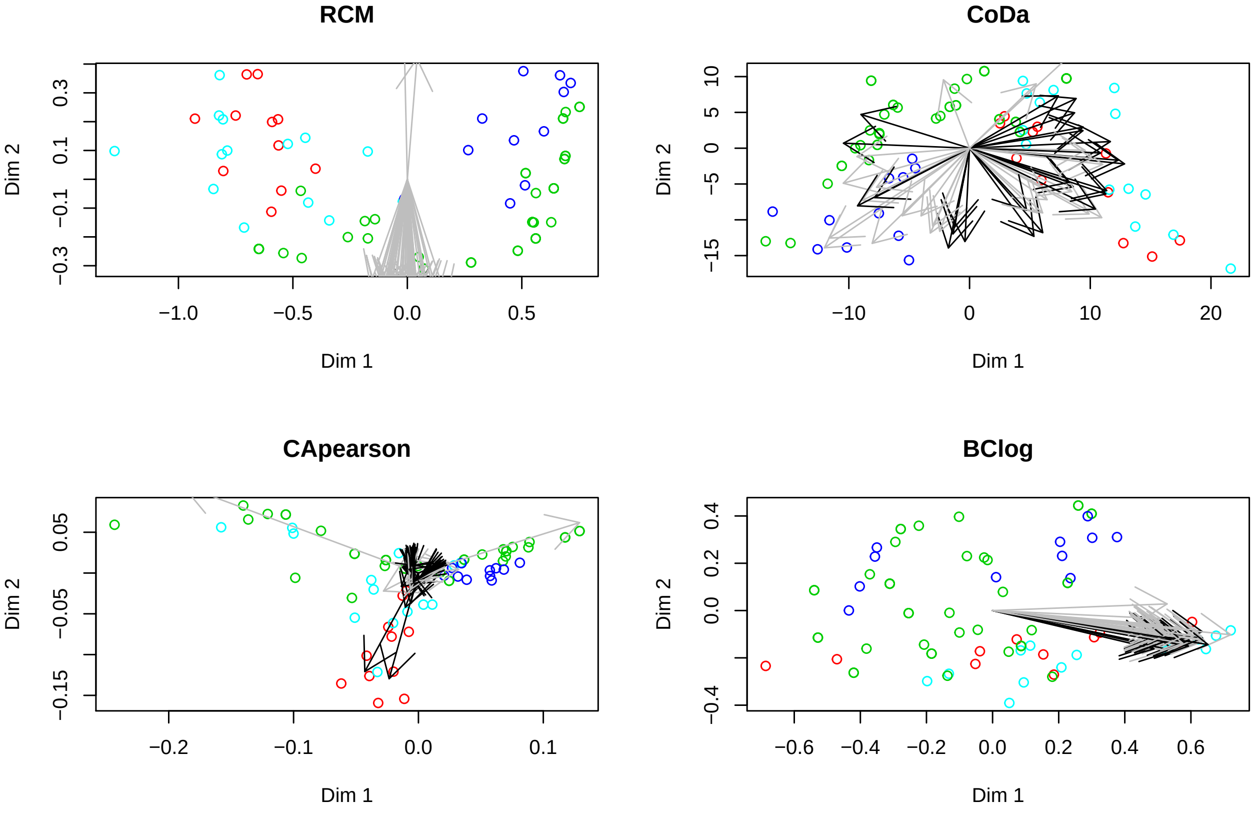

Figure S22: Biplots of the ordinations by the RC(M), CoDa, t-SNE and NMDS with Bray-Curtis distances on a randomly sampeld SimSeq dataset generated based on the CMETphase dataset with diet as grouping variable. Dots are coloured by diet, only the 5% taxa with strongest signal are shown. Differentially abundant taxa are coloured black, the others grey.

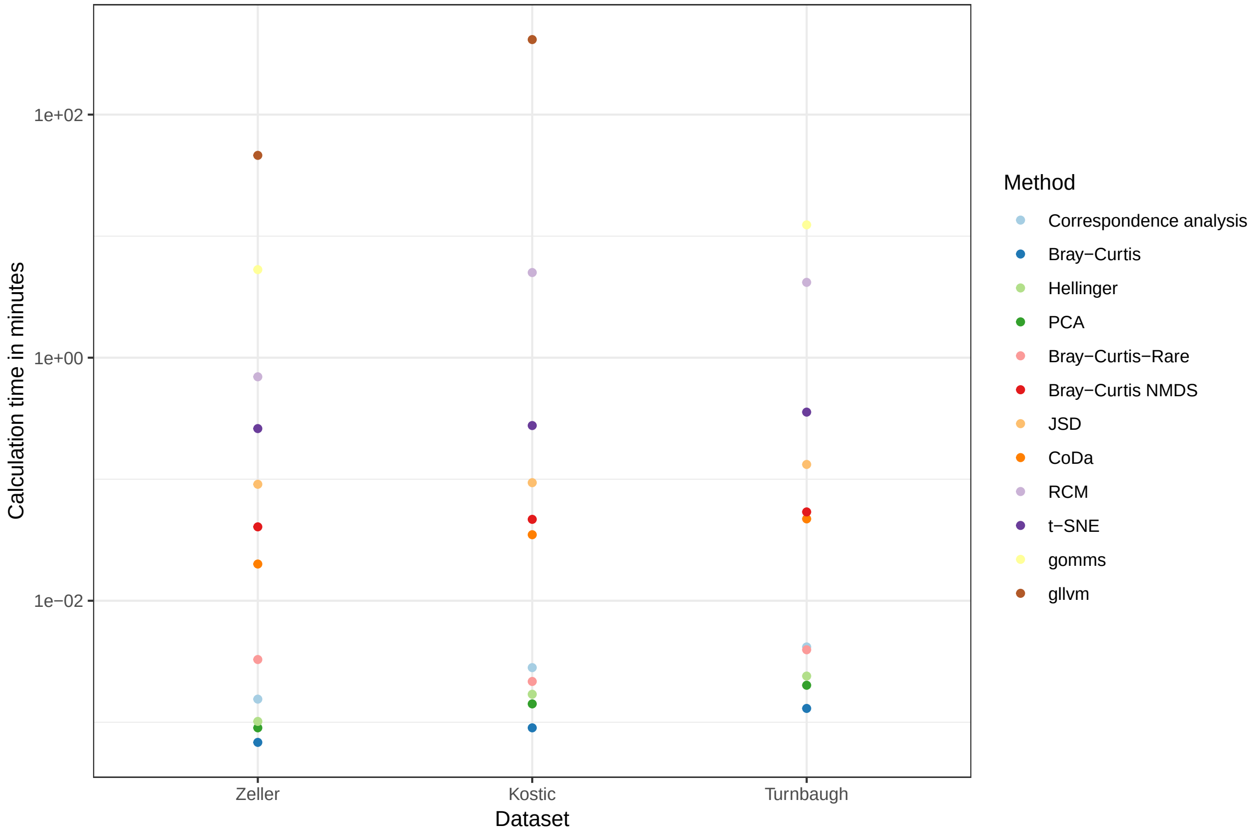

Figure S23: Benchmark of computation times of different methods on the Zeller, Kostic and Turnbaugh datasets. The gomms did not converge for the Kostic dataset, the gllvm method not for the Turnbaugh dataset. The fits of VGAM and logmult all failed because of memory problems. The *boral* package would not converge after >18h and timing was stopped for all three datasets.

3.5 Computational benchmark

We illustrate the computational efficiency in terms of user time of the different methods used by benchmarking them on the Zeller, Turnbaugh and Kostic datasets. The results are shown in Figure S23 and Table S6.

The *VGAM* and *logmult* packages also implement the RC(M) model with negative binomial error model, but with slight differences in restrictions on the parameters. Both packages fail to converge for the microbiome datasets under study, due to memory problems.

% latex table generated in R 3.5.1 by xtable 1.8-3 package % Fri Dec 7 16:11:36 2018

3.6 Failed fits

Another measure of performance is the amount of failed fits. Failed fits only occur for model-based approaches, so we limit the discussion to the *RCM*, *gomms* and *gllvm* methods.

% latex table generated in R 3.5.1 by xtable 1.8-3 package % Sat Dec 8 12:53:46 2018

3.7 Summary table

% latex table generated in R 3.5.1 by xtable 1.8-3 package % Sat Dec 8 12:55:05 2018

	Zeller	Kostic	Turnbaugh
RCM	00:00:42	00:05:01	00:04:10
CA	00:00:00	00:00:00	00:00:00
DCA	00:00:00	00:00:00	00:00:00
CoDa	00:00:01	00:00:02	00:00:03
PCA	00:00:00	00:00:00	00:00:00
BC	00:00:00	00:00:00	00:00:00
BClog	00:00:00	00:00:00	00:00:00
JSD	00:00:05	00:00:06	00:00:08
BCrel	00:00:00	00:00:00	00:00:00
BCrare	00:00:00	00:00:00	00:00:00
BCrelNMDS	00:00:02	00:00:03	00:00:03
Hellinger	00:00:00	00:00:00	00:00:00
tsne	00:00:16	00:00:17	00:00:21
gomms	00:05:18	Fit failed	00:12:23
gllvm	00:46:13	06:54:26	Fit failed
logmult	Out of memory	Out of memory	Out of memory
VGAM	Out of memory	Out of memory	Out of memory
boral	>18h	>18h	Out of memory

Table S6: Summary table of fitting times or different methods on the Zeller, Kostic and Turnbaugh datasets. Time formats are hh:mm:ss.

4 Real data examples

We apply the RC(M) method to a number of real datasets to illustrate its functionality and prove that it yields biologically valid results.

The Human Microbiome Project (HMP, V13 region of the 16S rRNA gene) (Peterson et al. 2009) and the American Gut Project (AGP) (AmericanGut.org 2015) provide microbiome count datasets of healthy human volunteers. Data from two studies on the colorectal microbiome of cancer patients, referred to as the Zeller data (Zeller et al. 2014) and the Kostic data (Kostic et al. 2012) are also included. A study on several generations of gnotobiotic mice, referred to as the Turnbaugh data (Turnbaugh et al. 2009), provides non-human microbiome data. A study on microbes in cooling water provides data from a non-mammalian source, referred to as the Props data (Props et al. 2016).

4.1 Human microbiome project

The Human Microbiome Project (HMP) aimed to characterize the healthy human microbiome (Peterson et al. 2009). 18 body sites were sampled, here we only show the results of the samples originating from the Anterior nares (nasal cavity). Because of the low number of recorded variables, only an unconstrained RC(M)-model was fitted.

Since we know that sequencing facility affects the outcome of the sequencing assay, and one is not interested in visualizing this technical variability, we can condition out this variable. We condition only on the primary sequencing center (Washington University genome center (WUGC), J. Craig Venter Institute (JCVI), Baylor College of Medicine (BCM) and Broad Institute (BI)).

	RCM	gomms	gllvm
0	1.00	0.16	1.00
0b	0.96	0.10	1.00
4	1.00	0.08	1.00
1	0.99	0.74	1.00
2	1.00	0.18	1.00
3	1.00	0.21	1.00
5	1.00	0.11	1.00
Phy	1.00	0.16	1.00
DM	1.00	0.44	1.00
ZINB	1.00	0.09	1.00
3b	1.00	0.46	1.00
4b	1.00	0.05	1.00
CMETcycle	1.00	0.00	0.00
CMETphase	1.00	0.02	0.00
KosticDiagnosis	1.00	0.11	1.00
KosticCountry	1.00	0.01	1.00
Zeller	0.94	0.89	1.00
Turnbaugh	1.00	0.60	1.00

Table S7: Fraction of succesfull fits over all generated datasets in parametric and non-parametric simulations for the RCM, gomms and gllvm methods

	PCoA	Correspondence analysis	CoDa	Latent variable models	RC(M)
Discriminative power	Mediocre	Mediocre	Good	Good	Good
Direct taxon identification	No (only PCA)	Yes	Yes	Yes	Yes
Sensitivity to library sizes	Some distance measures	None	Yes	No	No
Sensitivity to sample-wise dispersions	Some distance measures	None	Yes	Yes	Yes
Conditioning on known confounders	No	Yes	No	Yes	Yes
Constrained counterpart	Two-step approach	Yes	No	No	Yes
Fitting time	Seconds	Seconds	Seconds	Minutes-Hours	Minutes
Goodness-of-fit checks	No	No	No	Yes	Yes

Table S8: Summary table on the strengths and weaknesses of several families of ordination methods considered

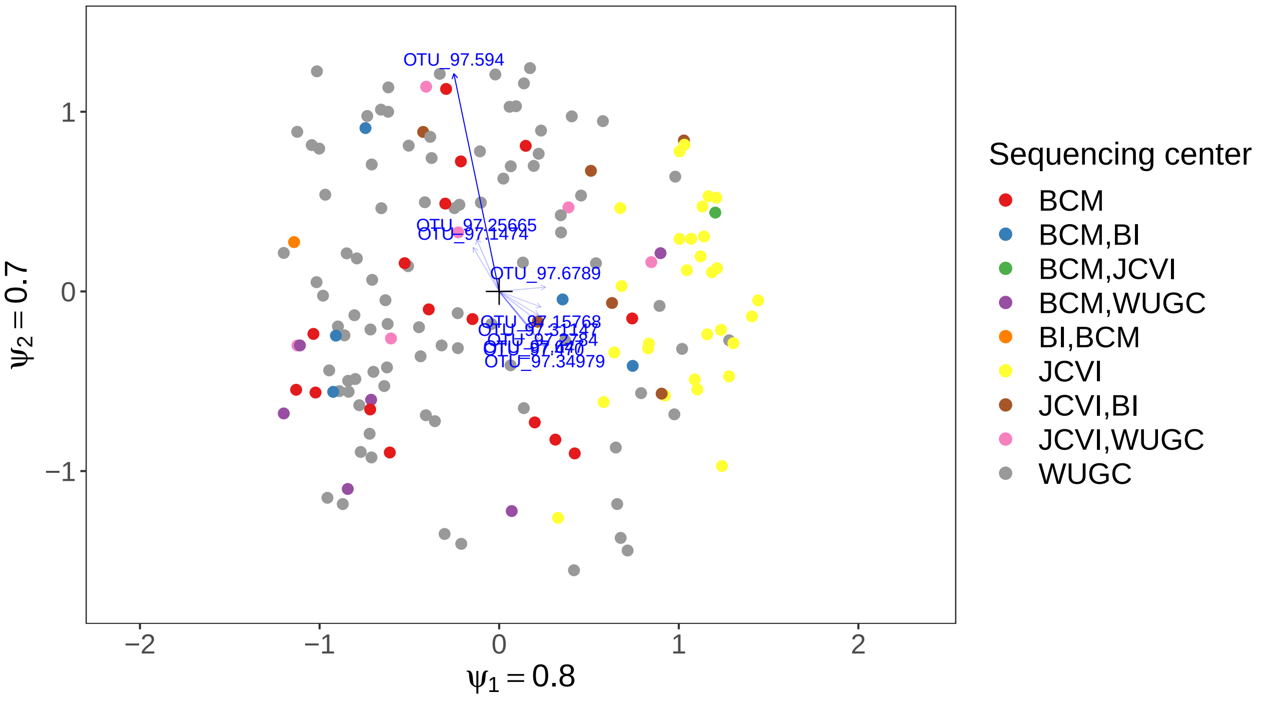

Figure S24: Biplot of the RC(M) ordination of the Anterior nares dataset of the HMP. Colours indicate sequencing center. Sequencing center clearly affects the obtained microbiome compositions.

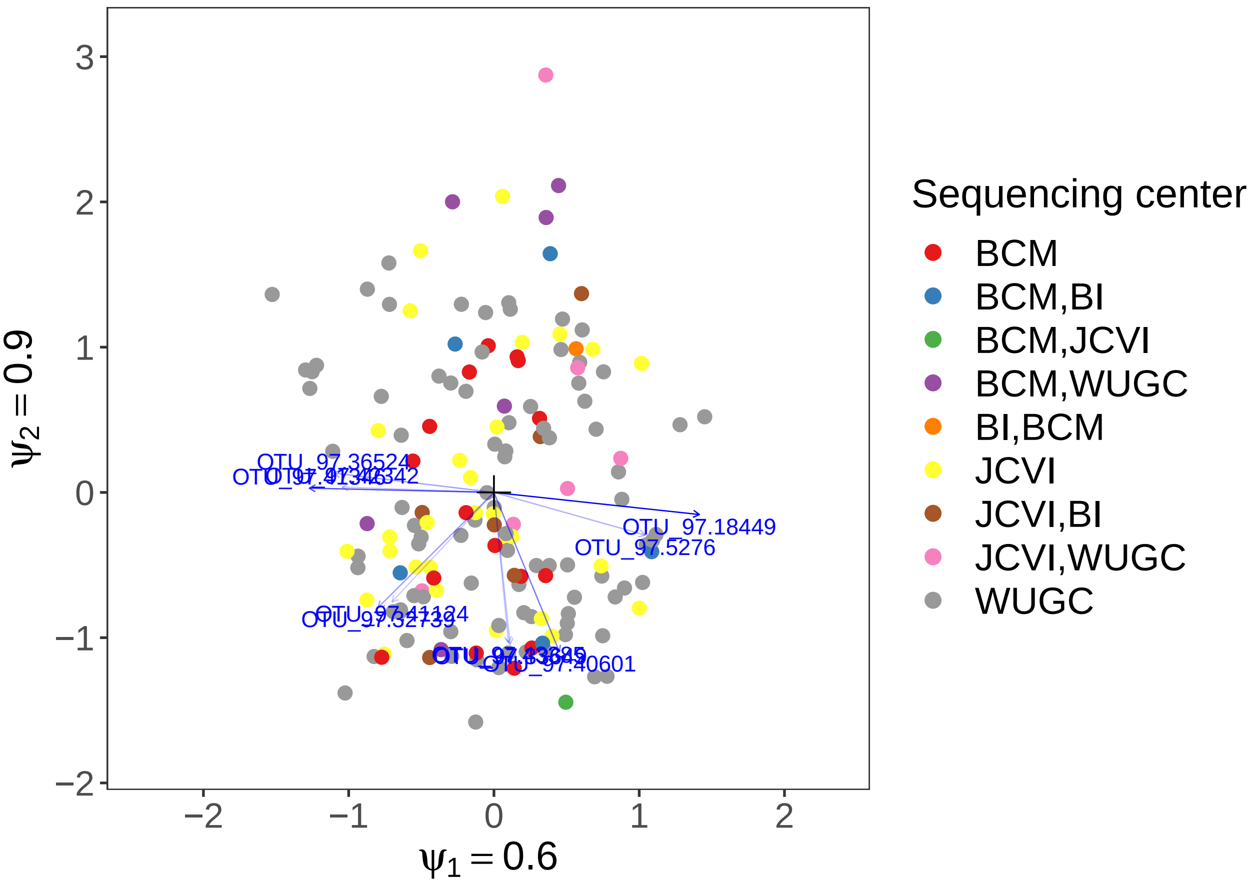

Figure S25: Biplot of the RC(M) ordination of the HMP Anterior nares dataset after conditioning on main sequencing center (Washington University genome center (WUGC), J. Craig Venter Institute (JCVI), Baylor College of Medicine (BCM) and Broad Institute (BI)). The effect of sequencing center has been largely filtered out.

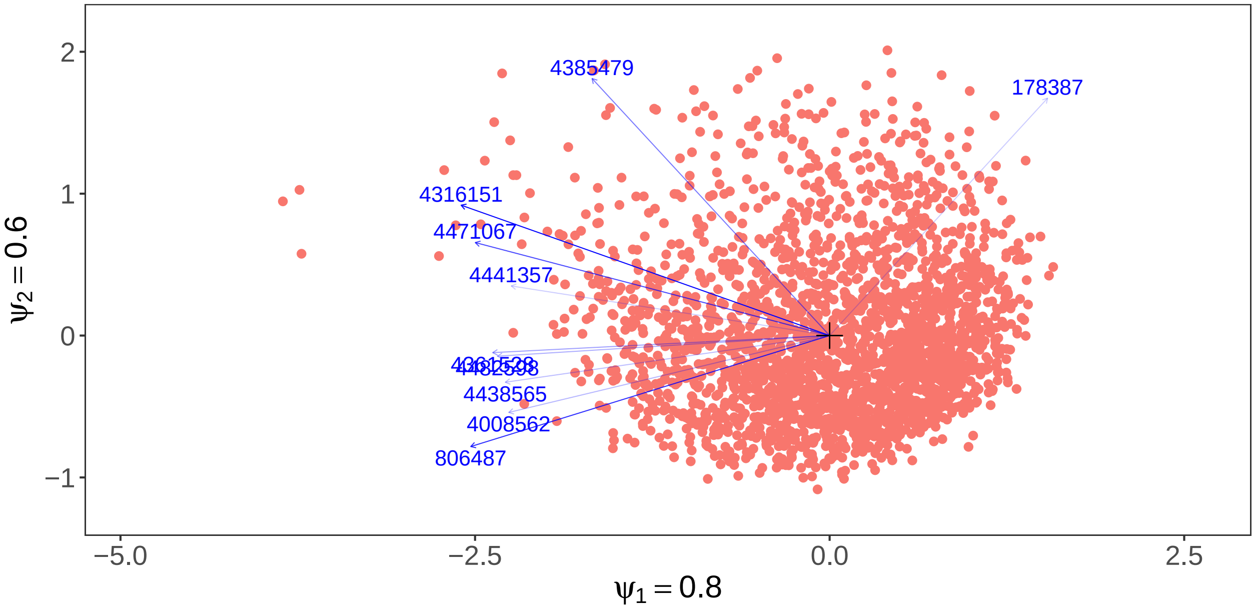

Figure S26: Biplot of the RC(M) ordination of the American gut dataset. As expected the dataset is very noisy and we do not find a clear signal.

4.2 The American gut project

The American gut project consists of stool samples sampled by volunteers at home, together with their answers to a questionnaire (AmericanGut.org 2015). Since they are sampled at home the variability is expected to be large. Only an unconstrained RC(M) model was fitted.

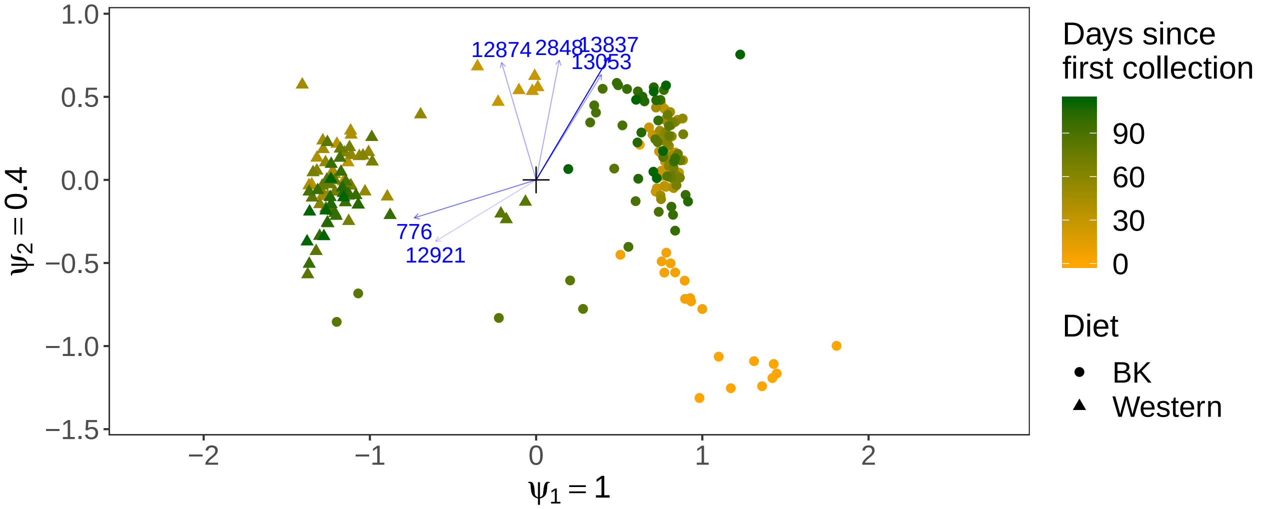

Figure S27: RC(M) biplot of Turnbaugh data. This dataset appears to have a very strong signal, we can distinguish at least two clusters. The separation in the first dimension is clearly an effect of Diet, the second dimension seems to be dominated by the date of collection

4.3 Turnbaugh et al. (2009)

In this study, 15 gnotobiotic mice were inoculated with human feces, and for one group the diet was switched to a Western diet after one month (Turnbaugh et al. 2009) (Turnbaugh data). Then a second generation of mice was inoculated with cecal samples from the previous groups, and here also diet was varied. The variables recorded were:

- Diet: current diet
- Generation_p: "Recipient1" if second generation, otherwise diet of cecal samples with which they were inoculated
- Time: age of mouse at sampling
- DGS: Diet and previous diet
- DTG: combines sampling diet, time, and previous generation (if any)

The number of (independent) variables is too small for a constrained analysis, only an unconstrained RC(M) model was fitted.

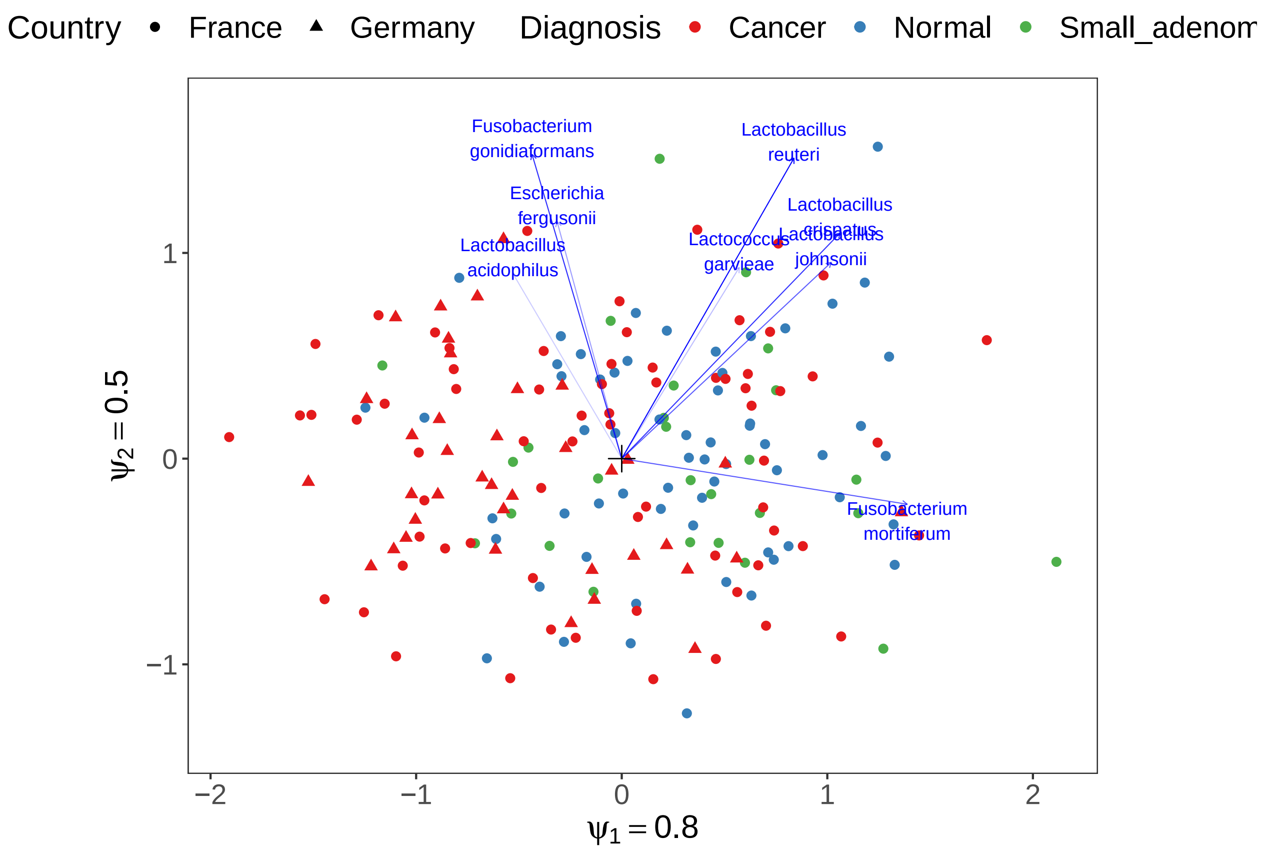

Figure S28: Biplot of the RC(M) ordination of the 16S Zeller data. We see some gradient as a function of cancer diagnosis, but there is still a lot of remaining variability. These is consistent with the findings of the authors Zeller et al. 2014.

4.4 Zeller et al. (2014)

4.4.1 Unconstrained RC(M)

The Zeller data are obtained from a study on colorectal cancer in cancer patients and healthy controls (Zeller et al. 2014). Patient covariates recorded were age, gender, BMI, cancer diagnosis (healthy, small adenoma or cancer) and country (France or Germany). On the same data, 16S rRNA as well as metagenomic data are available.

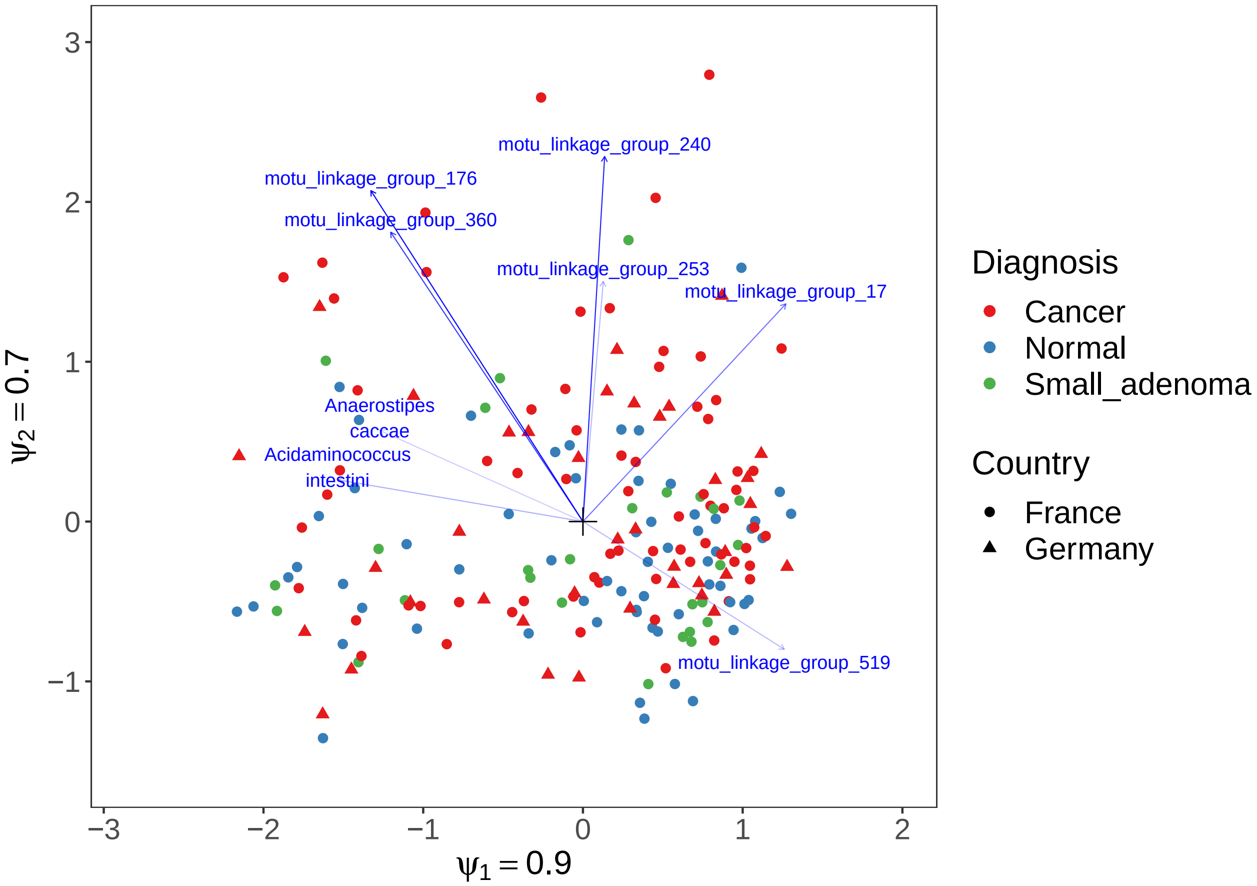

Figure S29: Biplot of the RC(M) ordination of the metagenomics Zeller data. No clear gradient related to the diagnosis is visible.

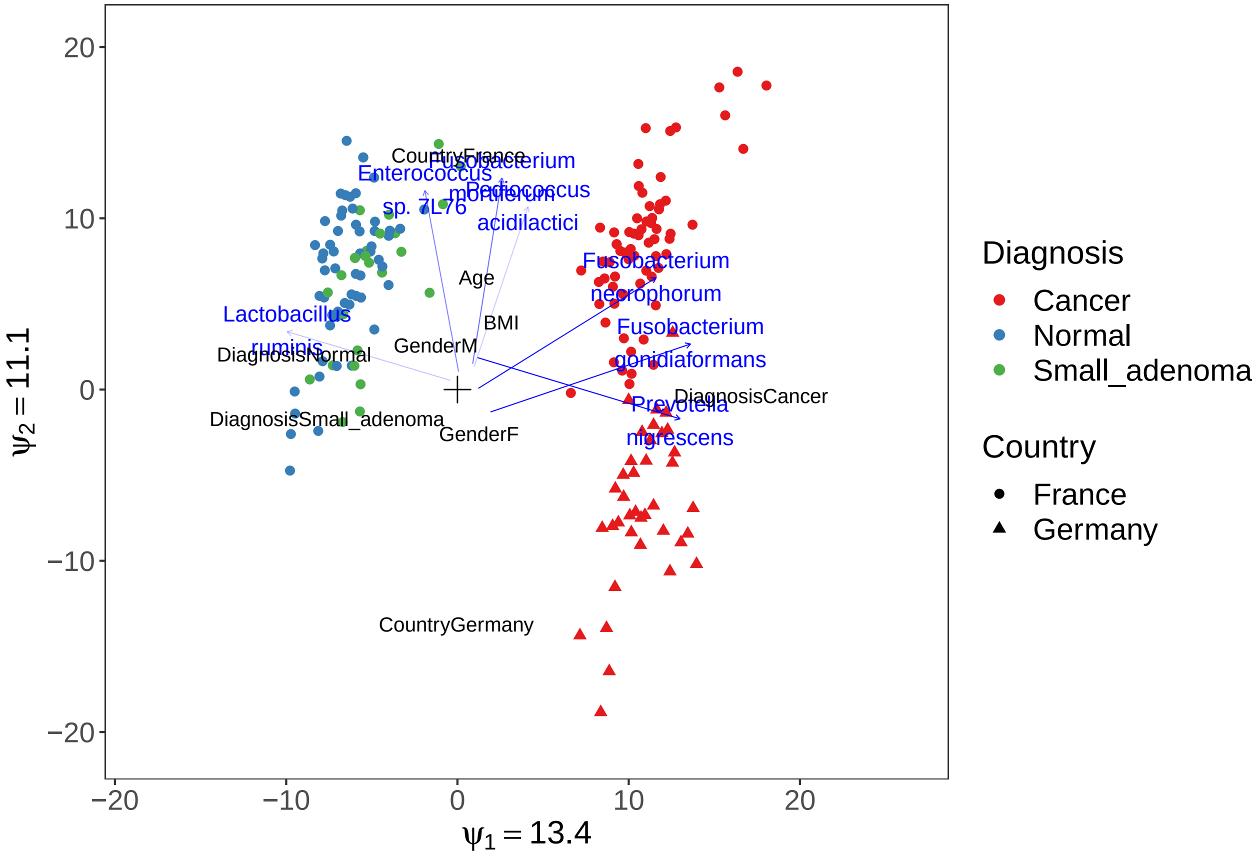

Figure S30: Triplot of constrained RC(M) analysis on 16S Zeller data with linear response functions. Cancer diagnosis and country appear to be the most important variables. The fact that this signal is much less clear in the unconstrained analysis suggests that the dataset contains a lot of variability that cannot be explained by the recorded variables.

4.4.2 Constrained RC(M)

In the constrained analysis of the Zeller data we used all the available covariates to construct the environmental gradient: age, gender, diagnosis, BMI and country.

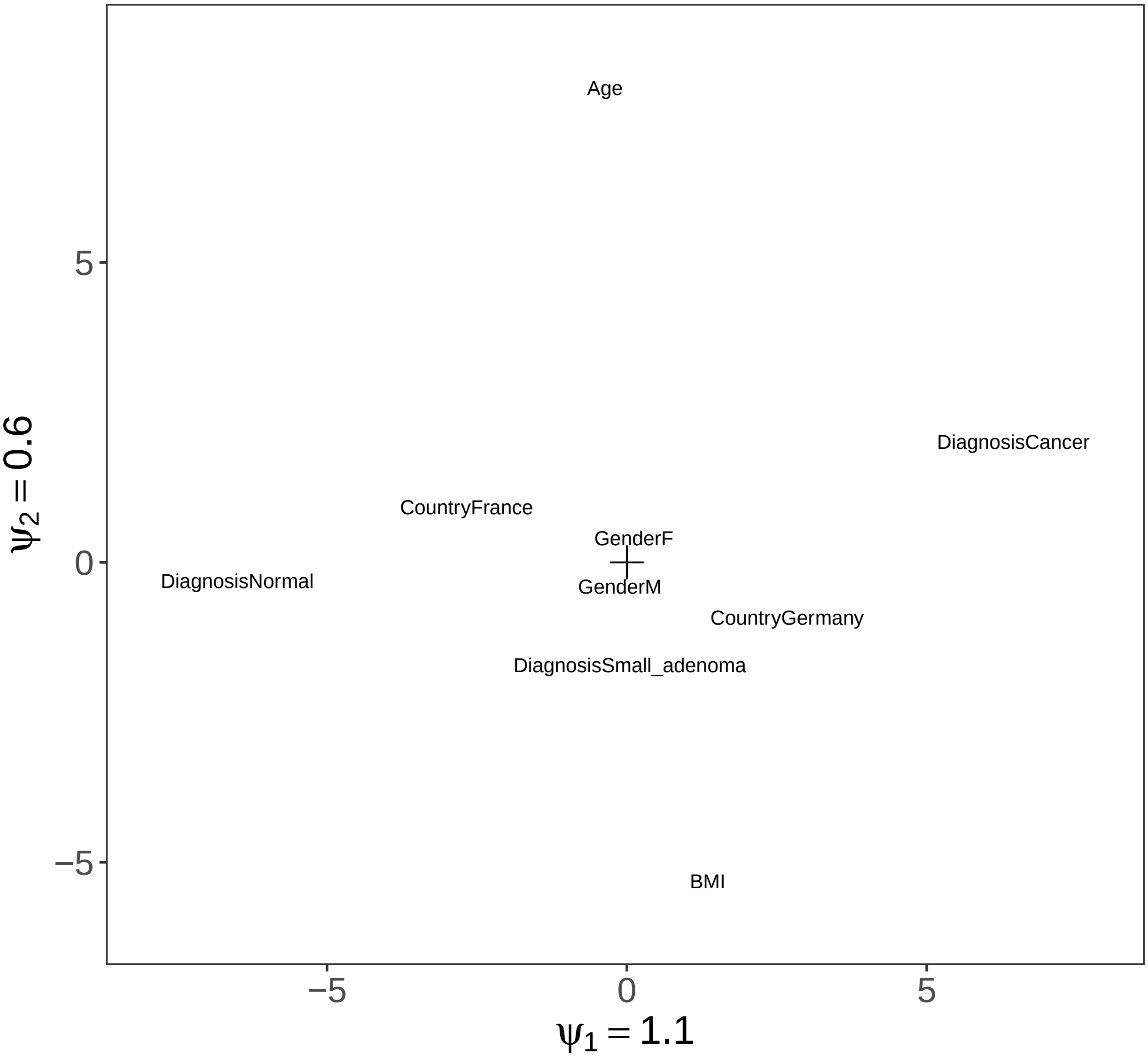

Figure S31: Monoplot of the environmental gradient of the constrained RC(M) analysis on 16S Zeller data with non-parametric response functions. The environmental gradient with non-parametric response functions is similar as to the linear case, only age is more important according to this model. Note that distances between the variables are meaningless, each dimension of the gradient should be interpreted separately.

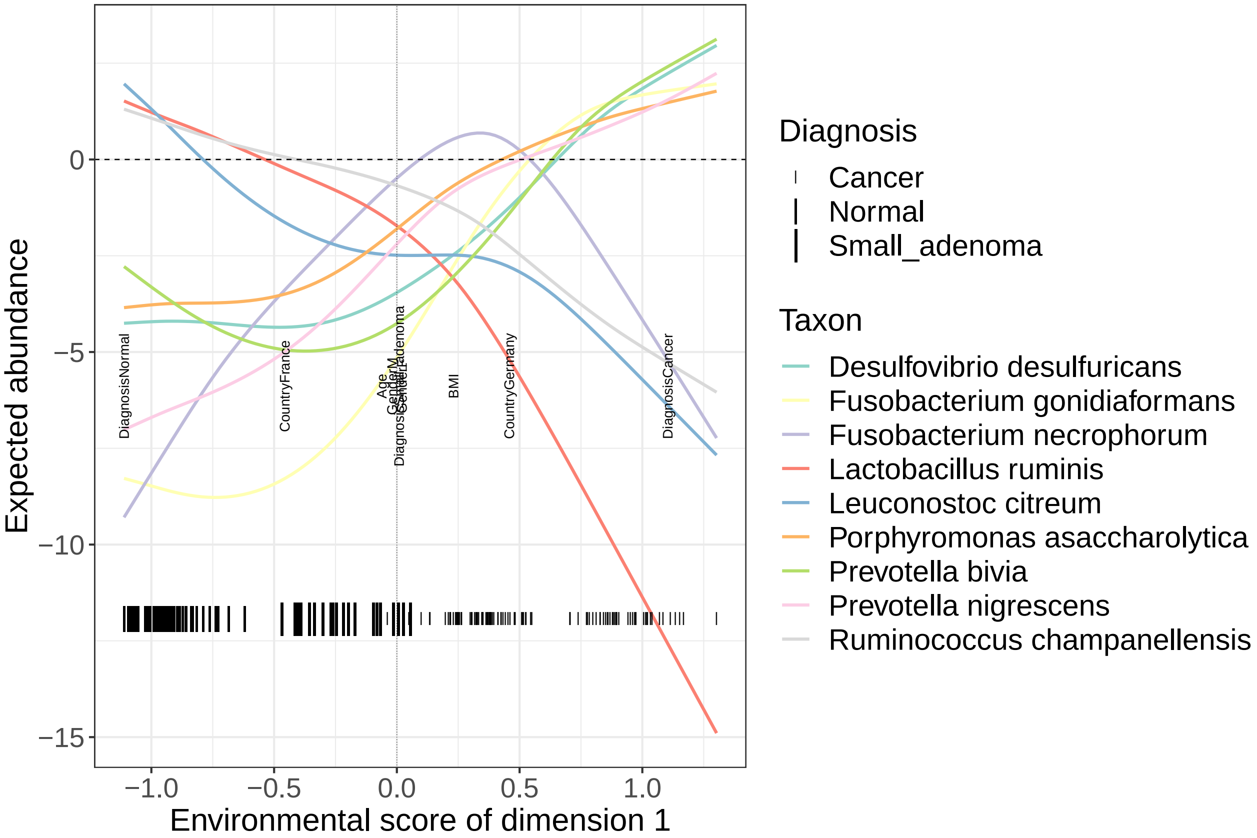

Figure S32: Non-parametric response functions of constrained RC(M) analysis on 16S Zeller data. The x-axis shows the values of the environmental gradient in the first dimension, with the observed values of this gradient shown as dashes below. The size of the dashes indicate the cancer diagnosis. The black labels show the contribution of the different variables to the gradient. It is clear that cancer diagnosis is a crucial variable for this gradient. The y-axis depicts the value of the response function, with the dashed line at 0 representing the homogeneity model. The coloured lines show the value of the taxon response function along the gradient. Only the 9 most strongly reacting taxa are shown. We can distinguish three different types of response functions among the 9 most responsive taxa: One group of taxa with decreasing abundance along the gradient, one group with increasing abundance and the *Neisseria* species has a unimodal response function that rises and drops along the gradient.

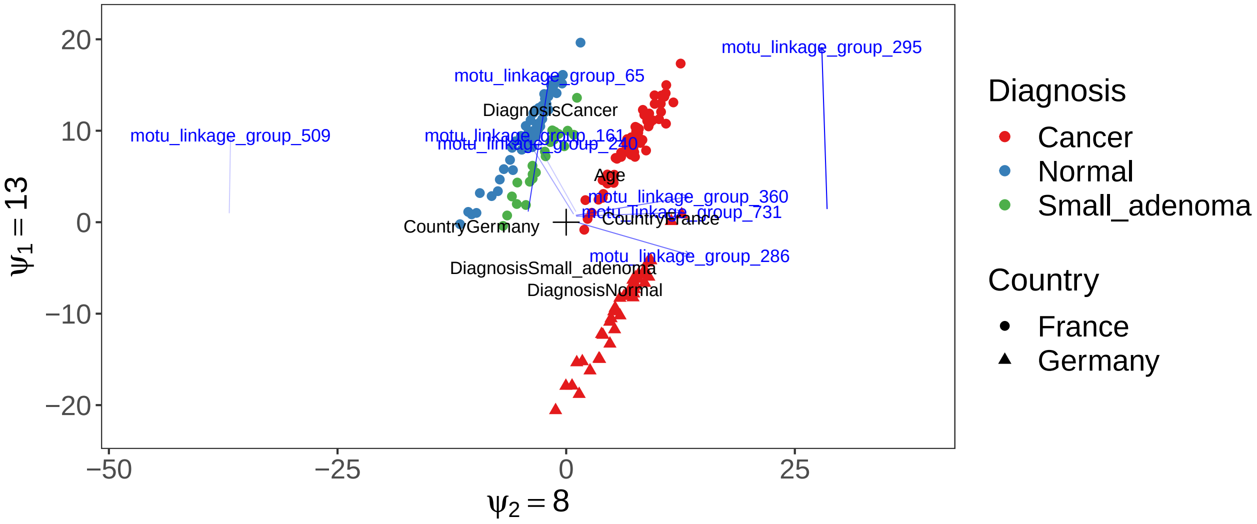

Figure S33: Triplot of constrained RC(M) analysis on metagenomic Zeller data with linear response functions. Cancer diagnosis and country appear to be the most important variables. The results from metagenome sequencing are very different from those from 16S rRNA sequencing. Country still plays an important role in the ordination, but the different diagnosis groups are not separated as clearly anymore. One mOTU seems completely out of touch with the rest.

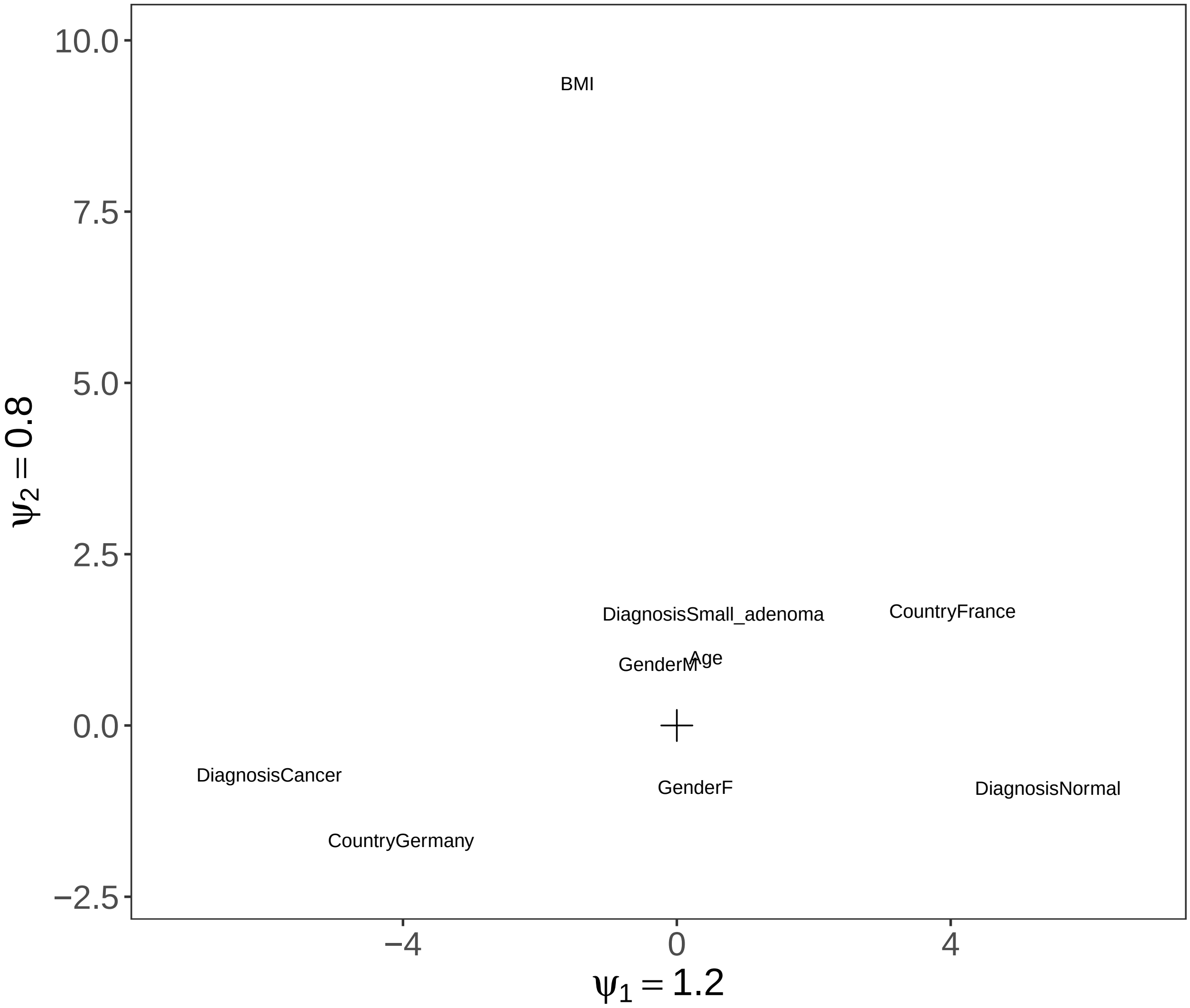

Figure S34: Monoplot of constrained RC(M) analysis on metagenomic Zeller data with non-parametric response functions. Cancer diagnosis dominates the first dimension, BMI the second.

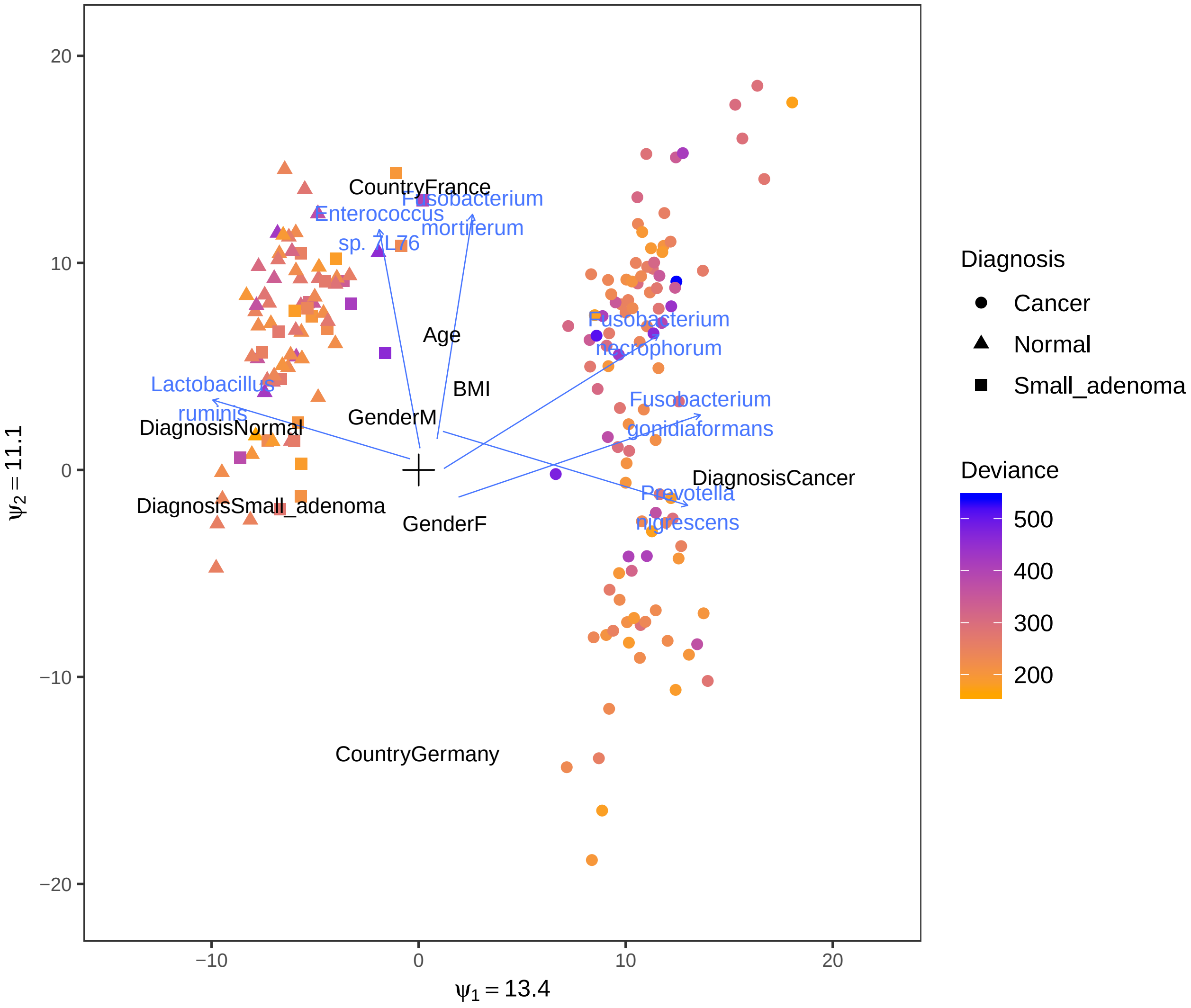

Figure S35: Constrained ordination plot of the Zeller data with linear response functions. Samples are coloured by mean deviance. There are no samples with exceptionally high deviances, nor any clusters of these samples. Still the samples with the highest deviance may deserve closer scrutiny.

4.4.3 Diagnostic plots

Assumptions and outlying observations can be further investigated using diagnostic plots.

4.4.3.1 Deviances

4.4.3.2 Influential observations

We investigate which samples have on average the strongest influence on the estimation of the age parameter in the environmental gradient in the first dimension

4.4.3.3 Residual plots

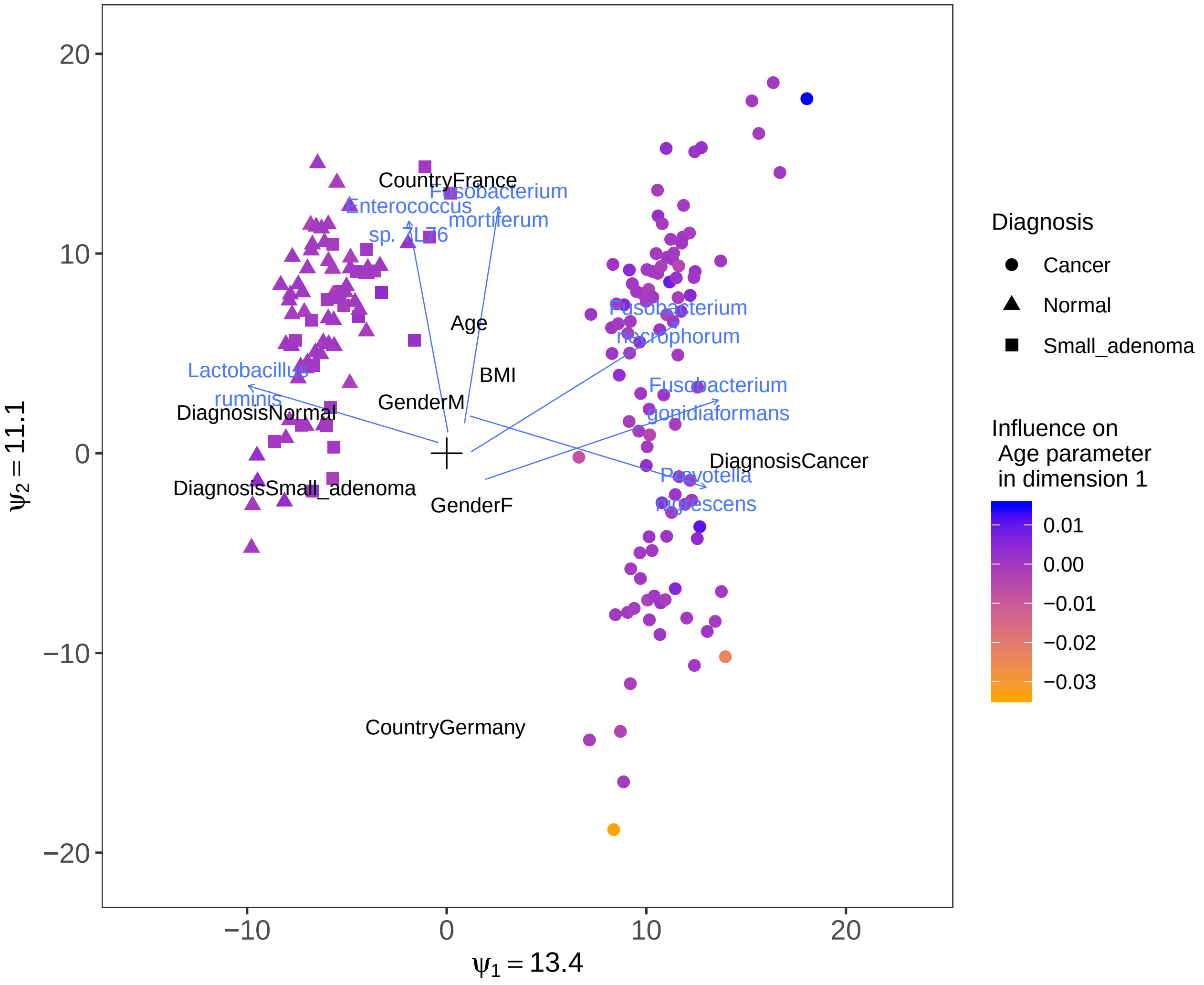

Figure S36: Constrained ordination plot of the Zeller data with linear response functions. Samples are coloured by influence on the estimation of the age parameter in the first dimension. As expected, samples from old people (on top) and young people (bottom) have the strongest influence on the estimation of this parameter, although no single sample has an extremely high influence.

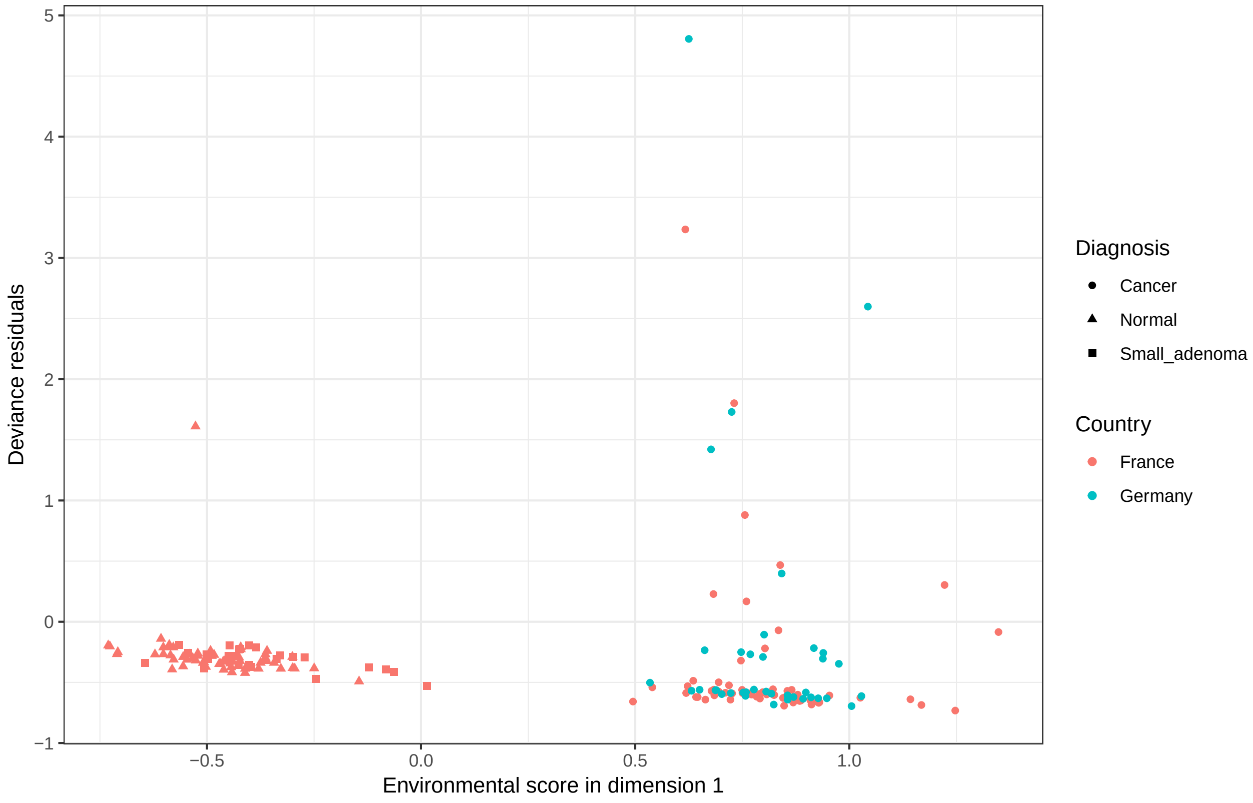

Figure S37: Deviance residuals of the RC(2) ordination of the Zeller data for Fusobacteruim gonidiaformans as a function of the environmental score in the first dimension. For this taxon there is a clear trend of more large values and an overall decrease in the size of the residuals with increasing environmental score. The linearity assumption may not be valid for this taxon in this dimension, and results of the ordination should be interpreted with caution.

Residual plots help to assess the assumption on the shape of the response function. Constrained RC(M) models with linear response functions are easy to plot, but their interpretation depends on the validity of the linearity assumption. To check this we can plot the residuals as a function of the regressor (the environmental score in the first dimension). There must not be a visible pattern of residuals as a function of this score. We pick the taxon with the highest deviance (*Fusobacterium gonidiaformans*) to plot as an illustration.

4.5 Kostic et al. (2012)

This is a study on the microbiome of colorectal cancer in humans (Kostic et al. 2012). Nine cancer patients were matched with 9 healthy patients, samples were taken repeatedly. The researchers find a enrichment of Fusobacteria in the cancer patients and a depletion of Bacteroidetes and Firmicutes.

For the constrained analysis we use the variables

- NECROSIS_PERCENT
- AGE
- NORMAL_EQUIVALENT_PERCENT
- FIBROBLAST_AND_VESSEL_PERCENT
- TREATMENT
- CEA (Carcinoembryonic antigen)
- SEX
- COUNTRY
- CHEMOTHERAPY
- HISTOLOGIC_GRADE
- TUMOR_PERCENT
- RADIATION_THERAPY
- INFLAMMATION_PERCENT
- PC3 (a prostate cancer cell line)

For radiation therapy and chemotherapy, "None" and "No" were pooled. For Necrosis percent, normal equivalent percent, tumor percent, inflammation percent and CEA, "None" was set to 0, for age to the average age. Necrosis percent, age, normal equivalent, fibroblast and vessel percent, and CEA were treated as continuous variables

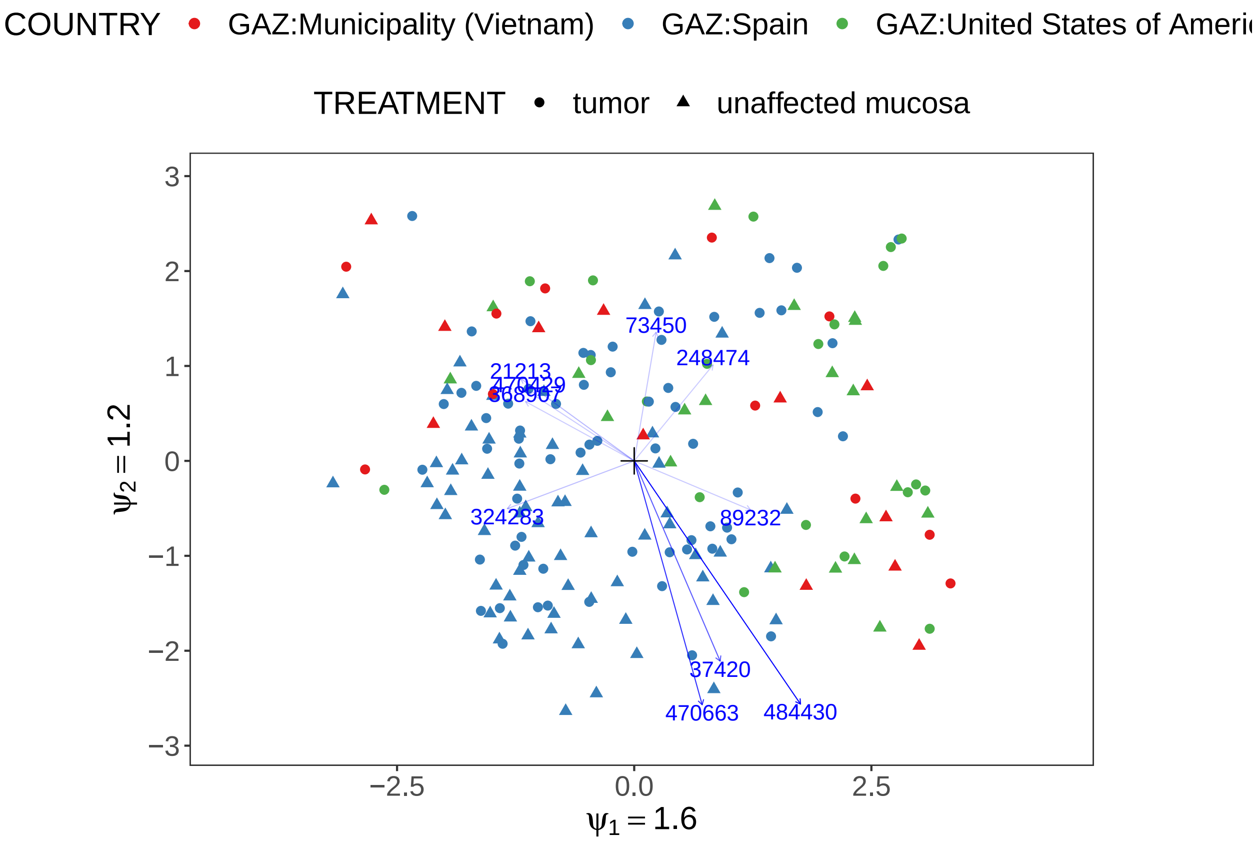

Figure S38: Unconstrained RC(M) biplot of Kostic data. Country, chemotherapy and radiotherapy seem to be related to microbiome composition from the unconstrained analysis.

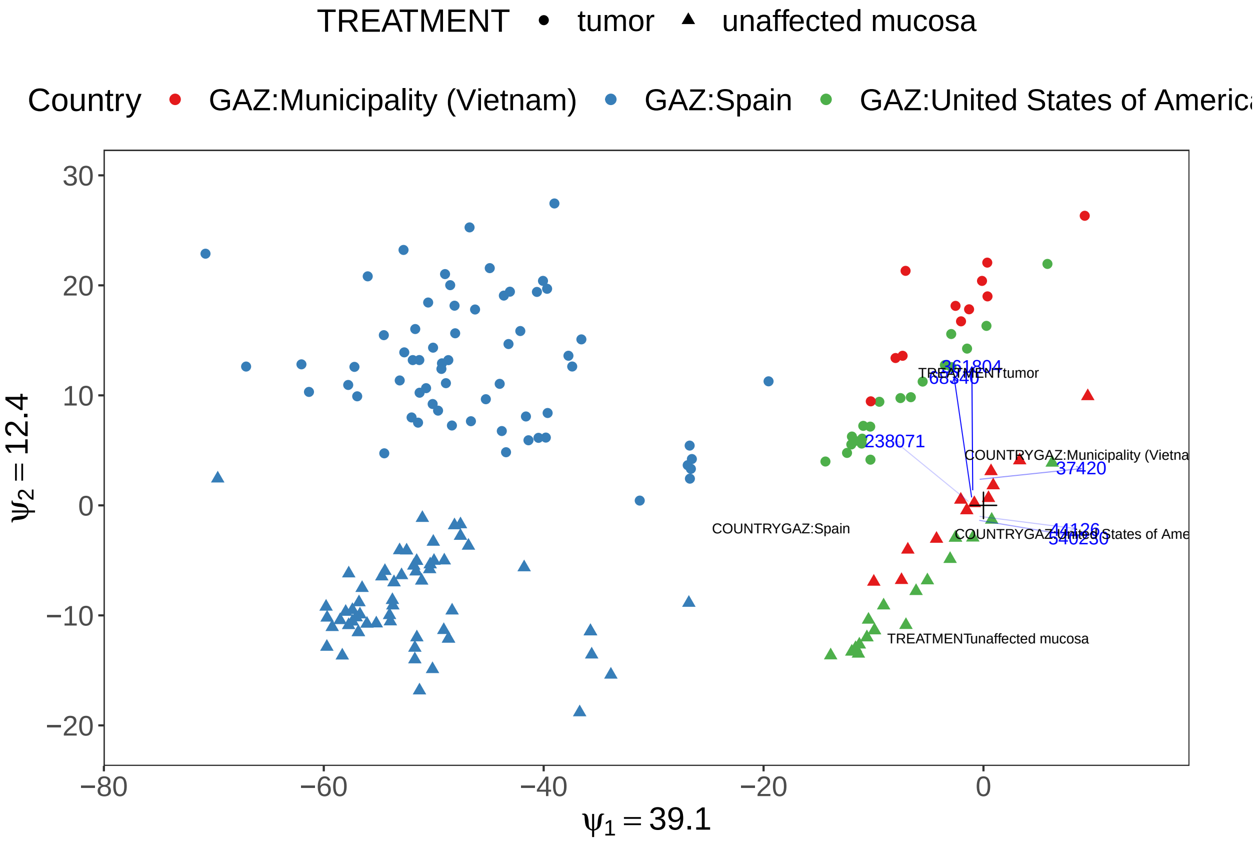

Figure S39: Constrained RC(M) triplot with linear response functions of Kostic data. In constrained RC(M), country of data collection and cancer status are clearly the main drivers of the ordination. For clarity only the most important variable is shown. , fig.height = 7

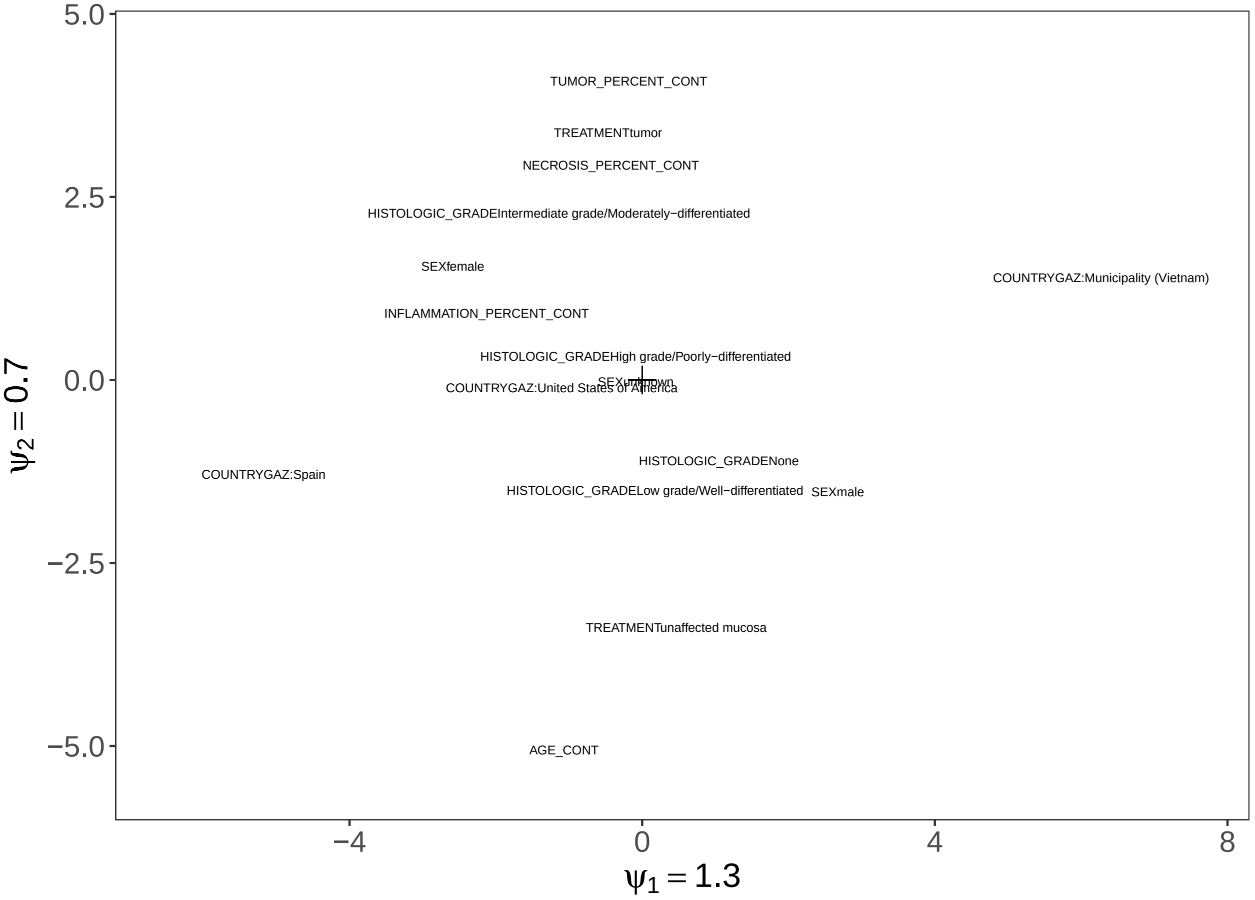

Figure S40: Constrained RC(M) monoplot with non-parametric response functions of Kostic data. Tumor percent stands out as an important variable

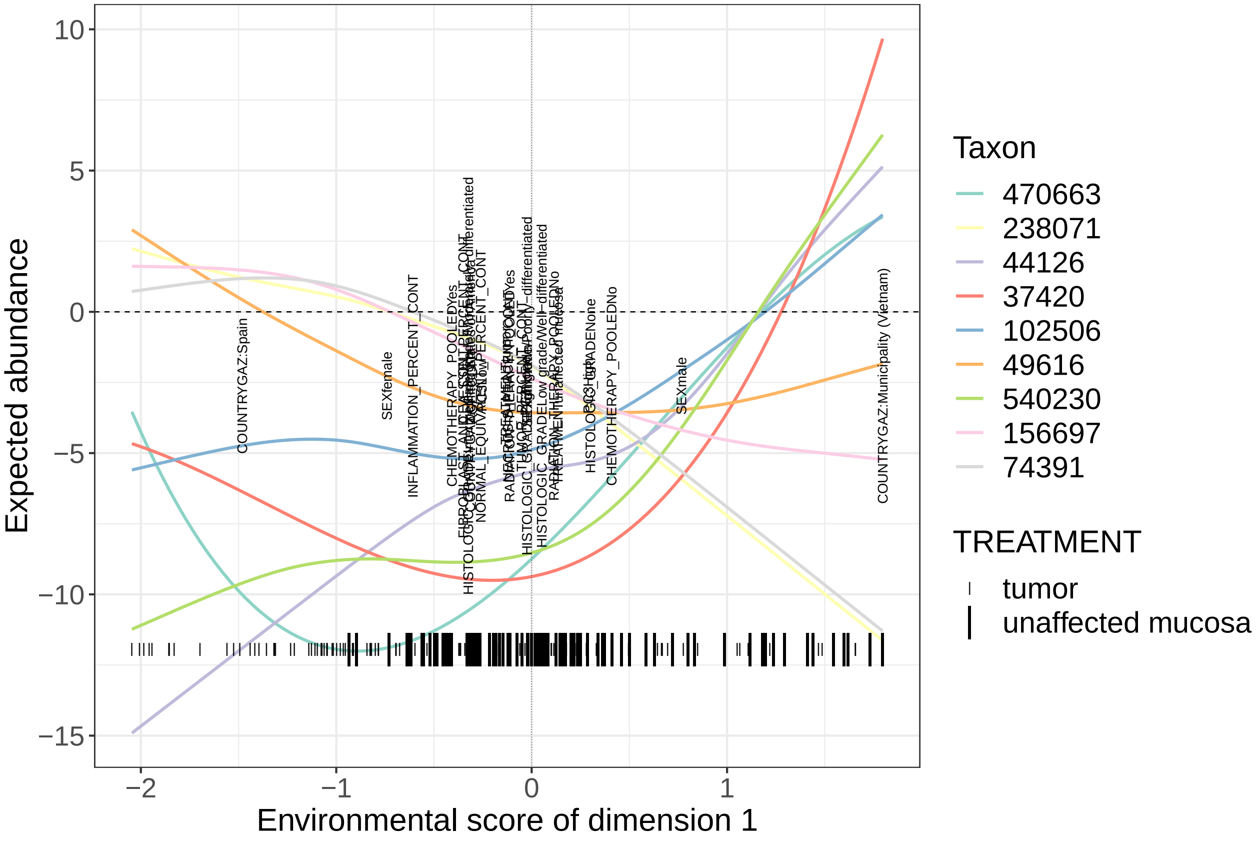

Figure S41: Non-parametric response functions of the constrained RC(M) triplot of Kostic data. The plot is largely dominated by one taxon with a very strong response

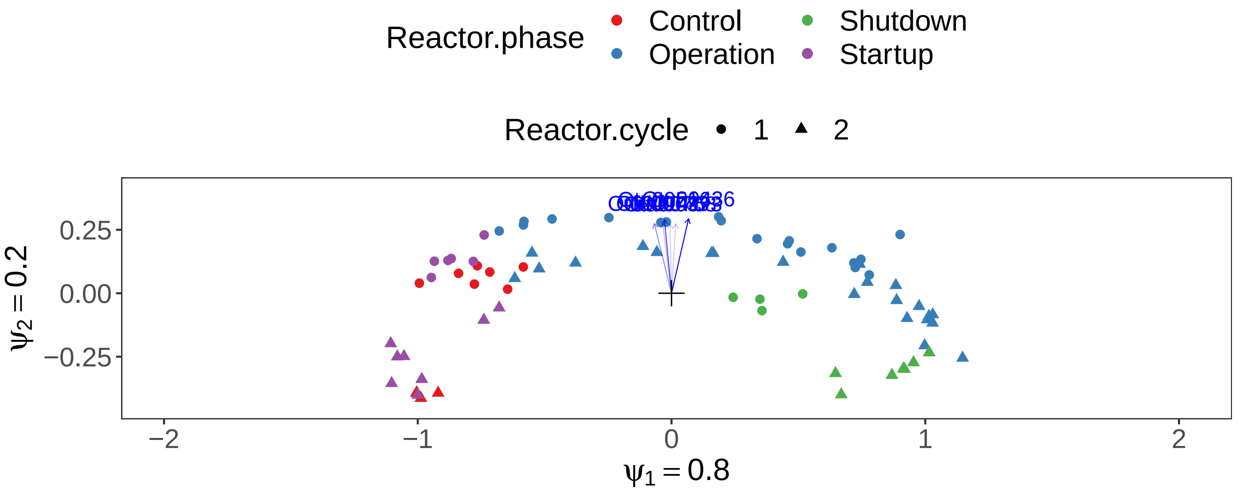

Figure S42: RC(M) biplot of the Props data. The first dimension mainly tracks the change of the cooling water throughout the different phases. There is a certain arch effect visible, which may indicate samples from the start up and shutdown are somewhat similar.

4.6 Props et al. (2016)

This is a longitudinal dataset on microbial growth in a water cooling system of a nuclear facility (Props et al. 2016).

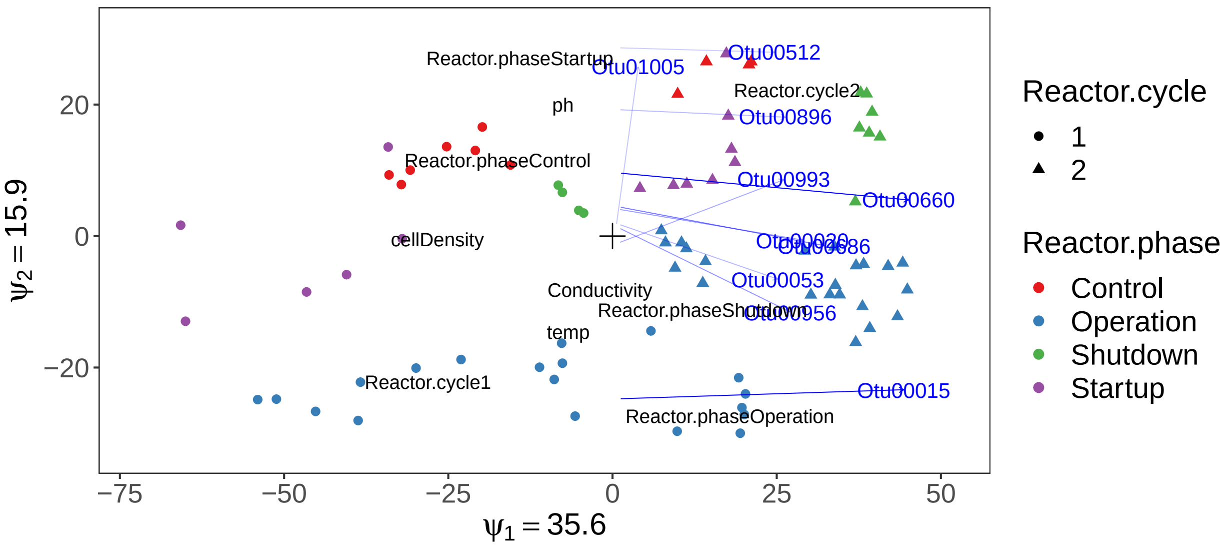

Figure S43: Constrained RC(M) triplot with linear response functions of the Props data. The constrained analysis confirms phase as being an important driver of sample variability. Also cycle turns out to be an important variable.

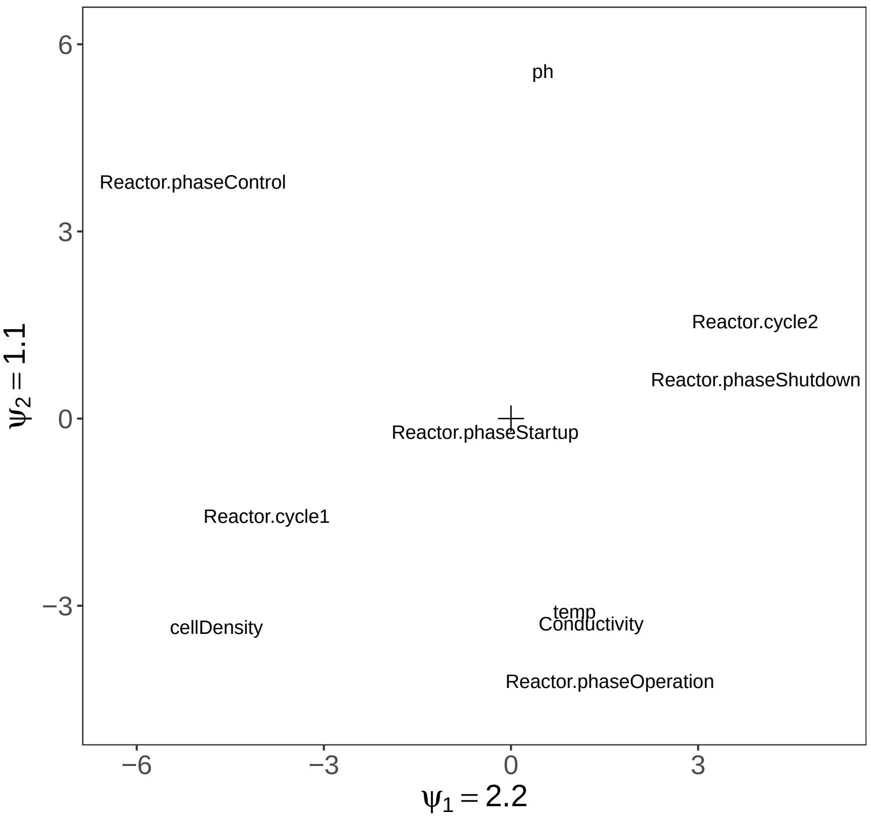

Figure S44: Constrained RC(M) monoplot with non-parametric response functions of the Props data. The constrained RC(M) with non-parametric response functions confirms the dominant role of reactor cycle and phase in shaping the ordination, although it allots more weight to pH as sample-specific variable.

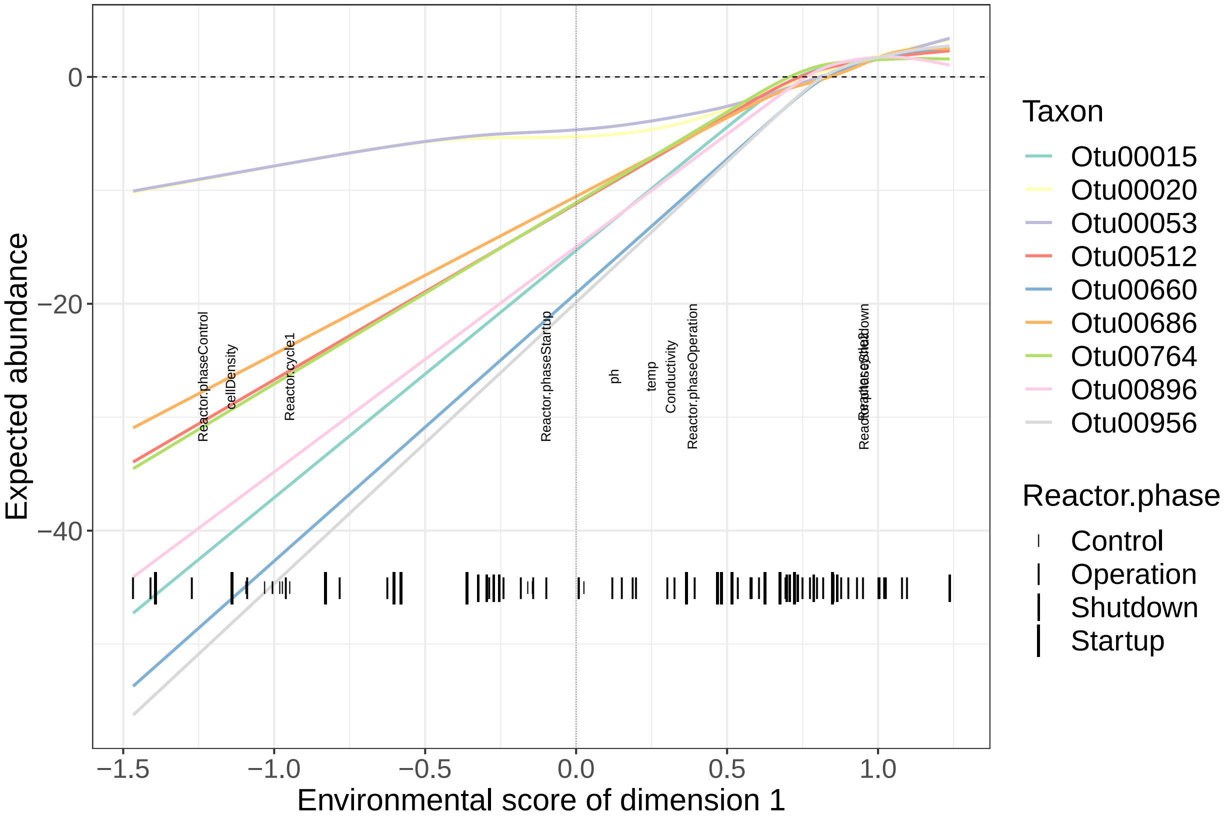

Figure S45: Response functions of constrained RC(M) with non-parametric response functions of the Props data. The response functions of the most strongly reacting taxa are monotonic in the first dimension, suggesting that a linear approximation may be appropriate. The samples clearly fall apart into two groups, which are well explained by reactor phase. This demonstrates the use of non-parametric response functions as a diagnostic for the linearity assumption for linear response functions.

5 R-code

All R-code used for the simulations and for generating the plots in the publication can be found in the S1 File. Code for fitting the RC(M) models can be found in the R-package *RCM* which can be downloaded from https://github.com/CenterForStatistics-UGent/RCM.

Existing implementations in R for fitting row-column interaction models, such as the *rcim*() function in the *VGAM* package (Yee 2015) and *rc*() in the *logmult* package (Bouchet-Valat 2017) fail to converge, likely due to numerical reasons.

6 R-language and package versions

All information on versions of the R-software and packages can be found in the following output of *sessionInfo()*.

```
## R version 3.5.1 (2018-07-02)
## Platform: x86_64-pc-linux-gnu (64-bit)
## Running under: Ubuntu 18.04.1 LTS
##
## Matrix products: default
## BLAS: /usr/lib/x86_64-linux-gnu/blas/libblas.so.3.7.1
## LAPACK: /usr/lib/x86_64-linux-gnu/lapack/liblapack.so.3.7.1
##
## locale:
##  [1] LC_CTYPE=en_US.UTF-8       LC_NUMERIC=C
##  [3] LC_TIME=en_GB.UTF-8        LC_COLLATE=en_US.UTF-8
##  [5] LC_MONETARY=en_GB.UTF-8    LC_MESSAGES=en_US.UTF-8
##  [7] LC_PAPER=en_GB.UTF-8       LC_NAME=C
##  [9] LC_ADDRESS=C               LC_TELEPHONE=C
## [11] LC_MEASUREMENT=en_GB.UTF-8 LC_IDENTIFICATION=C
##
## attached base packages:
## [1] stats     graphics  grDevices utils     datasets  methods   base
##
## other attached packages:
##  [1] xtable_1.8-3        zCompositions_1.1.2 truncnorm_1.0-8
##  [4] NADA_1.6-1          survival_2.43-3     MASS_7.3-51.1
##  [7] chron_2.3-53        boral_1.7           coda_0.19-2
## [10] logmult_0.7.0       gnm_1.1-0           gomms_1.0
## [13] gllvm_1.1.0         mvabund_3.13.1      TMB_1.7.15
## [16] cluster_2.0.7-1     ape_5.2             HMP_1.6
## [19] SpiecEasi_1.0.2     dirmult_0.1.3-4     tsne_0.1-3
## [22] reshape2_1.4.3      RCM_0.99.1          ggplot2_3.1.0
## [25] phyloseq_1.26.0
##
## loaded via a namespace (and not attached):
##  [1] nlme_3.1-137        bitops_1.0-6        xts_0.11-2
##  [4] RColorBrewer_1.1-2  rprojroot_1.3-2     numDeriv_2016.8-1
##  [7] tools_3.5.1         backports_1.1.2     R2WinBUGS_2.1-21
## [10] vegan_2.5-3         rpart_4.1-13        KernSmooth_2.23-15
## [13] lazyeval_0.2.1      BiocGenerics_0.28.0 mgcv_1.8-26
## [16] colorspace_1.3-2    nnet_7.3-12         permute_0.9-4
## [19] ade4_1.7-13         withr_2.1.2         curl_3.2
## [22] compiler_3.5.1      Biobase_2.42.0      alabama_2015.3-1
```

```
## [25] tseries_0.10-46     caTools_1.17.1.1    scales_1.0.0
## [28] mvtnorm_1.0-8       quadprog_1.5-5      stringr_1.3.1
## [31] digest_0.6.18       relimp_1.0-5        rmarkdown_1.10
## [34] XVector_0.22.0      pkgconfig_2.0.2     htmltools_0.3.6
## [37] rlang_0.3.0.1       TTR_0.23-4          rstudioapi_0.8
## [40] quantmod_0.4-13     huge_1.2.7          VGAM_1.0-6
## [43] zoo_1.8-4           jsonlite_1.5        gtools_3.8.1
## [46] magrittr_1.5        qvcalc_0.9-1        biomformat_1.10.0
## [49] Matrix_1.2-15       Rcpp_1.0.0          munsell_0.5.0
## [52] S4Vectors_0.20.1    Rhdf5lib_1.4.1      abind_1.4-5
## [55] stringi_1.2.4       yaml_2.2.0          nleqslv_3.3.2
## [58] zlibbioc_1.28.0     fishMod_0.29        rhdf5_2.26.0
## [61] gplots_3.0.1        plyr_1.8.4          grid_3.5.1
## [64] parallel_3.5.1      gdata_2.18.0        crayon_1.3.4
## [67] lattice_0.20-38     Biostrings_2.50.1   splines_3.5.1
## [70] multtest_2.38.0     tensor_1.5          knitr_1.20
## [73] pillar_1.3.0        igraph_1.2.2        boot_1.3-20
## [76] pulsar_0.3.4        codetools_0.2-15    stats4_3.5.1
## [79] R2jags_0.5-7        evaluate_0.12       rpart.plot_3.0.6
## [82] data.table_1.11.8   foreach_1.4.4       gtable_0.2.0
## [85] tweedie_2.3.2       rjags_4-8           tibble_1.4.2
## [88] iterators_1.0.10    IRanges_2.16.0      statmod_1.4.30
```

7 Hardware specifications

Real data analyses were run a Dell laptop with following specifications

- OS: Ubuntu 18.04 Bionic Beaver
- RAM: 16GB
- Processors: Intel i7 quadcore

Simulations were run on the high performance computing facilities of VSC (the Flemish Supercomputer Center) on the “delcatty cluster”, and on a server with following specifications:

- OS: Linux 8.6 (jessie)
- RAM: 132 GB
- Processors: Intel(R) Xeon(R) X7460 (12 Cores)

References

AmericanGut.org (2015). “The American gut project”. In: *https://github.com/biocore/American-Gut/blob/master/data/AG/AG_100nt.txt*.

Anders, S. and Huber, W. (2010). “Differential expression analysis for sequence count data”. In: *Genome Biol* 11.10. gb-2010-11-10-r106[PII], R106–R106.

Anderson, M. J. (2001). “A new method for non-parametric multivariate analysis of variance”. In: *Austral Ecology* 26.1, pp. 32 –46.

Becker, M. P. and Clogg, C. C. (1989). “Analysis of Sets of Two-Way Contingency Tables Using Association Models”. In: *Journal of the American Statistical Association* 84.405, pp. 142 –151.

Benidt, S. and Nettleton, D. (2015). “SimSeq: a nonparametric approach to simulation of RNA-sequence datasets”. In: *Bioinformatics* 31.13, pp. 2131–2140.

Bouchet-Valat, M. (2017). *Logmult: Log-Multiplicative Models, Including Association Models*. R package version 0.6.5.

Chen, J., Bittinger, K., Charlson, E. S., Hoffmann, C., Lewis, J., Wu, G. D., Collman, R. G., Bushman, F. D., and Li, H. (2012). “Associating microbiome composition with environmental covariates using generalized UniFrac distances”. In: *Bioinformatics* 28.16, pp. 2106 –2113.

Escoufier, Y. (1982). “L’analyse des tableaux de contingence simples et multiples”. In: *Metron* 40, pp. 53–77.

Goodman, L. (Sept. 1979). “Simple Models for the Analysis of Association in Cross-Classifications Having Ordered Categories”. In: 74, pp. 537 –552.

Hampel, F. R., Ronchetti, E. M., Rousseeuw, P. J., and Stahel, W. A. (2011). *Robust Statistics: The Approach Based on Influence Functions*. Vol. 07. John Wiley & Sons, Inc.

Heijden, P. G. M., Mooijaart, A., and Takane, Y. (Jan. 1994). “Correspondence analysis and contingency table models”. In:

Heijden, P. G. M. van der and Leeuw, J. de (1985). “Correspondence analysis used complementary to loglinear analysis”. In: *Psychometrika* 50.4, pp. 429–447.

Hui, F., Taskinen, S., Pledger, S., Foster, S., and Warton, D. (2015). “Model-based approaches to unconstrained ordination”. In: *Methods in Ecology and Evolution* 6.4, pp. 399 –411.

Kostic, A. D., Gevers, D., Pedamallu, C. S., Michaud, M., Duke, F., Earl, A. M., Ojesina, A. I., Jung, J., Bass, A. J., Tabernero, J., Baselga, J., Liu, C., Shivdasani, R. A., Ogino, S., Birren, B. W., Huttenhower, C., Garrett, W. S., and Meyerson, M. (2012). “Genomic analysis identifies association of Fusobacterium with colorectal carcinoma”. In: *Genome Res* 22.2. 22009990[Pmid], pp. 292 –298.

Kostic, A. D., Xavier, R. J., and Gevers, D. (2014). “The Microbiome in Inflammatory Bowel Diseases: Current Status and the Future Ahead”. In: *Gastroenterology* 146.6. 24560869[pmid], pp. 1489–1499.

Kurtz, Z. D., Müller, C. L., Miraldi, E. R., Littman, D. R., Blaser, M. J., and Bonneau, R. A. (May 2015). “Sparse and Compositionally Robust Inference of Microbial Ecological Networks”. In: *PLoS Comput Biol* 11.5, e1004226.

Oksanen, J., Blanchet, F. G., Friendly, M., Kindt, R., Legendre, P., McGlinn, D., Minchin, P. R., O'Hara, R. B., Simpson, G. L., Solymos, P., Stevens, M. H. H., Szoecs, E., and Wagner, H. (2017). *vegan: Community Ecology Package*. R package version 2.4-5.

Peterson, J., Garges, S., Giovanni, M., McInnes, P., Wang, L., Schloss, J. A., Bonazzi, V., McEwen, J. E., Wetterstrand, K. A., Deal, C., Baker, C. C., Di Francesco, V., Howcroft, T. K., Karp, R. W., Lunsford, R. D., Wellington, C. R., Belachew, T., Wright, M., Giblin, C., David, H., Mills, M., Salomon, R., Mullins, C., Akolkar, B., Begg, L., Davis, C., Grandison, L., Humble, M., Khalsa, J., Little, A. R., Peavy, H., Pontzer, C., Portnoy, M., Sayre, M. H., Starke-Reed, P., Zakhari, S., Read, J., Watson, B., and Guyer, M. (2009). "The NIH Human Microbiome Project". In: *Genome Res* 19.12. 19819907[pmid], pp. 2317–2323.

Pledger, S. and Arnold, R. (2014). "Multivariate methods using mixtures: Correspondence analysis, scaling and pattern-detection". In: *Computational Statistics & Data Analysis* 71.C, pp. 241 –261.

Props, R., Kerckhof, F.-M., Rubbens, P., De Vrieze, J., Hernandez Sanabria, E., Waegeman, W., Monsieurs, P., Hammes, F., and Boon, N. (2016). "Absolute quantification of microbial taxon abundances". In: *The ISME Journal* 11. Short Communication, pp. 584 –587.

R Core Team (2015). *R: A Language and Environment for Statistical Computing*. R Foundation for Statistical Computing. Vienna, Austria.

Robinson, M. D. and Smyth, G. K. (2007). "Moderated statistical tests for assessing differences in tag abundance". In: *Bioinformatics* 23.21, pp. 2881–2887.

Robinson, M. D., McCarthy, D. J., and Smyth, G. K. (2010). "edgeR: a Bioconductor package for differential expression analysis of digital gene expression data". In: *Bioinformatics* 26.1. btp616[PII], pp. 139–140.

Rousseeuw, P. J. (1987). "Silhouettes: A graphical aid to the interpretation and validation of cluster analysis". In: *Journal of Computational and Applied Mathematics* 20.Supplement - C, pp. 53 –65.

Schmidt, T. S. B., Rodrigues, J. F. M., and Mering, C. von (2016). "A family of interaction-adjusted indices of community similarity". In: *The Isme Journal* 11.3, pp. 791–807.

Sohn, M. B. and Li, H. (2017). "A GLM-based latent variable ordination method for microbiome samples". In: *Biometrics*, e–pub ahead of print.

ter Braak, C. J. F. (1986). "Canonical Correspondence Analysis: A New Eigenvector Technique for Multivariate Direct Gradient Analysis". In: *Ecology* 67.5, pp. 1167 –1179.

Turnbaugh, P. J., Ridaura, V. K., Faith, J. J., Rey, F. E., Knight, R., and Gordon, J. I. (2009). "The Effect of Diet on the Human Gut Microbiome: A Metagenomic Analysis in Humanized Gnotobiotic Mice". In: *Sci Transl Med* 1.6. 20368178[Pmid], 6ra14 –6ra14.

Yee, T. W. (2006). "Constrained additive ordination". In: *Ecology* 87.1, pp. 203 –213.

Yee, T. W. (2015). *Vector Generalized Linear and Additive Models: With an Implementation in R*. New York, USA: Springer.

Yee, T. W. and Hadi, A. F. (2014). "Row–column interaction models, with an R implementation". In: *Computational Statistics* 29.6, pp. 1427 –1445.

Zeller, G., Tap, J., Voigt, A. Y., Sunagawa, S., Kultima, J. R., Costea, P. I., Amiot, A., Böhm, J., Brunetti, F., Habermann, N., Hercog, R., Koch, M., Luciani, A., Mende, D. R., Schneider, M. A., Schrotz-King, P., Tournigand, C., Tran Van Nhieu, J., Yamada, T., Zimmermann, J., Benes, V., Kloor, M., Ulrich, C. M., Knebel Doeberitz, M. von, Sobhani, I., and Bork, P. (2014). "Potential of fecal microbiota for early-stage detection of colorectal cancer". In: *Mol Syst Biol* 10.766. 25432777[pmid].

Zhang, Y. and Thas, O. (Apr. 2016). "Constrained Ordination Analysis with Enrichment of Bell-Shaped Response Functions". In: *PLOS ONE* 11.4, pp. 1 –21.

Zhu, M., Hastie, T., and Walther, G. (Feb. 2005). "Constrained ordination analysis with flexible response functions". In: *Ecological Modelling* 187, pp. 524–536.

Zwilling, M. L. (2013). "Negative Binomial Regression". In: *The Mathematica Journal*, pp. 15 –16.
